# Bridging Pharmacopeial Standards and Chemical Reality of Triterpene Glycosides in *Actaea racemosa*

**DOI:** 10.3390/plants15152272

**Published:** 2026-07-24

**Authors:** Larisa-Mălina Cătăuță, Roman Senn, Alexander Schenk, Amin Chaanin, Christiane Halbsguth, Ombeline Danton

**Affiliations:** 1 Max Zeller Söhne AG, Seeblickstrasse 4, 8590 Romanshorn, Switzerland; larisa.catauta@zellerag.ch (L.-M.C.); roman.senn@zellerag.ch (R.S.); aschenk58@icloud.com (A.S.); ombelined@gmail.com (O.D.); 2 VitaPlant AG, Romanshornerstrasse 39, 8592 Uttwil, Switzerland; achaanin58@icloud.com

**Keywords:** triterpene glycosides, *Cimicifuga racemosa* Nutt., *Actaea racemosa*, *European Pharmacopoeia*, HPLC–ELSD method, wild plant material, cultivation

## Abstract

*Actaea racemosa* L. (syn. *Cimicifuga racemosa* (L.) Nutt., black cohosh) rhizomes contain triterpene glycosides (TTGs) that serve as principal quality markers in the *European Pharmacopoeia* (Ph. Eur.) monograph. However, discrepancies between chromatographic profiles of the plant material and the herbal reference standard (HRS) dry extract complicate quality assessment. This study elucidated the chemical origin of these discrepancies and evaluated whether the extract-based HRS can represent the analytical fingerprint of the plant material. Thirteen TTGs were identified and characterized within the official Ph. Eur. HPLC–ELSD method using centrifugal partition chromatography, semi-preparative UPLC, HRMS, GC–MS and NMR spectroscopy, employing reference standards and isolated compounds. Three malonylated TTGs (**8a**, **9a**, **10a**) were detected in wild and cultivated rhizomes but were absent from the *A. racemosa* dry extract HRS and other dry extracts. Quantitative analysis revealed that cultivated plants contained an insufficient amount of TTGs; however, after extraction, both the amount and the profile aligned with the Ph. Eur. requirements. This behavior is explained by the degradation and rearrangement pathways of the TTGs. Overall, these findings clarify the origin of chemical variability between plant and extract fingerprints and support the inclusion of malonylated TTGs in the quantitative determination of *A. racemosa* triterpene glycosides in the Ph. Eur.

## 1. Introduction

*Actaea racemosa* L. (syn. *Cimicifuga racemosa* (L.) Nutt.), commonly known as black cohosh, is a perennial plant that belongs to the Ranunculaceae family, native to the deciduous forests of eastern North America [1]. While the herbal drug consists of both rhizomes and roots of *A. racemosa* L., the term “rhizome” is used throughout this text, following the *European Pharmacopoeia* (Ph. Eur.), where the drug is officially defined as *Cimicifugae rhizoma* [2]. Preparations obtained from *A. racemosa* rhizomes are widely used for the relief of climacteric symptoms and as an alternative to hormone replacement therapy [3].

Commercial dependence on wild-harvested rhizomes raises both conservation and supply concerns [4,5]. The conservation status of the species varies across its North American range, with populations classified as imperiled in some jurisdictions, while being ranked as secure in others [6]. Because harvesting targets the roots and rhizomes, repeated or intensive collection may impair population regeneration, with experimental studies indicating that recovery can require several years [5,7]. Cultivation and forest farming have therefore been proposed as complementary strategies to reduce pressure on wild populations and improve the reliability of the supply chain [5].

In addition to these conservation concerns, correct identification of *A. racemosa* represents an important quality-control challenge. Adulteration or contamination of *A. racemosa* species with imported Asian species, such as *Actaea cimicifuga* (syn. *Cimicifuga foetida*), *A. dahurica* (syn. *C. dahurica*), *A. heracleifolia* (syn. *C. heracleifolia*), *A. simplex* (syn. *C. simplex*), and *A. brachycarpa* (syn. *C. brachycarpa*), as well as with North American Actaea species (*A. pacypoda*, *A. rubra* and *A. podocarpa*), has frequently been reported [8].

Chemically, the two main classes of substances present in the rhizomes are triterpene glycosides (TTGs) and phenolic compounds [1]. Even though clarifications on the mode of action of TTGs are still ongoing, they are used as markers for the quality of the herbal drug, and in some cases to identify falsification or contamination [8,9,10]. Cimiracemoside C (syn. cimigenol-3-*O*-α-L-arabinopyranoside) is considered a specific marker of *A. racemosa* [11,12], since a study based on HPLC analysis reported its presence in *A. racemosa* (and *A. rubifolia*) and its absence in *A. podocarpa*, *A. acerina*, *A. biternata*, *A. dahurica*, *A. foetida*, *A. heracleifolia*, *A. japonica* and *A. simplex* [13]. However, cimiracemoside C (syn. cimigenol-3-*O*-α-L-arabinopyranoside) was isolated from *A. dahurica* (Turcz. ex Fisch. & C.A.Mey.) Maxim as early as 1999 [14], and from *A. cimicifuga* L. in 2007 [15]. On the other hand, cimifugin has so far not been found in *Actaea racemosa*, and is currently used as a falsification marker in several publications [8,10] and in the HPTLC method from the *European Pharmacopoeia* monograph of black cohosh (01/2025:2069) [2].

In Europe, in order to commercialize black cohosh-based medicines, the rhizomes must comply with the criteria set by the Ph. Eur. The previous version (04/2014:2069) relied on a high-performance thin-layer chromatography (HPTLC) method comparing plant samples to an *A. racemosa* drug as an herbal reference standard (HRS) and reference solutions of actein and cimifugin [16]. In the current version (01/2025:2069), the identification is based exclusively on reference substances (actein, 23-*epi*-26-deoxyactein, isoferulic acid, cimifugin) instead of a comparison with the *A. racemosa* drug HRS [2].

Quantitative analysis of TTGs in the plant material is performed by high-performance liquid chromatography with evaporative light-scattering detection (HPLC–ELSD), using a dry extract of *A. racemosa* as HRS, as required by the Ph. Eur. A fingerprint of the *A. racemosa* dry extract HRS is provided, with 12 specified peaks, whose areas must be summed to obtain a minimum content of 1% TTGs [2,16].

Against this background, cultivated *A. racemosa* was investigated as a potential alternative source of herbal drug material. During preliminary quality assessment, the investigated cultivated rhizomes showed TTG profiles that did not fully correspond to the profile of the *A. racemosa* dry extract HRS, whereas the corresponding dry extracts displayed the expected pharmacopeial profile. These observations raised the broader question of whether the *A. racemosa* dry extract HRS adequately reflects the TTG profile of the *A. racemosa* herbal drug.

Previous studies on TTG conversion have shown that the temperature conditions during extract preparation or drying of the plant material strongly influence the resulting chromatographic fingerprint [13,17]. Based on these findings, we hypothesized that the differences observed between the cultivated rhizomes and their corresponding dry extracts are, at least in part, attributed to compositional changes occurring during extraction and drying. To test this hypothesis, the HPLC profiles of plant materials and dry extracts were first compared. Key peaks of the official profile of *A. racemosa* dry extract HRS, along with additional relevant peaks, were identified using reference standards or by isolation followed by structure elucidation (NMR, HR–MS). Chromatographic variability among individual wild plants was assessed, followed by a comparison of wild and cultivated batches. Finally, cultivated plants were evaluated against their corresponding dry extracts to determine compositional changes introduced during extraction and drying.

## 2. Results

### 2.1. Comparison of UPLC-ELSD and HPLC-ELSD Profiles of TTGs in A. racemosa Drug and Dry Extract HRS

**Figure 1:**
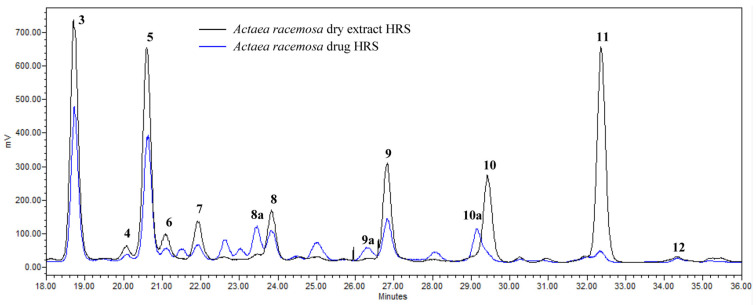
UPLC–ELSD chromatogram of *Actaea racemosa* dry extract HRS and *Actaea racemosa* drug HRS.

**Figure 2:**
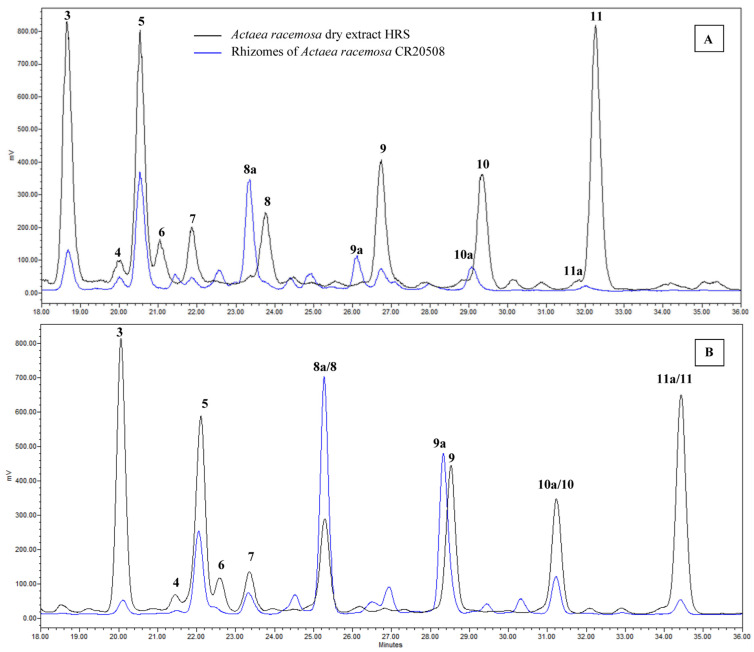
UPLC–ELSD (**A**) and HPLC–ELSD (**B**) chromatograms of a rhizome of *Actaea racemosa* (CR20508) and *Actaea racemosa* dry extract HRS.

According to the Ph. Eur. monograph, a dry extract is used as the HRS for the quantification of TTGs in plant material [2,16]. Therefore, it was necessary to assess how the TTG profile of the plant material differs from that of the dry extract. For this purpose, *A. racemosa* drug HRS and the *A. racemosa* dry extract HRS were analyzed by UPLC–ELSD with method parameters according to the Ph. Eur. (Figure 1). Strong variations in profiles were observed between both HRSs. The *A. racemosa* drug HRS contains peaks eluting shortly before peaks **8**, **9**, **10** and **11** (defined by the *A. racemosa* dry extract HRS). These peaks were named **8a**, **9a**, **10a** and **11a**, respectively. It is relevant to note that when using an HPLC instrument, the separation between peaks **8a**/**8**, **9a**/**9**, **10a**/**10** and **11a**/**11** is lost (Figure 2), resulting in a lack of information. For this reason, only a UPLC instrument with the same method parameters as described in the Ph. Eur. monograph was used further [2,16].

### 2.2. Identification of TTGs by Reference Standards and Structural Elucidation of Isolated Compounds

To determine the identities of the 12 peaks specified in the Ph. Eur. assay, as well as those of the additional peaks **8a**, **9a**, **10a** and **11a**, commercially available TTG standards were analyzed by UPLC–ELSD and by UPLC–ESI–QTOF (+). Seven out of these 12 peaks were identified in the *A. racemosa* dry extract HRS by comparison with reference standards: cimiracemoside A (**1**, HRESIMS *m*/*z* 699.3671 [M+Na]^+^), 24-*O*-acetylhydroshengmanol-3-*O*-β-D-xylopyranoside (**9**, HRESIMS *m*/*z* 703.3945 [M+Na]^+^), cimigenol-3-*O*-α-L-arabinoside (**10**, HRESIMS *m*/*z* 643.3763 [M+Na]^+^) and cimigenol-3-*O*-β-D-xyloside (**11**, HRESIMS *m*/*z* 643.3765 [M+Na]^+^). Actein, available as a mixture of 23-R/23S-epimers, revealed two peaks: one corresponding to peak 3 (named **3a** to distinguish from **3b**, both compounds eluting at the retention time of peak 3) and a second one corresponding to peak 5 (named **5a** to distinguish from **5b**, both compounds eluting at the retention time of peak 5). 23-*epi*-26-deoxyactein also eluted at the retention time of peak 3 (named **3b**, HRESIMS *m*/*z* 683.3707 [M+Na]^+^). The MS spectra of peak 3 of the *A. racemosa* dry extract HRS confirmed the presence of both substances. Additionally, cimigenol aglycone was detected at the RT of 42.3 min (named **14**, HRESIMS *m*/*z* 511.3434 [M+Na]^+^).

Compounds **5b** (eluting at the retention time of peak 5), **8a**, **9a**, **10a** and **13** were isolated by semi-preparative HPLC from the rhizomes of *A. racemosa*. Compound **11a** could not be isolated in the amount and purity required for NMR analysis. Based on HRESIMS data, its molecular mass was estimated to be approximately 852 Da. In comparison with compound **10a**, the observed 86 Da mass increment is consistent with the presence of an additional malonyl group on the sugar moiety. Compound **9a** was shown to lose one or both malonyl groups during degradation. By analogy, the putative compound **11a** may be particularly labile. If **11a** represents a dimalonylated analog of compound **10a**, it would combine a malonyl-rich sugar moiety with an acetylhydroshengmanol-type aglycone, a structural type involved in the conversion sequence toward cimigenol glycosides. This combination may have contributed to its apparent instability and to the difficulty of isolating sufficient material for NMR-based structural confirmation.

The structures of the isolated TTGs were established by combined analysis of 1D and 2D NMR data, including ^1^H NMR, ^13^C-DEPTq where available, ^1^H–^1^H COSY, ROESY, HSQC-DEPT and HMBC spectra, together with HRESIMS data. The complete ^1^H and ^13^C NMR assignments are provided in Table S1 in the Supplementary Materials; the corresponding NMR spectra are shown in Figures S1–S33, and the HRESIMS spectra in Figures S34–S39.

The isolated compounds showed characteristic NMR features of C-3 glycosylated triterpenes, including oxygenated C-3 resonances at δC 87.4–87.9 ppm and anomeric H-1′/C-1′ signals of the sugar moiety, correlating together in HMBC.

Compounds **5b**, **8a** and **9a** displayed closely related NMR profiles, consistent with a 23-*O*-acetylshengmanol aglycone, as indicated by the acetyl carbonyl resonance, labeled Ac1 in Table S1 (δC 170.0–170.3 ppm), the acetyl methyl resonance, labeled Ac2 in Table S1 (δH 2.03–2.09 ppm), and the characteristic downfield-shifted H-23 signal (δH 4.81–4.89 ppm) with the corresponding C-23 resonance at δC 71.2–71.8 ppm. GC–MS analysis after acid hydrolysis confirmed D-xylose as the sugar moiety in compounds **5b**, **8a** and **9a.** In comparison with the non-malonylated compound **5b**, compound **8a** showed a pronounced downfield shift in H-2′ from δH 3.06 to δH 4.52 ppm, whereas H-4′ remained essentially unchanged. Together with the presence of one set of malonyl carbonyl and methylene resonances, this supported malonylation at O-2′. Compound **9a** showed a similar downfield-shifted H-2′ signal at δH 4.54 ppm and an additional downfield shift in H-4′ at δH 4.58 ppm, together with a second set of malonyl resonances, consistent with a 2′,4′-*O*-dimalonylated derivative. Therefore, they were identified as 23-*O*-acetylshengmanol-3-*O*-β-D-xylopyranoside (**5b**), 23-*O*-acetylshengmanol-3-*O*-(2′-*O*-malonyl)-β-D-xylopyranoside (**8a**), and 23-*O*-acetylshengmanol-3-*O*-(2′,4′-*O*-dimalonyl)-β-D-xylopyranoside (**9a**).

Compound **10a** was distinguished from compounds **5b**, **8a** and **9a** by the resonances in the C-23/C-24 side-chain region. In contrast to the 23-*O*-acetylshengmanol derivatives, compound **10a** showed a downfield-shifted H-24 signal at δH 4.83 ppm with the corresponding C-24 resonance at δC 81.3 ppm, together with acetyl resonances at δC 170.6 ppm and δH 2.00 ppm, supporting its assignment as a 24-*O*-acetylhydroshengmanol derivative. GC–MS analysis after acid hydrolysis confirmed D-xylose as the sugar moiety. The downfield-shifted H-2′ signal at δH 4.41 ppm, together with one set of malonyl carbonyl and methylene resonances, further supported malonylation at O-2′. The structure was thus established as 24-*O*-acetylhydroshengmanol-3-*O*-(2′-*O*-malonyl)-β-D-xylopyranoside (**10a**).

Compound **13** showed an NMR profile consistent with a related shengmanol-type aglycone. Compared with compounds **5b**, **8a** and **9a**, it lacked the acetyl resonances Ac1/Ac2 and the characteristic downfield-shifted H-23 signal of the 23-*O*-acetylated derivatives. Moreover, sugar analysis by GC–MS and NMR of the sugar moiety were comparable to those of the non-malonylated compound **5b**, and no malonyl resonances were detected. These data supported its identification as shengmanol-3-*O*-β-D-xylopyranoside (**13**).

The MS spectrum of peak 8 (**8**, HRESIMS *m*/*z* 703.3958 [M+Na]^+^) closely resembled that of **9** (HRESIMS *m*/*z* 703.3945 [M+Na]^+^), suggesting a related structure differing in the sugar moiety, analogous to the arabinoside/xyloside pair observed for **10** and **11**. However, compound **8** could not be isolated in sufficient amount and purity due to its instability. Upon thermal degradation, peak 8 yielded compound **8b** (HRESIMS *m*/*z* 661.3865 [M+Na]^+^), whose NMR data supported a 24-*O*-hydroshengmanol-type aglycone. Compound **8b** showed no acetyl resonances, and the 42 Da mass difference between peak 8 and compound **8b** was consistent with loss of an acetyl substituent during degradation.

**Scheme 1:**
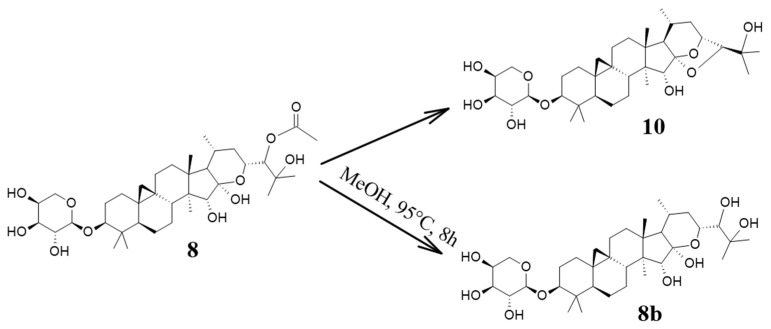
Experimentally observed thermal conversion of compound **8** into compounds **10** and **8b**.

GC–MS analysis after acid hydrolysis confirmed L-arabinose as the sugar moiety in compound **8b**. Based on the mass data, degradation behavior, NMR characterization of **8b** and GC–MS sugar analysis, peak 8 was assigned as 24-*O*-acetylhydroshengmanol-3-*O*-α-L-arabinopyranoside (**8**, Scheme 1).

Based on HRESIMS data, peaks 2, 6 and 7 showed a fragmentation pattern closely related to 23-*epi*-26-deoxyactein, with characteristic ions at *m*/*z* 451, 469, 601, 661. These findings suggest that these peaks may correspond to closely related cycloartane-type triterpene glycosides. For peaks 2 and 6, the ion at *m*/*z* 701, consistent with [M+Na]^+^, indicates a molecular mass of approximately 678 Da, i.e., 18 Da higher than 23-*epi*-26-deoxyactein. The common ion at *m*/*z* 661 is therefore interpreted as an in-source dehydrated ion [M+H−H_2_O]^+^, explaining the close similarity of the subsequent fragment ions to those of 23-*epi*-26-deoxyactein. These peaks may represent deoxyactein-related triterpene isomers, potentially including xyloside/arabinoside isomeric forms. Peak 7 displayed a similar fragmentation pattern but appeared less clearly resolved, suggesting either closely related isomeric triterpene glycosides or partial co-elution, possibly involving components with molecular masses of approximately 678 and 680 Da.

Peak 4 showed a sodium adduct ion at *m*/*z* 685.3860 [M+Na]+, corresponding to a molecular mass of approximately 662 Da and matching the molecular ion of isolated 23-*O*-acetylshengmanol-3-*O*-β-D-xylopyranoside (**5b**). The shared ion at *m*/*z* 435 further supports a structurally related acetylshengmanol-type triterpene glycoside. Additional ions in peak 4, such as *m*/*z* 645, 585 and 453, are consistent with dehydration and loss of acetic acid from an acetylated triterpene glycoside. Therefore, peak 4 was tentatively assigned to an acetylshengmanol arabinopyranoside, corresponding to the arabinoside analog of compound **5b**.

Peak 12 exhibited a sodium adduct ion at *m*/*z* 729.3720 [M+Na]^+^, corresponding to a molecular mass of approximately 706 Da. This mass is 86 Da higher than that of compounds **10**, **11** and **13**, consistent with the presence of one additional malonyl group. The ion at *m*/*z* 643 further supports this interpretation, as it corresponds to the loss of a malonyl residue and matches the sodium adduct of non-malonylated cimigenol-/shengmanol-type glycosides represented by compounds **10**, **11** and **13**. Moreover, the fragment ions at *m*/*z* 585, 453 and 435 closely resemble those observed for cimigenol-3-*O*-α-L-arabinoside (**10**), cimigenol-3-*O*-β-D-xyloside (**11**) and shengmanol-3-*O*-β-D-xylopyranoside (**13**). However, since these compounds have the same molecular mass and closely related HRESIMS fragmentation patterns, the available MS data do not allow discrimination between a malonylated cimigenol-type and a malonylated shengmanol-type glycoside. Therefore, peak 12 was tentatively classified as a malonylated cimigenol-/shengmanol-type triterpene glycoside.

**Figure 3:**
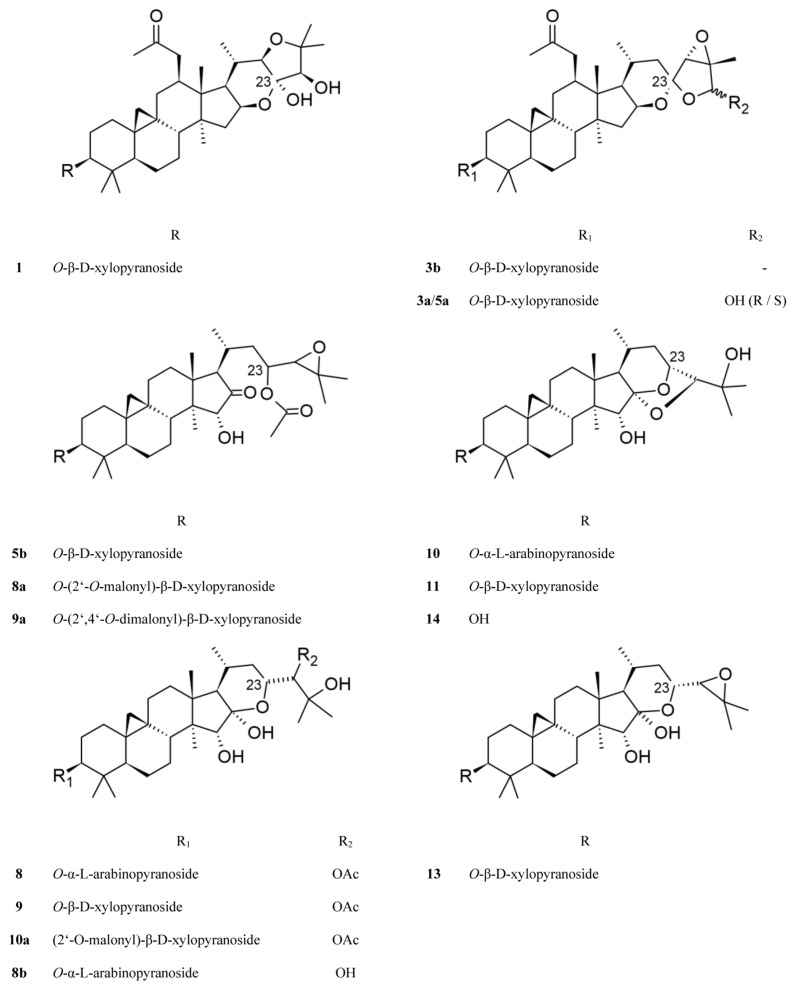
Chemical structures of the TTGs identified in the rhizomes and extracts of *Actaea racemosa*. The numbering of the compounds (**1**–**12**) corresponds to the peak numbering in the *Actaea racemosa* dry extract HRS chromatogram. Compounds **3a**/**3b** and **5a**/**5b** represent co-eluting species associated with peaks 3 and 5, respectively. Peaks 2, 4, 6, 7 and 12 could not be unambiguously assigned to individual compounds. The remaining annotated compounds (**8a**, **8b**, **9a**, **10a**, **13**, **14**) were identified beyond the main Ph. Eur. peaks.

Due to the absence of authentic reference substances and the limited diagnostic value of HRESIMS fragmentation for distinguishing closely related triterpene glycoside isomers, peaks 2, 4, 6, 7 and 12 could not be unambiguously identified at the individual compound level. Nevertheless, their mass spectral characteristics support their classification as structurally related cycloartane-type triterpene glycosides. An overview of all confirmed and tentative assignments, together with the corresponding sources of evidence, is provided in Table A1. The chemical structures of the TTGs that were unambiguously assigned using reference standards or isolated compounds are shown in Figure 3.

### 2.3. Degradation Pathways of TTGs Observed During Isolation

The instability of TTGs during isolation posed significant challenges for obtaining pure compounds. To minimize degradation, the fractions were lyophilized immediately after collection with the Waters Fraction Manager-Analytical (WFM-A), and subsequently stored at −20 °C.

**Scheme 2:**
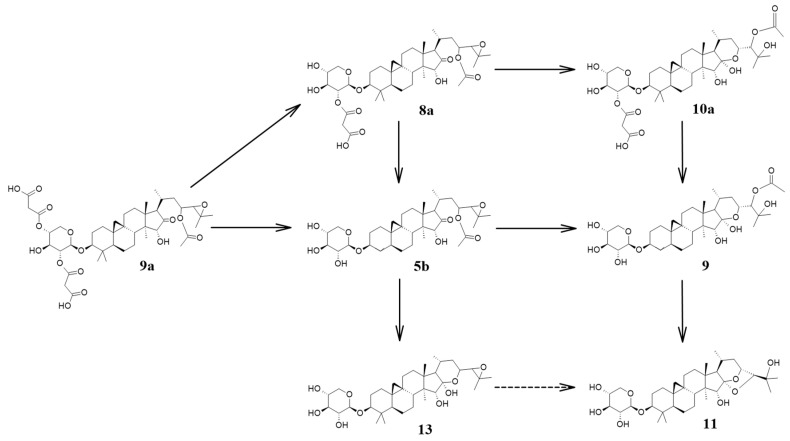
Experimentally observed and proposed conversion pathways of the TTGs identified during storage and isolation. Solid arrows indicate experimentally observed transformations, whereas the dashed arrow from compound **13** to compound **11** indicates a theoretically proposed conversion that was not directly observed in this study.

The lyophilized fractions were evaluated for purity by UPLC–MS, and those showing minimal degradation were combined to yield sufficient material for NMR analysis. Based on the analysis of degraded samples, conversion pathways were proposed, as summarized in Scheme 2. The rationale behind these degradation processes was explained by Kusano [13,17], who demonstrated that acetylshengmanol glycoside serves as a precursor for hydroshengmanol and cimigenol glycosides. Another aspect involved the instability of the malonylated glycosides of **8a**, **9a** and **10a**, which readily converted into their corresponding non-malonylated glycosides. Specifically, **8a** degraded into **5b**, **9** and **10a**. Similarly, **9a** yielded **5b**, **8a**, **9**, and **10a**. Compound **5b** showed minor amounts of **9** and **13**, whereas **13** proved to be the most stable among the isolated compounds.

Compound **9a** readily loses one or both malonyl groups, converting to **8a** and **5b**, respectively. Compound **9** could be produced either by **5b** through conversion from acetylshengmanol to acetylhydroshengmanol type [13,17], or by the loss of malonyl group of **10a**. Compound **13** was detected as a degradation product of **5b**; the conversion is proposed to involve deacetylation at C-23 followed by the first cyclization to a shengmanol type while retaining the epoxy group. In analogy to the reported chemical conversion [13,17], **13** could theoretically undergo a second cyclization to yield compound **11**, although this transformation was not directly observed in this study.

Furthermore, migration of acyl substituents within carbohydrate moieties is well documented [18]. Consistent with this behavior, intramolecular acyl rearrangements within the sugar moieties of compounds **9a** and **8a** were observed. Two degradation products of **9a** exhibited identical MS fragmentation patterns (*m*/*z* 817, 857, 873) but distinct retention times, suggesting positional isomers with the malonyl groups relocated to the 2′,3′- and 3′,4′-position of the sugar ring (Figure S43, Supporting Information). In degraded samples of **8a**, positional malonyl isomers (3′- and 4′-) and compound **9a** were detected (Figure S41, Supporting Information). Compound **9a** likely forms by further acylation of **8a**, involving free or activated malonyl intermediates generated during partial hydrolysis and subsequent re-esterification of the sugar hydroxyl groups.

### 2.4. TTG Profile Variability in Wild and Cultivated Actaea racemosa Rhizomes

**Figure 4:**
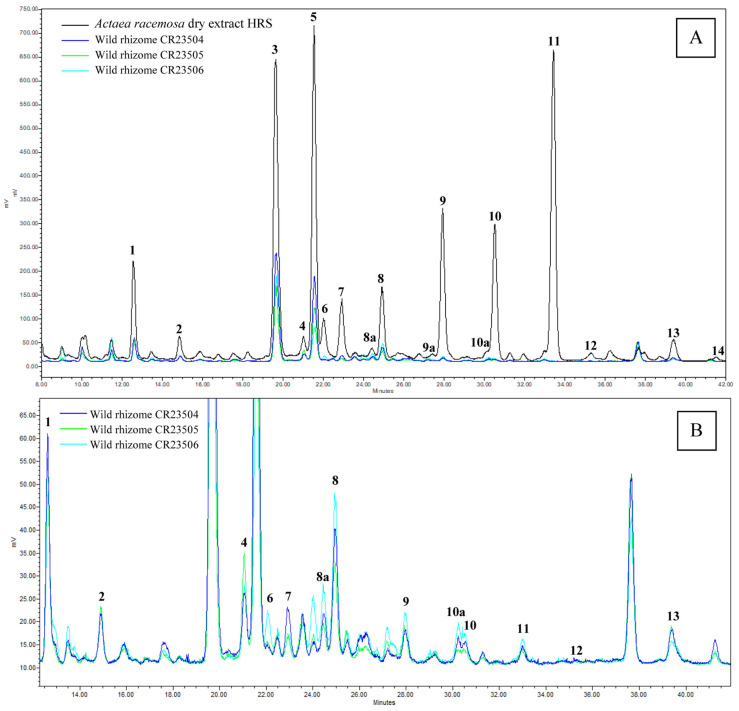
UPLC–ELSD fingerprints of individually collected wild rhizomes of *Actaea racemosa* (CR23504, CR23505, CR23506) compared with *Actaea racemosa* dry extract HRS (**A**) and zoomed in (**B**).

**Figure 5:**
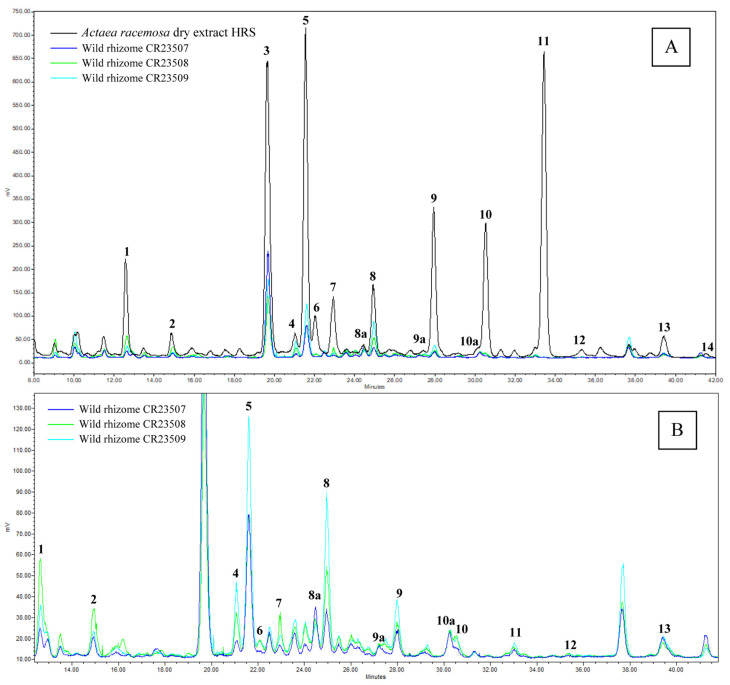
UPLC–ELSD fingerprints of individually collected wild rhizomes of *Actaea racemosa* (CR23507, CR23508, CR23509) compared with *Actaea racemosa* dry extract HRS (**A**) and zoomed in (**B**).

After establishing the identity and conversion behavior of the major TTGs, variability among individual plants was examined. For this purpose, rhizomes from individual wild plants of *A. racemosa* (CR23504–CR23509) were harvested, dried separately, and analyzed by UPLC–ELSD using the chromatographic conditions described in the Ph. Eur. monograph (Figure 4 and Figure 5). The chromatographic profiles of individual plants differed mainly in peak intensities, but all contained both **8** and **8a**, as well as **9** and **9a**, with overlapping peaks **10** and **10a** in all samples.

To explore sustainable alternatives, rhizomes from cultivated *A. racemosa* were analyzed.

**Figure 6:**
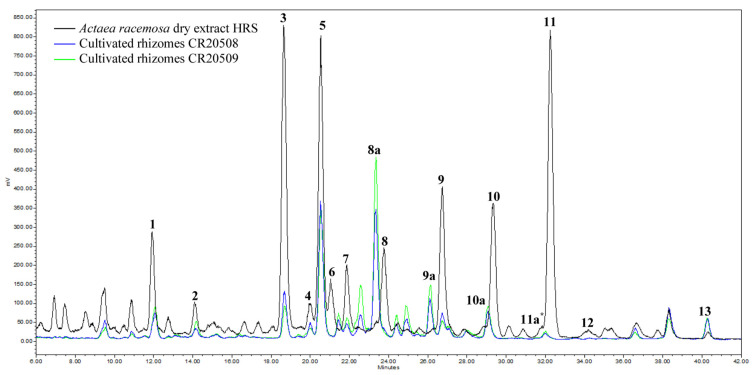
UPLC–ELSD chromatograms of two cultivated rhizomes (CR20508, CR20509) and the *Actaea racemosa* dry extract HRS. * Compound **11a** was detected only in the cultivated rhizomes. A nearby peak in the *Actaea racemosa* dry extract HRS elutes close to **11a**, but MS data confirm that they are different compounds.

**Figure 7:**
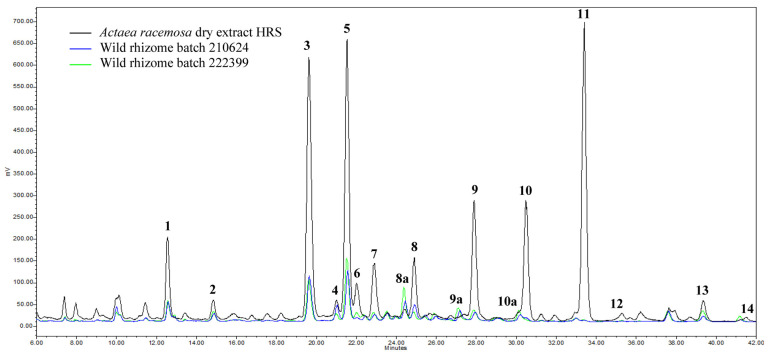
UPLC–ELSD chromatograms of two wild-harvested rhizome batches (210624, 222399) and the *Actaea racemosa* dry extract HRS.

**Figure 8:**
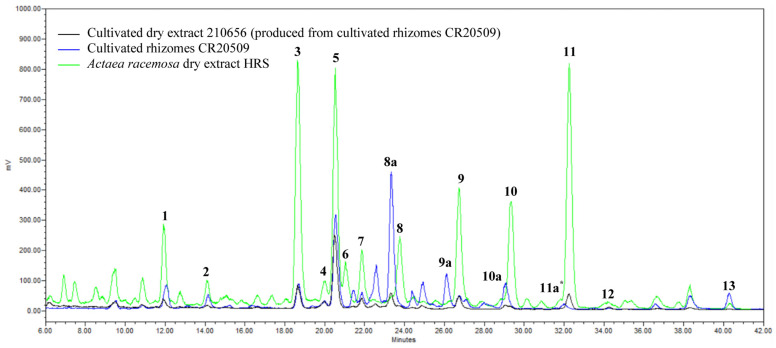
UPLC–ELSD chromatograms of cultivated rhizomes (CR20509), the corresponding dry extract (210656) and the *Actaea racemosa* dry extract HRS. * Compound **11a** was detected only in the cultivated rhizomes.

A major challenge associated with cultivated material was that not all plants exhibited sufficient TTG contents to comply with Ph. Eur. requirements, due to low levels and/or absence of peaks **8**, **9**, **10** and **11**. Peaks **8a**, **9a**, **10a** were present at considerably higher levels in cultivated plants than in wild plants, whereas they occurred at lower levels or were not detected in the corresponding dry extracts (Figure 6, Figure 7 and Figure 8). Furthermore, peak **11a** occurred exclusively in cultivated plants, being absent in the wild ones and all dry extracts.

The chromatographic profile differences between wild and cultivated plants of *A. racemosa*, as well as between cultivated plants and their corresponding dry extracts, can be explained by the existence of TTG originating from compounds **8a**, **9a**, **10a** and possibly the putative compound **11a**. This conversion appears to be enhanced during the production of dry extract. In the two cultivated plants investigated, the total TTG content was 1.56% ± 0.03% for CR20508 and 1.49% ± 0.03% for CR20509, whereas the corresponding dry extracts contained 8.53% ± 0.01% for 210655 and 8.43% ± 0.06% for 210656.

The batch-specific DER values were calculated from production data based on the amount of plant material used and the dry residue of the corresponding spissum extract.

Based on the drug-extract ratios of 3.98 for 210655 and 3.95 for 210656, the expected TTG contents would be 6.20% and 5.89%. When calculated using the Ph. Eur. peak selection, the excess TTG observed in the dry extracts accounted for approximately 27.3% and 30.2% of the measured total TTG content, respectively. However, when compounds **8a**, **9a** and **10a** were additionally included in the calculation, the excess was reduced to 3.5% for extract 210655, while no positive excess was observed for extract 210656 (Table 1). This indicates that inclusion of the malonylated compounds largely explains the increase in TTG content during extraction.

The quantitative differences were accompanied by marked changes in the chromatographic profile. During production of the corresponding dry extracts, peaks 5, 8, 9, 10 and 11 increased, while additional malonylated peaks either decreased or disappeared, consistent with the conversion processes outlined in Scheme 2. The higher presence of compounds **8a**, **9a**, **10a** and putative compound **11a** in cultivated rhizomes can be attributed to the milder post-harvest conditions. Consequently, the conversion process is still at an incipient stage in the cases of cultivated plants.

## 3. Discussion

This study confirmed previously published data on the conversion of TTGs and provided new insights into the behavior of malonylated glycosides. Most of the 12 peaks specified in the Ph. Eur. method could be assigned, and compounds **5b**, **8a**, **9a**, **10a**, **13** and **8b** were isolated and structurally characterized. Together, these findings support a conversion network between malonylated precursors and the corresponding non-malonylated TTGs. Compound **11a** could not be isolated in the amount and purity required for NMR analysis and should therefore be regarded as tentatively assigned. Full characterization of **11a** will require targeted isolation from cultivated plant material enriched in this peak. Future work should use larger amounts of selected starting material, mild extraction conditions and LC–MS-guided preparative purification with narrow collection windows. Rapid processing of collected fractions and, if necessary, repeated semi-preparative purification may allow recovery of sufficient material for NMR-based structural confirmation and sugar analysis.

The relevance of malonylated glycosides as processing-sensitive constituents is supported by observations from other plant systems. In ginseng, malonylated ginsenosides show organ-dependent distribution, with particularly high relative levels reported in fibrous roots [19], and studies on steamed American ginseng (*Panax quinquefolius*) have shown that these compounds can decrease during processing and convert into acetylated and neutral ginsenosides [20]. Similar processing-related conversion has been reported for malonyl isoflavone glycosides in soybean (*Glycine max* L.) and Astragali Radix (*Astragalus membranaceus* (Fisch.) Bge.) [21,22]. These examples support the interpretation that malonylated glycosides can represent processing-sensitive precursor forms whose abundance depends on plant matrix, extraction and handling conditions.

Against this background, the data indicate that the lower content or absence of acetylhydroshengmanol (**8** and **9**) and cimigenol glycosides (**10** and **11**) in the investigated cultivated plants is related to the incomplete transformation of their malonylated precursors and other unidentified malonyl arabinosides. During production of the corresponding dry extracts, this conversion appears to proceed further, as reflected by the increase in peaks 5, 8, 9, 10 and 11. Thus, in the two cultivated plant/dry extract batches investigated, extraction and drying led to a TTG profile more closely aligned with the Ph. Eur. dry extract profile.

This observation is also relevant for the use of cimigenol-3-*O*-α-L-arabinoside, also referred to as cimiracemoside C (**10**), as a marker compound for *A. racemosa*. Although this compound has been proposed as a useful marker for species identification [11,12], the present results show that its diagnostic interpretation requires caution. In the investigated cultivated batches, compound **10** was low or absent in the plant material but increased after extraction, similar to compound **11.** While the xyloside conversion sequence leading to compound **11** is supported by the compounds characterized in this study, the corresponding arabinoside sequence leading to compound **10** could only be partially characterized. Specifically, 24-*O*-acetylhydroshengmanol-3-*O*-α-L-arabinopyranoside was assigned as compound **8**, and peak 4 was tentatively classified as a possible acetylshengmanol arabinoside; however, the corresponding malonylated arabinoside precursors were not identified. Therefore, cimigenol-3-*O*-α-L-arabinoside (**10**) should be interpreted together with the broader TTG profile rather than as a stand-alone exclusion marker for *A. racemosa* identity.

The quantitative relevance of these profile differences is further supported by the recalculation of TTG contents with and without compounds **8a**, **9a** and **10a**. These malonylated compounds had a substantial impact on the apparent TTG balance between cultivated plant material and the corresponding dry extracts. Their inclusion largely reduced or eliminated the excess TTG observed after extraction. These results support further evaluation of whether peaks corresponding to the malonylated compounds **8a**, **9a** and **10a** should be considered in the quantitative assessment of TTGs in *A. racemosa* rhizomes.

While these findings provide important insights, several methodological limitations should be considered when interpreting the results. One limitation is that the comparison between cultivated plant material and corresponding dry extracts was based on two batches from a single cultivation and manufacturing setting, as comparable plant material and corresponding extracts from other manufacturers or extraction processes were not available. A further limitation is that peaks 2, 4, 6, 7 and 12, as well as compound **11a**, could not be unambiguously established, although all were assigned to the triterpene glycoside class. Nevertheless, the proposed conversion pathways are supported by the degradation behavior observed during isolation and sample handling.

Finally, the observed differences between the *A. racemosa* drug HRS and the *A. racemosa* dry extract HRS suggest that future pharmacopeial discussions may benefit from considering an additional plant material-based reference chromatogram.

## 4. Materials and Methods

### 4.1. Materials

All solvents used were of HPLC grade. Acetonitrile, methanol, ethyl acetate, acetic acid, and formic acid were purchased from VWR Chemicals (Radnor, PA, USA) and hexane from Sigma-Aldrich (St. Louis, MO, USA). Water was obtained from an ELGA Ultrapure water system PURELAB^®^ 7120 (ELGA LabWater, High Wycombe, UK). LC-MS-grade solvents purchased from Merck (Darmstadt, Germany) were used for HRMS analysis.

Acetyl chloride was purchased from Sigma-Aldrich (St. Louis, MO, USA); (S)-(+)-2-butanol and trifluoroacetic anhydride (TFAA) were bought from TCI Chemicals (Tokyo, Japan). Reference sugars for sugar analysis were obtained from Sigma-Aldrich (L-arabinose, D-xylose).

### 4.2. Plant Material

Rhizomes of *Cimicifuga racemosa* (L.) Nutt. for the isolation of **5b**, **8a**, **9a**, **10a** and **13** (CR20510) were obtained from VitaPlant (Uttwil, Switzerland), clone CR 81-1 established in Holland in 2016 and collected in April 2020. CR20509 was obtained from the same clone and collection time. CR20508 was obtained from clone CR 68-7, established and collected at the same time. The clone CR 81-1 was also registered with the Swiss Federal Plant Variety Office under the variety name “CIMIZELL”. Genetic testing and confirmation of the two clones CR 81-1 and CR 68-7 as *Cimicifuga racemosa* (L.) Nutt. had already been carried out using High-Resolution Melting Analysis at the University of Vienna. A living specimen is available at Max Zeller Söhne AG, Uttwil, Switzerland.

Dry extracts 210656 and 210655 were produced by Max Zeller Söhne AG from cultivated rhizomes CR20509 and CR20508 respectively. The drug-extract ratio was 3.952 for dry extract 210656 and 3.975 for dry extract 210655.

Compound **8b** was isolated from the dry extract (batch number 210301) of *Cimicifuga racemosa* (L.) Nutt. rhizomes, produced by Max Zeller Söhne AG. The extract was prepared from wild-harvested rhizomes (batch CR210624) collected in Kentucky, USA, between August and November 2020. A voucher specimen (CR-2302) is deposited at Max Zeller Söhne AG, Romanshorn, Switzerland.

An additional wild-harvested rhizome batch (222399) was collected in Kentucky, United States, between September and November 2021.

The 6 individual rhizomes of *Cimicifuga racemosa* (L.) Nutt. (CR23504, CR23505, CR23506, CR23507, CR23508, CR23509) were collected as whole plants by American Botanicals (Secaucus, NJ, USA) and shipped fresh to VitaPlant.

### 4.3. Reference Standards

Commercially available TTGs were employed as reference standards. Cimiracemoside A was purchased from Chromadex (Los Angeles, CA, USA), while cimigenol aglycone was sourced from Chemfaces (Wuhan, China). Actein, 23-epi-26-deoxyactein, 24-*O*-acetylhydroshengmanol-3-*O*-β-D-xylopyranoside, cimigenol-3-*O*-α-L-arabinopyranoside (cimiracemoside C) and cimigenol-3-*O*-β-D-xylopyranoside were purchased from PhytoLab (Vestenbergsgreuth, Germany). The purity of all reference compounds exceeded 90% as determined by HPLC and NMR.

*Actaea racemosa* for assay CRS, *Actaea racemosa* dry extract and drug as HRS were purchased from EDQM (Strasbourg, France).

### 4.4. Analytical HPLC and UPLC Analysis of Dry Extracts and Plant Materials

UPLC analyses were performed using an ACQUITY UPLC H-Class system (Waters, Milford, MA, USA) coupled to an evaporative light-scattering detector (ELSD, 1260 series, Agilent Technologies, Santa Clara, CA, USA), whereas HPLC analyses were carried out on an ACQUITY Arc HPLC system (Waters, USA) coupled to a second ELSD.

Analytical separations were achieved on a Zorbax Eclipse XDB-C18 column (5 μm, 4.6 × 250 mm i.d., Agilent Technologies, USA), and data acquisition was handled by Empower 3 software (Waters, USA).

The analyses followed the method described in the Ph. Eur. monograph for the quantification of TTGs in the rhizomes of *A. racemosa* [2,16]. The mobile phase system consisted of water containing 0.1% formic acid (A) and acetonitrile–methanol, 50:50, *v*/*v*, containing 0.1% formic acid (B). The flow rate was set to 1.0 mL/min, and the gradient was increased from 50% to 80% B over 40 min. ELSD settings included an evaporator temperature of 100 °C, nebulizer temperature of 60 °C, and gas flow rate of 0.9 mL/min using nitrogen as the carrier gas.

Plant material samples were prepared at a concentration of 80 mg/mL in methanol–water, 1:1, *v*/*v*, whereas dry extracts were prepared at 50 mg/mL in methanol. Quantification of total triterpene glycosides was performed according to the Ph. Eur. assay using the official *Actaea racemosa* for assay CRS, containing monoammonium glycyrrhizate, as calibration reference. The *A. racemosa* dry extract HRS was used only as a chromatographic reference for peak assignment and profile comparison. Quantitative analyses were performed in analytical duplicate, and results are reported as means ± SD.

The UPLC–ELSD method was verified for its intended use in accordance with the requirements of Ph. Eur. General Chapter 5.26 (“Verification of Pharmacopoeial Procedures”). The verification included assessments of selectivity/specificity, repeatability and robustness, as well as the stability of reference and test solutions, variation in sample preparation and variation in column batches.

### 4.5. Identification of TTGs Based on Reference Standards and Isolated Compounds

Identification of TTGs was achieved by LC–HRMS on an Acquity UPLC H-Class system coupled to a SYNAPT G2-S Q-TOF mass spectrometer equipped with an electrospray ionization source (ESI). Instrument control and data processing were performed using MassLynx 4.2 software (all Waters, Milford, MA, USA).

UPLC–ESI–QTOF analyses were carried out under the chromatographic conditions described in Section 4.4, based on the Ph. Eur. monograph method [2,16]. Mass spectra were acquired in positive mode over the range *m*/*z* 100–1000. The capillary voltage was set at 3000 V, and the cone voltage was 40 V. Nitrogen was used as the nebulizing and desolvation gas. The desolvation and cone gas flow rates were 600 and 0 L/h, respectively. The desolvation temperature was 250 °C, and the source temperature was 80 °C.

Reference standards and isolated substances were prepared in methanol at concentrations ranging from 250 to 300 ng/mL, while the *A. racemosa* dry extract HRS was dissolved in methanol at a concentration of 750 µg/mL.

### 4.6. Extraction and Isolation of Compounds ***5b*** and ***13***

First, 200 g of powdered plant material was mixed in a blender with 1 L methanol for 5 min at room temperature. The supernatant was filtered and dried under vacuum. The residue was redissolved in 250 mL ethyl acetate, sonicated, allowed to stand overnight, filtered and dried under vacuum.

The resulting methanol–ethyl acetate extract was fractionated by centrifugal partition chromatography (CPC) on an ARMEN SPOT CPC Instrument System (SCPC-100, rotor volume 100 mL, AlphaCrom, Rheinfelden, Switzerland) equipped with a ProStar 210 Solvent Delivery Module and a ProStar 701 X-Y Fraction Collector. The biphasic solvent system consisted of n-hexane–ethyl acetate–methanol–water, 3:7:3:7, *v*/*v*/*v*/*v*. A 2 g portion of the extract was dissolved in a mixture of the upper and the lower phase (5 mL each), and the entire solution (10 mL) was injected. CPC separation was conducted in descending mode at a rotational speed of 2000 rpm and a flow rate of 5 mL/min. Fractions were collected every 2 min over 80 min, resulting in 40 fractions, which were subsequently combined and dried according to their UPLC profile.

The fraction F31–F33 (t_R_ 60–66 min) was further purified by semi-preparative reverse-phase high-performance liquid chromatography (RP–HPLC) using an UPLC Acquity H-Class system coupled to a QDa detector with ESI-source (Waters, USA). The QDa detector was operated in positive ESI full-scan mode over the range *m*/*z* 350–1250. The capillary voltage was set to 1.2 kV, the cone voltage to 20 V, and the probe temperature to 450 °C. The separation was performed on a Reprospher 100 C18-DE (5 µm, 10 × 150 mm i.d., Dr. Maisch, Ammerbuch-Entringen, Germany) column coupled with a Reprospher 100 C18-DE (5 µm, 10 × 30 mm i.d. Dr. Maisch, Germany) pre-column. Water (A) and acetonitrile–methanol, 50:50, *v*/*v* (B), were used as mobile phases. The gradient program was as follows: 1–10 min, isocratic with 50% B; 10–80 min, 50–90% B, at a flow rate of 2.2 mL/min. For MS-detection, the flow was split at a ratio of 1:100, and a make-up flow of water–acetonitrile–methanol, 50:25:25, *v*/*v*/*v*, containing 0.2% formic acid was added at 0.022 mL/min using the Isocratic Solvent Manager (Waters, USA).

Target compounds were collected through time-based windows using a Waters Fraction Manager-Analytical (WFM-A, Waters, Milford, MA, USA) and lyophilized using a Lyoph Alpha 3–4 LSC basic freeze dryer (Christ, Osterode am Harz, Germany), yielding 5.9 mg of **5b** (t_R_ = 45.9 min) and 6.0 mg of **13** (t_R_ = 71.8 min), respectively.

### 4.7. Extraction and Isolation of Compounds ***8a***, ***9a*** and ***10a***

First, 500 g of powdered plant material was extracted with 5 L of ethyl acetate for 8 h at room temperature. The extract was filtered, and the remaining plant material residue was re-extracted with 1 L methanol for 3 h at room temperature, filtered and dried.

Then, 20 mg/mL methanolic extract samples were subjected to semi-preparative RP–HPLC on a Zorbax Eclipse XDB-C18 (5 µm, 9.4 × 250 mm i.d., Agilent Technologies, USA) column. The separation was performed on the same UPLC Acquity H-Class system coupled to a QDa detector as described in Section 4.6, using the same QDa detector settings. The chromatographic conditions were modified as follows: eluent A consisted of water acidified with 0.05% acetic acid, while eluent B comprised acetonitrile–methanol, 1:1, *v*/*v*, likewise acidified with 0.05% acetic acid. The gradient program was as follows: 1–50 min, 60–65% B; 50–65 min, 65–67.5% B; flow rate of 2 mL/min. For the MS-detection, the flow was split at a ratio of 1:10, while adding 0.2 mL/min of water–acetonitrile–methanol, 50:25:25, *v*/*v*/*v*, containing 0.15% acetic acid with the Isocratic Solvent Manager. Collection of the compounds was carried out over several days, and after each collection day the compounds were lyophilized. Before combining the dried compounds obtained after each collection day, the purity was tested using MS. In this way, 2.2 mg of **8a** (t_R_ = 30.9 min), 3.3 mg of **9a** (t_R_ = 34.1 min) and 1.7 mg of **10a** (t_R_ = 48.1 min) were obtained.

### 4.8. Extraction and Isolation of Compound ***8*** for Obtaining ***8b***

The dry extract (batch number 210301) was produced by two successive extractions of the rhizomes with 60% ethanol (*v*/*v*) for 3 h first and then for 2 h at room temperature. After filtration, Povidon 30 (extract–excipient ratio: 3:1, JRS Pharma, Rosenberg, Germany) was added to the liquid extract, which was then partially concentrated and spray-dried. Then, 100 g of dry extract was extracted with 5 L of ethyl acetate for one hour at room temperature. The extract was filtered and dried.

Extract samples were subjected to semi-preparative RP-HPLC using the same column, sample concentration, UPLC Acquity H-Class system, and QDa detection settings as described in Section 4.7. Chromatographic separation was carried out with a non-acidified binary solvent system composed of water (A) and acetonitrile–methanol, 1:1, *v*/*v* (B). The gradient conditions were as follows: 1–30 min, 62–65% B; 30–45 min, 65–67.5% B, flow rate of 2 mL/min. For the MS-detection, the flow was split 1:100, while adding 0.2 mL/min of water–acetonitrile–methanol, 50:25:25, *v*/*v*/*v,* containing 0.15% formic acid with the Isocratic Solvent Manager. Compound **8** was collected through time-based windows and dried, yielding 3.1 mg of **8** (t_R_ = 31.5 min). Given the low purity of compound **8** and the small amount for a proper NMR identification, it was further subjected to forced degradation, in which compound **8** was dissolved in methanol and subjected to heating for **8** h at 95 °C, resulting in compound **8b**.

### 4.9. Characterization Data of Compounds ***5b***, ***8a***, ***9a***, ***10a***, ***13*** and ***8b***

23-*O*-Acetylshengmanol-3-*O*-β-D-xylopyranoside (**5b**): white amorphous powder; [α]_D_^25^ −53.8° (c 0.2, methanol); ^1^H and ^13^C NMR, see Table S1, Supporting Information; HRESIMS *m*/*z* 685.3928 [M+Na]^+^ (calcd for C_37_H_58_NaO_10_^+^, 685.3922).

23-*O*-Acetylshengmanol-3-*O*-(2′-*O*-malonyl)-β-D-xylopyranoside (**8a**): white amorphous powder; [α]_D_^25^ −17.9° (c 0.07, methanol); ^1^H and ^13^C NMR, see Table S1, Supporting Information; HRESIMS *m*/*z* 771.3923 [M+Na]^+^ (calcd for C_40_H_60_NaO_13_^+^, 771.3926).

23-*O*-Acetylshengmanol-3-*O*-(2′,4′-*O*-dimalonyl)-β-D-xylopyranoside (**9a**): white amorphous powder; [α]_D_^25^ −28.6° (c 0.2, methanol); ^1^H and ^13^C NMR, see Table S1, Supporting Information; HRESIMS *m*/*z* 857.3761 [M+Na]^+^ (calcd for C_43_H_62_NaO_16_^+^, 857.3930).

24-*O*-Acetylhydroshengmanol-3-*O*-(2′-*O*-malonyl)-β-D-xylopyranoside (**10a**): white amorphous powder; [α]_D_^25^ −8.2 (c 0.1, methanol); ^1^H and ^13^C NMR, see Table S1, Supporting Information; HRESIMS *m*/*z* 789.4022 [M+Na]^+^ (calcd for C_40_H_62_NaO_14_^+^, 789.4032).

Shengmanol-3-*O*-β-D-xylopyranoside (**13**): white amorphous powder; [α]_D_^25^ −5.5 (c 0.16, methanol); ^1^H and ^13^C NMR, see Table S1, Supporting Information; HRESIMS *m*/*z* 643.3823 [M+Na]^+^ (calcd for C_35_H_56_NaO_9_^+^, 643.3817).

24-*O*-hydroshengmanol-3-*O*-α-L-arabinopyranoside (**8b**): white amorphous powder; [α]_D_^25^ +11.0° (c 0.15, methanol); ^1^H and ^13^C NMR, see Table S1, Supporting Information; HRESIMS *m*/*z* 661.3865 [M+Na]^+^ (calcd for C_35_H_58_NaO_10_^+^, 661.3928).

### 4.10. NMR Spectroscopy

NMR spectra were recorded on a Bruker Avance III spectrometer (Fällanden, Switzerland) operating at 500 MHz for ^1^H and 126 MHz for ^13^C at 23 °C with a 5 mm BBO probe. Spectra were measured in DMSO-*d*_6_ (purity > 99.5%; Sigma-Aldrich, St. Louis, MO, USA). Data were analyzed using Spectrus Processor (ACD/Lab, Toronto, ON, Canada) software (version ACD/Labs 2023.1.0).

### 4.11. GC–MS Identification of the Sugar Moiety

GC–MS was performed on a Hewlett-Packard GC/MS system (Agilent G1503A-6890 Plus GC) equipped with a 5973 Mass Selective Detector (MSD) and a 59864B Ionization Gauge Controller (Agilent Technologies). A J&W DM-225 GC column (30 m; i.d.: 0.25 mm; film thickness: 0.25 µm; Agilent Technologies) was used. Injector temperature was 280 °C. Helium (0.7 mL/min) was used as a carrier gas. Transfer line temperature was 240 °C. The following temperature program was applied: 60 °C hold for 1 min, increase to 240 °C at 10 °C/min followed by 5 min at 240 °C. EI ionization was performed at a temperature of 240 °C with an electron energy of 70 eV. Mass scans from *m*/*z* 50–700 were recorded. Data acquisition was performed by MSD ChemStation G1701DA D.03.00.611 software (Agilent Technologies) and data were processed with Spectrus Processor (ACD-Lab, Toronto, ON, Canada).

Each compound (1.3 mg for **5b**, 0.4 mg for **8a**, 1.4 mg for **9a**, 0.65 mg for **10a**, 1 mg for **13** and 1.9 mg for **8b**) was hydrolyzed with 2 N HCl (0.5 mL) for 15 h at 100 °C. The hydrolysate was extracted twice with ethyl acetate (2 × 0.5 mL). The aqueous phase was dried under a flow of nitrogen (1 h at 35 °C) and then under high vacuum (5 h, lyophilizer). The residue was treated with 250 µL of a mixture of (+)-2-butanol–acetyl chloride, 10:0.5, *v*/*v*, at 55 °C for 15 h. After drying under a stream of nitrogen, the residue was derivatized with 250 µL of a mixture of trifluoroacetic anhydride–ethyl acetate, 3:4, *v*/*v*, at 55 °C for 1 h. The samples were diluted with 750 µL of HPLC-grade ethyl acetate. Then, 1 μL of each derivatized sample was injected using an HTS-PAL 113542 autosampler (CTC-Analytics, Zwingen, Switzerland) into the GC–MS in split mode (split ratio 1:50). L-arabinose (0.9 mg), D-arabinose (0.6 mg), L-xylose (0.9 mg) and D-xylose (1.6 mg) derivatized in the same manner were used as references.

### 4.12. Polarimetric Measurements

Optical rotations were measured on a JASCO P-2000 polarimeter (Portmann Instruments AG, Biel-Benken, Switzerland) equipped with a 1 dm path-length sample cell at a constant temperature of 25 °C and a wavelength of 589 nm (Na-D line).

## Figures and Tables

**Table 1:** Comparison of TTG contents calculated with the current Ph. Eur. peak selection and with additional consideration of malonylated TTGs **8a**, **9a** and **10a** in cultivated plants and corresponding dry extracts.

Plant Material	Dry Extract	Calculation	TTG in Plant Material (%)	DER	Expected TTG in Extract (%)	Measured TTG in Extract (%)	Excess
CR20508	210655	Ph. Eur. peak selection	1.56 ± 0.03	3.98	6.20	8.53 ± 0.01	27.31%
CR20508	210655	Considering **8a**, **9a**, **10a**	2.49 ± 0.01	3.98	9.90	10.26 ± 0.07	3.50%
CR20509	210656	Ph. Eur. peak selection	1.49 ± 0.03	3.95	5.89	8.43 ± 0.06	30.26%
CR20509	210656	Considering **8a**, **9a**, **10a**	2.61 ± 0.04	3.95	10.31	9.87 ± 0.01	−4.44%

## Data Availability

The data presented in this study are available in the article and the Supplementary Materials.

## Supplementary Materials

The following supporting information can be downloaded at https://www.mdpi.com/article/10.3390/plants15152272/s1: Table S1: ^1^H and ^13^C NMR spectroscopic data of compounds **5b**, **8a**, **9a**, **10a**, **13** and **8b**; Figures S1–S5: ^1^H-NMR, ^1^H-^1^H COSY, ^1^H-^1^H ROESY, HSQC-DEPT, and HMBC spectra of compound **5b**; Figures S6–S11: ^1^H-NMR, ^13^C-DEPTq, ^1^H-^1^H COSY, ^1^H-^1^H ROESY, HSQC-DEPT, and HMBC spectra of compound **8a**; Figures S12–S17: ^1^H-NMR, ^13^C-DEPTq, ^1^H-^1^H COSY, ^1^H-^1^H ROESY, HSQC-DEPT, and HMBC spectra of compound **9a**; Figures S18–S22: ^1^H-NMR, ^1^H-^1^H COSY, ^1^H-^1^H ROESY, HSQC-DEPT, and HMBC spectra of compound **10a**; Figures S23–S27: ^1^H-NMR, ^1^H-^1^H COSY, ^1^H-^1^H ROESY, HSQC-DEPT, and HMBC spectra of compound **13**; Figures S28–S33: ^1^H-NMR, ^13^C-DEPTq, ^1^H-^1^H COSY, ^1^H-^1^H ROESY, HSQC-DEPT, and HMBC of compound **8b**; Figures S34–S39: HRESIMS spectra of compounds **5b**, **8a**, **9a**, **10a**, **13**, and **8b;** Figures S40–S44: TIC chromatograms of degraded compounds **8a**, **9a**, and **10a**; Figure S45: Key HMBC and ROESY correlations of **5b**, **8a**, **9a**, **10a**, **13** and **8b**.

## Appendix A

**Table A1:** Overview of chromatographic TTG peaks and additional constituents identified or tentatively assigned in *A. racemosa* rhizomes and extracts.

Label/Peak *	t_R_ (min)	Proposed Identity	Molecular Formula	Marker Ion Exact Mass, *m*/*z*	Other Fragments, *m*/*z*	Identification Confidence	Source of Evidence
**1** (Peak 1)	13.05	cimiracemoside A	C_37_H_56_O_11_	699.3671 [M+Na]^+^	659.3754 599.3578 467.3211	Confirmed	Reference standard; RT/HRESIMS match
Peak 2	15.43	deoxyactein-related cycloartane-type TTG	not assigned	661.3895 [M+H-H_2_O]^+^	601.3679 469.3381 451.3256	Tentative class assignment	HRESIMS; analogy to **3b**
**3a** (Peak 3)	20.40	actein	C_37_H_56_O_11_	599.3558 [M+H-H_2_O-acetic acid]^+^	659.3724 467.3202 449.3105	Confirmed	Reference standard; RT/HRESIMS match
**3b** (Peak 3)	20.40	23-*epi*-26-deoxyactein	C_37_H_56_O_10_	683.3707 [M+Na]^+^	661.3895 601.3751 469.3363 451.3264	Confirmed	Reference standard; RT/HRESIMS match
Peak 4	21.81	23-*O*-acetylshengmanol-3-*O*-α-L-arabinopyranoside	C_37_H_58_O_10_	685.3860 [M+Na]^+^	645.3951 513.3596 453.3418	Tentative assignment	HRESIMS; analogy to **5b**
**5a** (Peak 5)	22.36	actein	C_37_H_56_O_11_	599.3553 [M+H-H_2_O-acetic acid]^+^	659.3721 467.3195 449.3109	Confirmed	Reference standard; RT/HRESIMS match
**5b** (Peak 5)	22.36	23-*O*-acetylshengmanol-3-*O*-β-D-xylopyranoside	C_37_H_58_O_10_	685.3928 [M+Na]^+^	645.3931 513.3586 453.3418	Confirmed	Isolated; NMR; HRESIMS; GC–MS sugar analysis
Peak 6	22.85	deoxyactein-related cycloartane-type TTG	not assigned	701.3791 [M+Na]^+^	661.3867 469.3347 451.3260	Tentative class assignment	HRESIMS; analogy to **3b**
Peak 7	23.61	unresolved TTG mixture, molecular masses ~678/680 Da	not assigned	601.3727	663.4432 469.3344 451.3245	unresolved mixture	HRESIMS only
**8a**	25.42	23-*O*-Acetylshengmanol-3-*O*-(2′-*O*-malonyl)-β-D-xylopyranoside	C_40_H_60_O_13_	771.3923 [M+Na]^+^	667.3801 435.3254	Confirmed	Isolated; NMR; HRESIMS; GC–MS sugar analysis
**8** (Peak 8)	25.69	24-*O*-acetylhydroshengmanol-3-*O*-α-L-arabinopyranoside	C_37_H_60_O_11_	703.3958 [M+Na]^+^	645.3958 585.3748 513.3595	Confirmed	HRESIMS; HRESIMS similarity to **9**; degradation product **8b** confirmed by NMR/GC–MS sugar analysis
**8b**	27.32	24-*O*-hydroshengmanol-3-*O*-α-L-arabinopyranoside	C_35_H_58_O_10_	661.3865 [M+Na]^+^	677.3594 643.3767 435.3318	Confirmed	Isolated; NMR; HRESIMS; GC–MS sugar analysis
**9a**	28.28	23-*O*-Acetylshengmanol-3-*O*-(2′,4′-*O*-dimalonyl)-β-D-xylopyranoside	C_43_H_62_O_16_	857.3761 [M+Na]^+^	873.3387 535.3391	Confirmed	Isolated; NMR; HRESIMS; GC–MS sugar analysis
**9** (Peak 9)	28.77	24-*O*-acetylhydroshengmanol-3-*O*-β-D-xylopyranoside	C_37_H_60_O_11_	703.3945 [M+Na]^+^	645.3942 585.3756 513.3590	Confirmed	Reference standard; RT/HRESIMS match
**10a**	31.20	24-*O*-Acetylhydroshengmanol-3-*O*-(2′-*O*-malonyl)-β-D-xylopyranoside	C_40_H_62_O_14_	789.4022 [M+Na]^+^	771.3923 731.4003	Confirmed	Isolated; NMR; HRESIMS; GC–MS sugar analysis
**10** (Peak 10)	31.56	cimigenol-3-*O*-α-L-arabinoside	C_35_H_56_O_9_	643.3763 [M+Na]^+^	585.3762 453.3409 435.3311	Confirmed	Reference standard; RT/HRESIMS match
**11a**	34.22	putative dimalonylated acetylhydroshengmanol-type	not assigned	875.4048 [M+Na]^+^	817.4026 789.4046 731.4029	Tentative class assignment	HRESIMS; analogy to **10a**
**11** (Peak 11)	34.60	cimigenol-3-*O*-β-D-xyloside	C_35_H_56_O_9_	643.3765 [M+Na]^+^	585.3764 453.3419 435.3319	Confirmed	Reference standard; RT/HRESIMS match
Peak 12	35.56	putative malonylated cimigenol-/shengmanol-type	not assigned	729.3720 [M+Na]^+^	643.3783 585.3724 435.3296	Tentative class assignment	HRESIMS; analogy to **10**, **11** and **13**
**13**	40.21	Shengmanol-3-*O*-β-D-xylopyranoside	C_35_H_56_O_9_	643.3823 [M+Na]^+^	659.3560 585.3790 435.3261	Confirmed	Isolated; NMR; HRESIMS; GC–MS sugar analysis
**14**	42.26	cimigenol aglycone	C_30_H_48_O_5_	511.3434 [M+Na]^+^	527.3167	Confirmed	Reference standard; RT/HRESIMS match

* Numbers 1–12 without letters refer to chromatographic peaks defined by the *A. racemosa* dry extract HRS chromatogram used in the Ph. Eur. assay. Additional labels, including **3a**/**3b**, **5a**/**5b**, **8a**, **8b**, **9a**, **10a**, **11a**, **13** and **14**, refer to individual constituents that either co-elute within one of these peaks or represent structurally related compounds detected outside the 12 main HRS peaks.
