# Supplementary material for: Bridging Pharmacopeial Standards and Chemical Reality of Triterpene Glycosides in Actaea racemosa
Source: Plants (Basel). 2026 Jul 24;15(15):2272. doi: 10.3390/plants15152272 (PMC13468391; doi:10.3390/plants15152272)
Supplement: Supplementary file 1 [file plants-15-02272-s001.zip › plants-4413706-supplementary.pdf]

plants

Supplementary Materials

Bridging Pharmacopoeial Standards and Chemical Reality of Triterpene Glycosides in *Actaea racemosa*

Larisa-Mălina Cătăuță [1], Roman Senn [1], Alexander Schenk [1], Amin Chaanin [2], Christiane Halbsguth [1,*] and Ombeline Danton [1]

[1] Max Zeller Söhne AG, Seeblickstrasse 4, 8590 Romanshorn, Switzerland; larisa.catauta@zellerag.ch (L.-M.C.); roman.senn@zellerag.ch (R.S.); aschenk58@icloud.com (A.S.); ombelined@gmail.com (O.D.)
[2] VitaPlant AG, Romanshornerstrasse 39, 8592 Uttwil, Switzerland; achaanin58@icloud.com
* Correspondence: christiane.halbsguth@zellerag.ch; Tel.: +41-71-466-0772

List of Supplementary Materials

Table S1. ^{1}H and ^{13}C NMR Spectroscopic Data of Compounds 5b, 8a, 9a, 10a, 13 and 8b (DMSO-d_6; 500 MHz for ^{1}H and 126 MHz for ^{13}C NMR; δ in ppm)........ 1

Figure S1. ^{1}H-NMR spectrum of compound 5b (500 MHz, DMSO-d_6). 3

Figure S2. ^{1}H-^{1}H COSY spectrum of compound 5b (500 MHz, DMSO-d_6). 4

Figure S3. ^{1}H-^{1}H ROESY spectrum of compound 5b (500 MHz, DMSO-d_6). 5

Figure S4. HSQC-DEPT spectrum of compound 5b (500 MHz, DMSO-d_6)........ 6

Figure S5. HMBC spectrum of compound 5b (500 MHz, DMSO-d_6)........ 7

Figure S6. ^{1}H-NMR spectrum of compound 8a (500 MHz, DMSO-d_6). 8

Figure S7. ^{13}C-DEPTq spectrum of compound 8a (500 MHz, DMSO-d_6). 9

Figure S8. ^{1}H-^{1}H COSY spectrum of compound 8a (500 MHz, DMSO-d_6)........ 10

Figure S9. ^{1}H-^{1}H ROESY spectrum of compound 8a (500 MHz, DMSO-d_6). 11

Figure S10. HSQC-DEPT spectrum of compound 8a (500 MHz, DMSO-d_6). 12

Figure S11. HMBC spectrum of compound 8a (500 MHz, DMSO-d_6). 13

Figure S12. ^{1}H-NMR spectrum of compound 9a (500 MHz, DMSO-d_6)........ 14

Figure S13. ^{13}C-DEPTq spectrum of compound 9a (500 MHz, DMSO-d_6)........ 15

Figure S14. ^{1}H-^{1}H COSY spectrum of compound 9a (500 MHz, DMSO-d_6)........ 16

Figure S15. ^{1}H-^{1}H ROESY spectrum of compound 9a (500 MHz, DMSO-d_6). 17

Figure S16. HSQC-DEPT spectrum of compound 9a (500 MHz, DMSO-d_6). 18

Figure S17. HMBC spectrum of compound 9a (500 MHz, DMSO-d_6). 19

Figure S18. ^{1}H-NMR spectrum of compound 10a (500 MHz, DMSO-d_6). 20

Figure S19. ^{1}H-^{1}H COSY spectrum of compound 10a (500 MHz, DMSO-d_6)........ 21

Figure S20. ^{1}H-^{1}H ROESY spectrum of compound 10a (500 MHz, DMSO-d_6). 22

Figure S21. HSQC-DEPT spectrum of compound 10a (500 MHz, DMSO-d_6). 23

Figure S22. HMBC spectrum of compound 10a (500 MHz, DMSO-d_6). 24

Figure S23. ^{1}H-NMR spectrum of compound 13 (500 MHz, DMSO-d_6). 25
Figure S24. ^{1}H-^{1}H COSY spectrum of compound 13 (500 MHz, DMSO-d_6). 26
Figure S25. ^{1}H-^{1}H ROESY spectrum of compound 13 (500 MHz, DMSO-d_6). 27
Figure S26. HSQC-DEPT spectrum of compound 13 (500 MHz, DMSO-d_6). 28
Figure S27. HMBC spectrum of compound 13 (500 MHz, DMSO-d_6). 29
Figure S28. ^{1}H-NMR spectrum of compound 8b (500 MHz, DMSO-d6). 30
Figure S29. ^{13}C-DEPTq spectrum of compound 8b (500 MHz, DMSO-d_6). 31
Figure S30. ^{1}H-^{1}H COSY spectrum of compound 8b (500 MHz, DMSO-d_6). 32
Figure S31. ^{1}H-^{1}H ROESY spectrum of compound 8b (500 MHz, DMSO-d_6). 33
Figure S32. HSQC-DEPT spectrum of compound 8b (500 MHz, DMSO-d_6). 34
Figure S33. HMBC spectrum of compound 8b (500 MHz, DMSO-d_6). 35
Figure S34. HRESIMS spectrum of compound 5b. 36
Figure S35. HRESIMS spectrum of compound 8a. 37
Figure S36. HRESIMS spectrum of compound 9a. 38
Figure S37. HRESIMS spectrum of compound 10a. 39
Figure S38. HRESIMS spectrum of compound 13. 40
Figure S39. HRESIMS spectrum of compound 8b. 41
Figure S40. TIC (+) Chromatogram of compound 8a with the degradation products 5b, 9 and 10a. 42
Figure S41. TIC (+) Chromatogram of compound 8a containing its malonyl positional isomers (RT 26.069 min and RT 26.544) and also compound 9a. 43
Figure S42. TIC (+) Chromatogram of compound 9a with the degradation products 8a and 10a. 44
Figure S43. TIC (+) Chromatogram of compound 9a containing its malonyl positional isomers (2′,3′- and 3′,4′-) as well as compound 8a. 45
Figure S44. TIC (+) Chromatogram of compound 10a with the degradation products 9 and 11. 46

Table S1. ^{1}H and ^{13}C NMR Spectroscopic Data of Compounds **5b**, **8a**, **9a**, **10a**, **13** and **8b** (DMSO-d$_6$; 500 MHz for ^{1}H and 126 MHz for ^{13}C NMR; δ in ppm)

	**5b**		**8a**		**9a**		**10a**		**13**		**8b**	
position	δ_C, type[b]	δ_H (*J* in Hz)	δ_C, type	δ_H (*J* in Hz)	δ_C, type	δ_H (*J* in Hz)	δ_C, type[b]	δ_H (*J* in Hz)	δ_C, type	δ_H (*J* in Hz)	δ_C, type	δ_H (*J* in Hz)
1	31.9, CH_2	1.26[a]<br>1.56[a]	31.2, CH_2	1.18[a]<br>1.45[a]	31.2, CH_2	1.20[a]<br>1.47[a]	[c]	[c]	32.0, CH_2	1.27[a]<br>1.53[a]	31.9, CH_2	1.41[a]<br>1.17[a]
2	29.6, CH_2	1.62[a]<br>1.89, m	28.9, CH_2	1.51[a]<br>1.80, m	28.9, CH_2	1.54[a]<br>1.81, m	[c]	[c]	29.7, CH_2	1.61[a]<br>1.90, m	29.6, CH_2	1.52[a]<br>1.82, m
3	87.8, CH	3.18[a]	87.4, CH	3.11[a]	87.9, CH	3.13, m	87.8, CH	3.08[a]	87.8, CH	3.18[a]	87.7, CH	3.07, m
4	41.0, C		40.1, C		40.3, C		[c]		40.9, C		41.0, C	
5	47.3, CH	1.35[a]	46.6, CH	1.26[a]	46.5, CH	1.26[a]	46.5, CH	1.23, m	47.3, CH	1.33, dd (12.2, 4.0)	47.2, CH	1.20[a]
6	20.7, CH_2	0.84[a]<br>1.59[a]	20.2, CH_2	0.72, m<br>1.49[a]	20.2, CH_2	0.73, m<br>1.49[a]	[c]	[c]	20.8, CH_2	0.81, m<br>1.59[a]	20.8, CH_2	0.70[a]<br>1.48[a]
7	25.8, CH_2	1.27[a]<br>2.12[a]	25.2, CH_2	1.16[a]<br>2.02[a]	25.2, CH_2	1.18[a]<br>2.04[a]	[c]	[c]	26.0, CH_2	1.03[a]<br>1.75[a]	26.1, CH_2	1.11[a]<br>1.98[a]
8	47.8, CH	1.79[a]	47.3, CH	1.68[a]	47.4, CH	1.68[a]	[c]	[c]	48.6, CH	1.65[a]	48.5 CH	1.51[a]
9	20.0, C		19.5, C		19.5, C		[c]		20.0, C		19.7, C	
10	26.7, C		26.0, C		26.0, C		[c]		26.5, C		26.4, C	
11	26.2, CH_2	1.08[a]<br>1.74[a]	25.7, CH_2	0.96[a]<br>1.65[a]	25.7, CH_2	0.96[a]<br>1.65[a]	[c]	[c]	26.2, CH_2	1.20[a]<br>2.07, m	26.0, CH_2	1.64[a]
12	32.7, CH_2	1.77[a]	32.0, CH_2	1.69[a]	32.0, CH_2	1.68[a]	[c]	[c]	33.7, CH_2	1.57[a]<br>1.71[a]	33.2, CH_2	1.45[a]
13	41.2, C		40.6, C		40.6, C		33.3, C		41.7, C		41.8, C	
14	45.5, C		44.9, C		44.9, C		41.1, C		46.0, C		46.0, C	
15	82.0, CH	3.88, s	81.4, CH	3.80, d (4.0)	81.4, CH	3.80[a]	81.6, CH	3.78[a]	81.8, CH	3.63, d (4.9)	81.9, CH	3.52, s
16	219.1, C		218.9, C		218.9, C		[c]		102.3, C		100.9, C	
17	59.4, CH	2.12[a]	58.8, CH	2.06, d (5.8)	58.8, CH	2.06[a]	[c]	[c]	60.4, CH	1.56[a]	60.6, CH	1.43[a]
18	19.8, CH_3	1.25[a]	19.4, CH_3	1.16, s	19.4, CH_3	1.16, s	19.8, CH_3	1.01, s	20.3, CH_3	1.13, s	21.9, CH_3	0.90[a]
19	30.3, CH_2	0.45, br s<br>0.65, br s	29.9, CH_2	0.38, d (3.7)<br>0.55, d (3.4)	30.0, CH_2	0.38, br s<br>0.55, br s	31.3, CH_2	0.30, br s<br>0.51, br s	30.7, CH_2	0.39, d (3.7)<br>0.64, d (3.7)	30.5, CH_2	0.32, br s<br>0.54, br s
20	27.4, CH	1.78[a]	26.7, CH	1.65[a]	26.7, CH	1.66[a]	26.7, CH	1.53, m	26.4, CH	1.69[a]	26.9, CH	[c]
21	20.3, CH_3	1.06[a]	19.7, CH_3	0.98, d (6.7)	19.7, CH_3	0.98, d (6.4)	21.2, CH_3	0.89[a]	21.7, CH_3	1.01 d (6.1)	20,4, CH_3	1.06[a]
22	36.4, CH_2	1.52[a]<br>2.22, dd (11.4, 11.4)	35.8, CH_2	1.41[a]<br>2.13, m	35.8, CH_2	1.40[a]<br>2.13, dd<br>(11.9, 11.9)	[c]	[c]	31.6, CH_2	1.57[a]<br>1.65[a]	33.6, CH_2	1.61[a]
23	71.8, CH	4.89, m	71.2, CH	4.83, m	71.2, CH	4.81, t (9.3)	81.1, CH	3.42[a]	75.0, CH	3.55, m	74.7, CH	4.09[a]
24	64.5, CH	2.87, d (7.3)	63.9, CH	2.82, d (8.5)	63.9, CH	2.82, d (8.5)	81.3, CH	4.83, d (8.5)	67.0, CH	3.09[a]	78.1, CH	2.95, d (2.8)

25	58.6, C		58.1, C		58.1, C		70.0, C		57.1, C		72.1, C	
26	19.5, CH_3	1.35, s	24.4, CH_3	1.24, s	24.4, CH_3	1.24, s	24.3, CH_3	1.04, s	19.4, CH_3	1.28[a]	25.4, CH_3	1.04[a]
27	24.9 CH_3	1.32, s	19.0, CH_3	1.27, s	19.0, CH_3	1.27, s	28.5, CH_3	1.04, s	25.5, CH_3	1.28[a]	29.0, CH_3	1.10, s
28	11.7 CH_3	0.90, s	11.3, CH_3	0.81, s	11.3, CH_3	0.81, s	11.3, CH_3	0.77, s	11.7, CH_3	0.90, s	11.6, CH_3	0.78, s
29	15.4 CH_3	0.87, s	24.8, CH_3	0.85, s	24.7, CH_3	0.84, s	24.7, CH_3	0.83, s	15.4, CH_3	0.87, s	25.6, CH_3	0.95[a]
30	25.6 CH_3	1.05[a]	14.6, CH_3	0.67, s	14.8, CH_3	0.68, s	14.8, CH_3	0.65, s	25.6, CH_3	1.05, s	15.4, CH_3	0.79, s
Ac1	170.3, C		170.0, C		170.0, C		170.6, C					
Ac2	21.3 CH_3	2.09[a]	20.8, CH_3	2.03, s	20.8, CH_3	2.03, s	21.1, CH_3	2.00, s				
15-OH				5.26, d (5.2)						5.51 d (5.5)		
16-OH										4.85, s		
2'-OH										4.81, br s		
**Sugar moiety at 3**												
1'	106.4, CH	4.22, d (6.1)	102.6, CH	4.37, d (7.9)	102.3, CH	4.52[a]	102.6, CH	4.41[a]	106.3, CH	4.23, d (7.6)	106.2, CH	4.13[a]
2'	74.4, CH	3.06[a]	75.1, CH	4.52, dd (7.9, 9.2)	74.8, CH	4.54[a]	75.1, CH	4.41[a]	74.4, CH	3.07[a]	71.5, CH	3.33[a]
3'	77.3, CH	3.18[a]	74.2, CH	3.29, dd (9.5, 9.5)	72.1, CH	3.67, t (7.9)	75.5, CH	3.34[a]	77.3, CH	3.19[a]	73.15, CH	3.31[a]
4'	70.2, CH	3.34, m	69.6, CH	3.35[a]	70.4, CH	4.58, m	68.7, CH	3.34[a]	70.2, CH	3.36[a]	68.1, CH	3.60, br s
5'a	66.1, CH_2	3.73, m	65.6, CH_2	3.70, dd (11.3, 5.2)	62.1, CH_2	3.81[a]	65.9, CH_2	3.68, m	66.1, CH_2	3.74, dd (11.3, 5.2)	65.5, CH_2	3.36[a]
5'b		3.10[a]		3.12[a]		3.26[a]		3.10[a]		3.11[a]	65.5, CH_2	3.63, dd (12.1, 2.9)
**Malonyl group**												
Mal2'-1			166.1, C		168.8, C		167.9, C					
Mal2'-2			41.8, CH_2	3.24, d (15.6)<br>3.36[a]	45.3, CH_2	2.87, d (14.3)<br>3.03, d (14.0)	45.4, CH_2	2.84, d (14.3)<br>3.00, d (14.3)				
Mal2'-3			167.5[b]		168.0, C		169.0, C					
Mal4'-1					169.2, C							
Mal4'-2					45.6, CH_2	2.94, s						
Mal4'-3					168.1, C							

[a] Overlapping signals, [b] Chemical shifts obtained from the HSQC and HMBC, [c] Signal not visible.

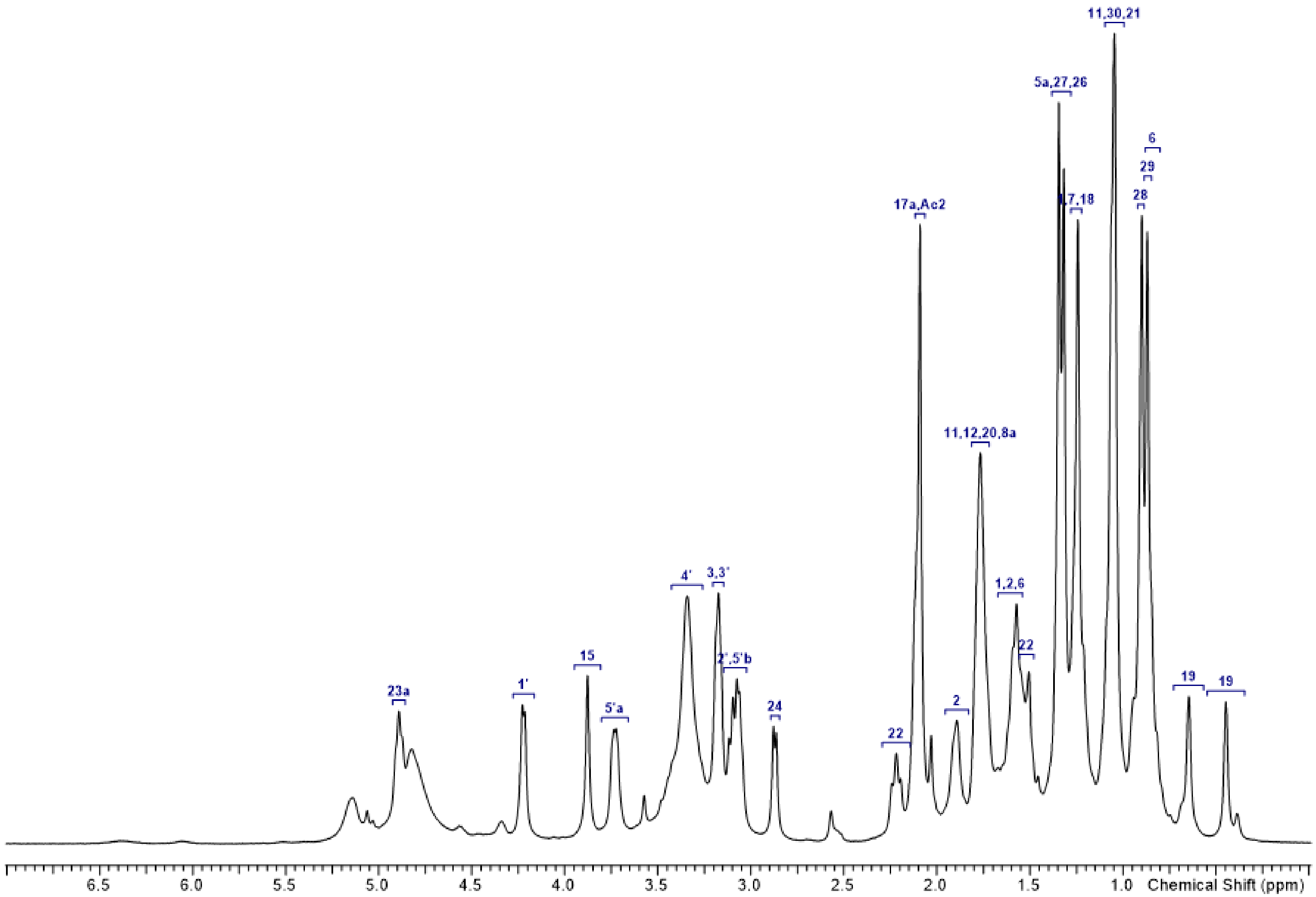

Figure S1. ^{1}H-NMR spectrum of compound **5b** (500 MHz, DMSO-d_6).

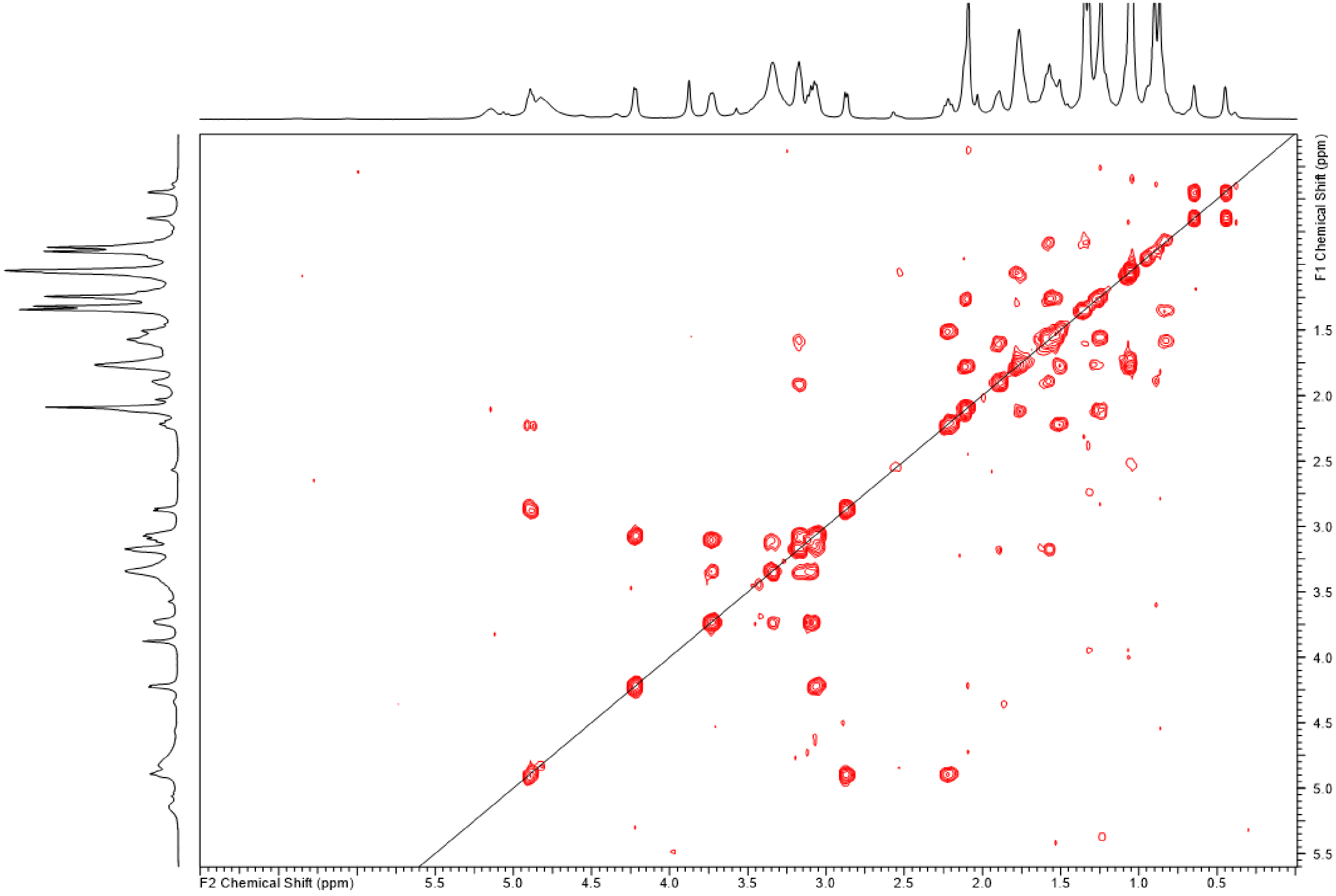

Figure S2. ^{1}H-^{1}H COSY spectrum of compound **5b** (500 MHz, DMSO-d_6).

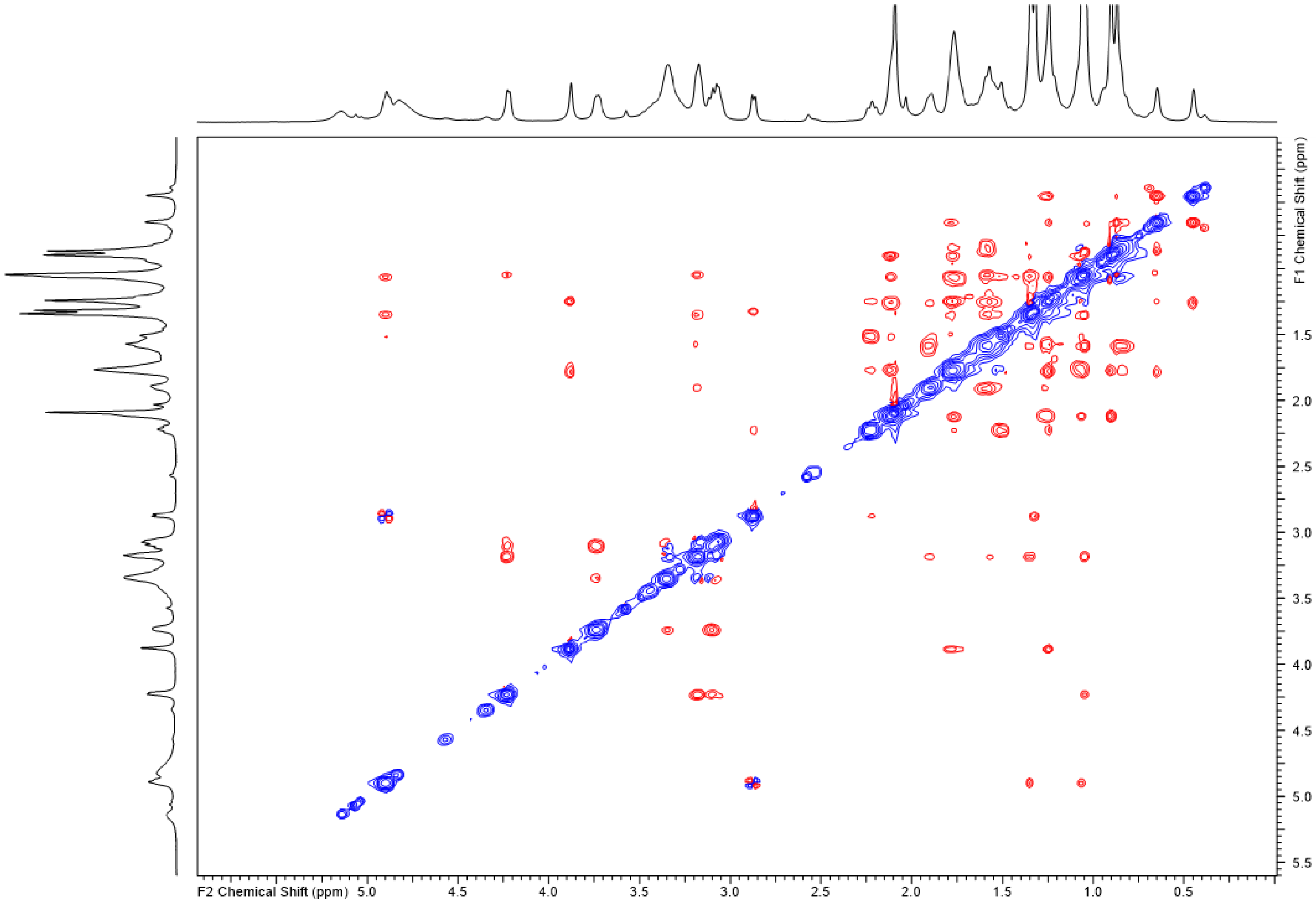

Figure S3. ^{1}H-^{1}H ROESY spectrum of compound **5b** (500 MHz, DMSO-d_6).

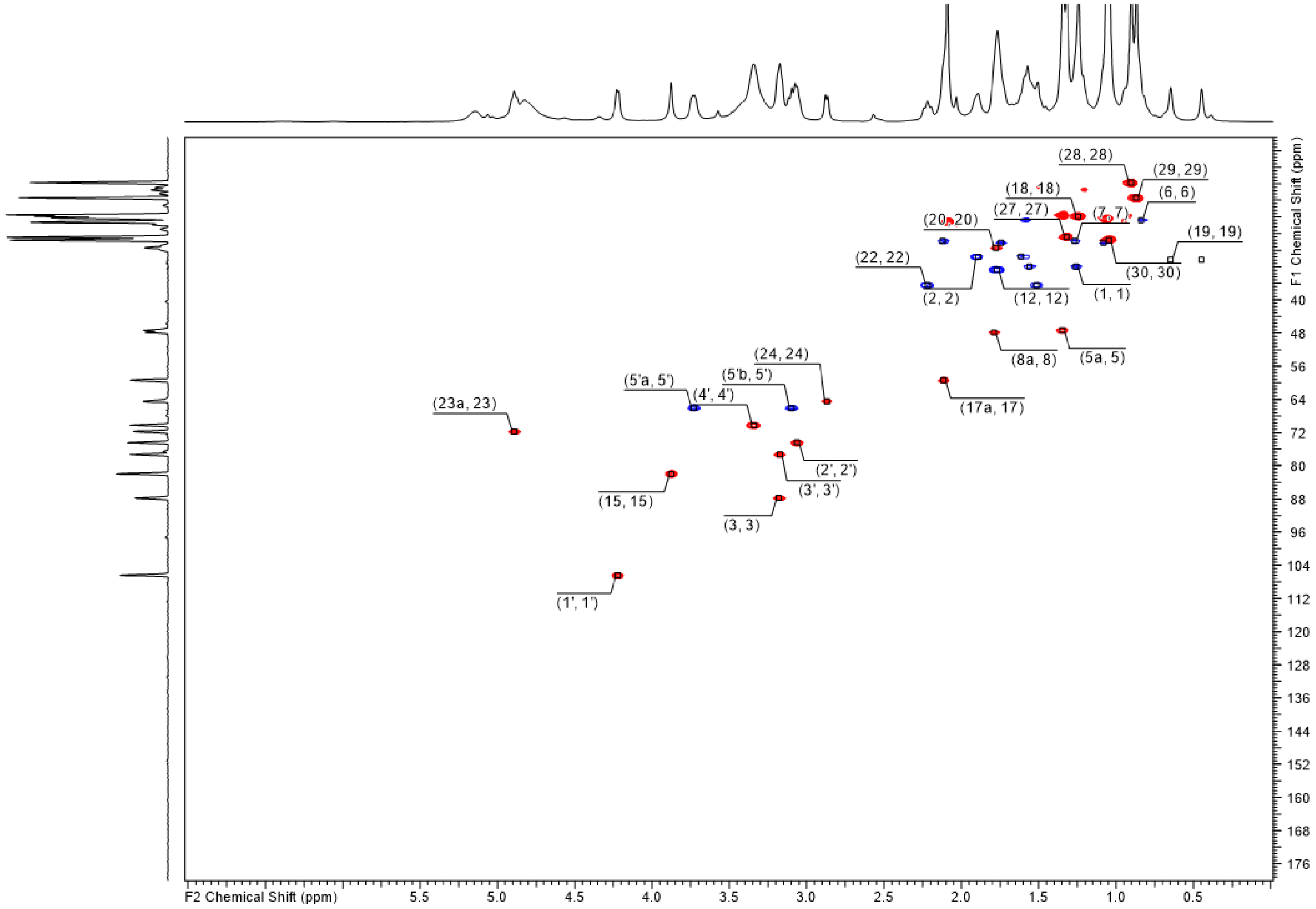

Figure S4. HSQC-DEPT spectrum of compound **5b** (500 MHz, DMSO-d$_6$).

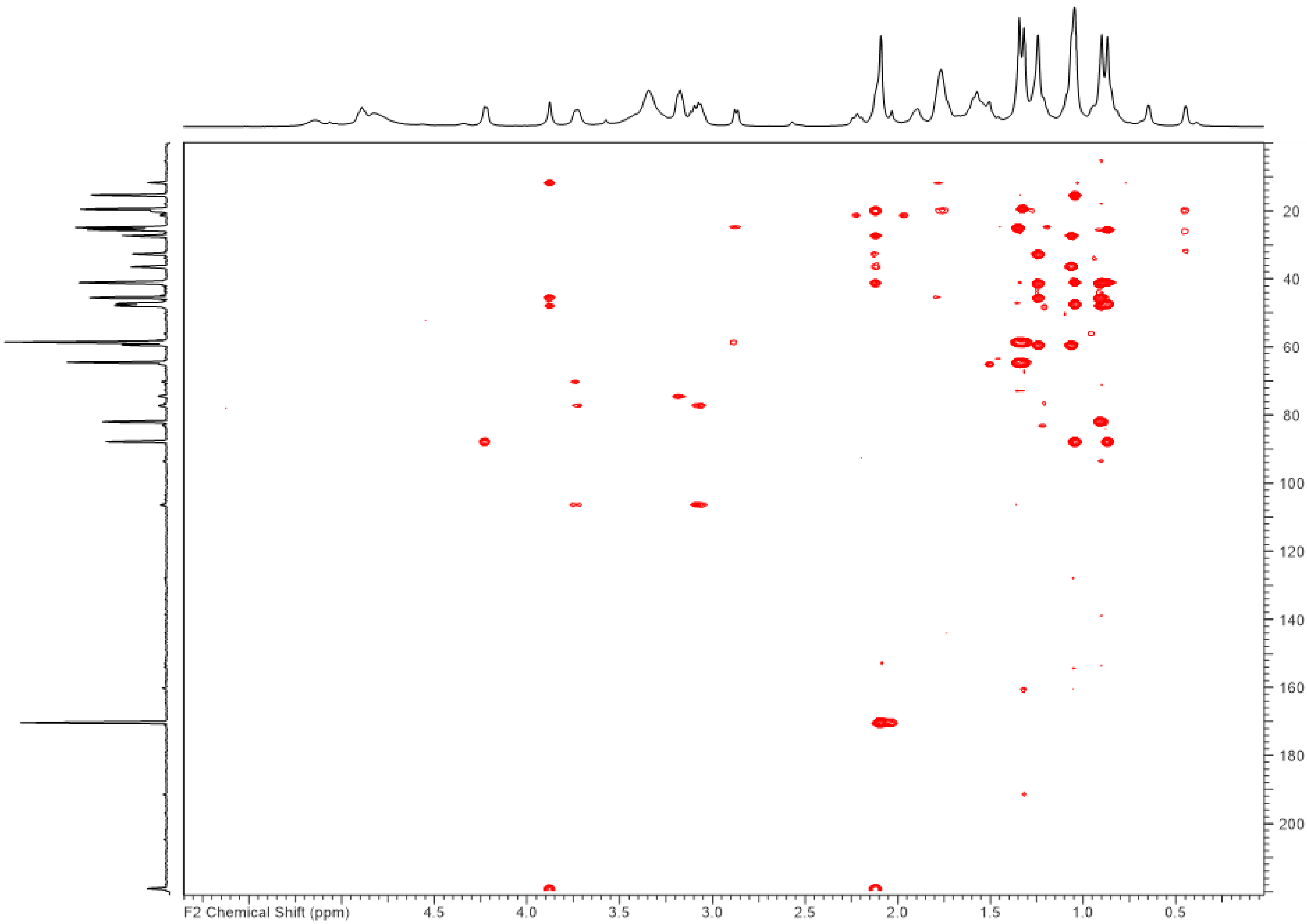

Figure S5. HMBC spectrum of compound **5b** (500 MHz, DMSO-d_6).

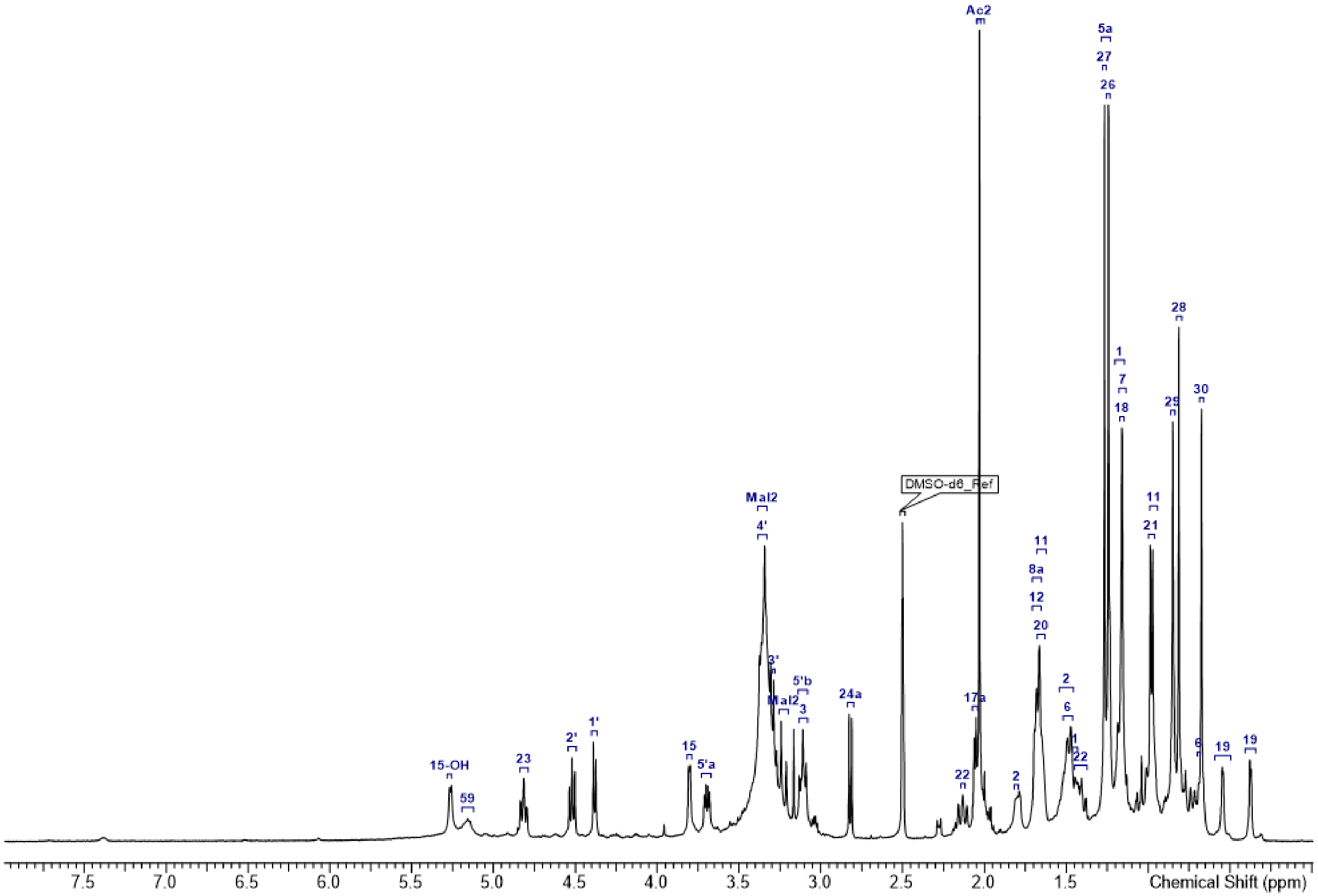

Figure S6. ^{1}H-NMR spectrum of compound **8a** (500 MHz, DMSO-d_6).

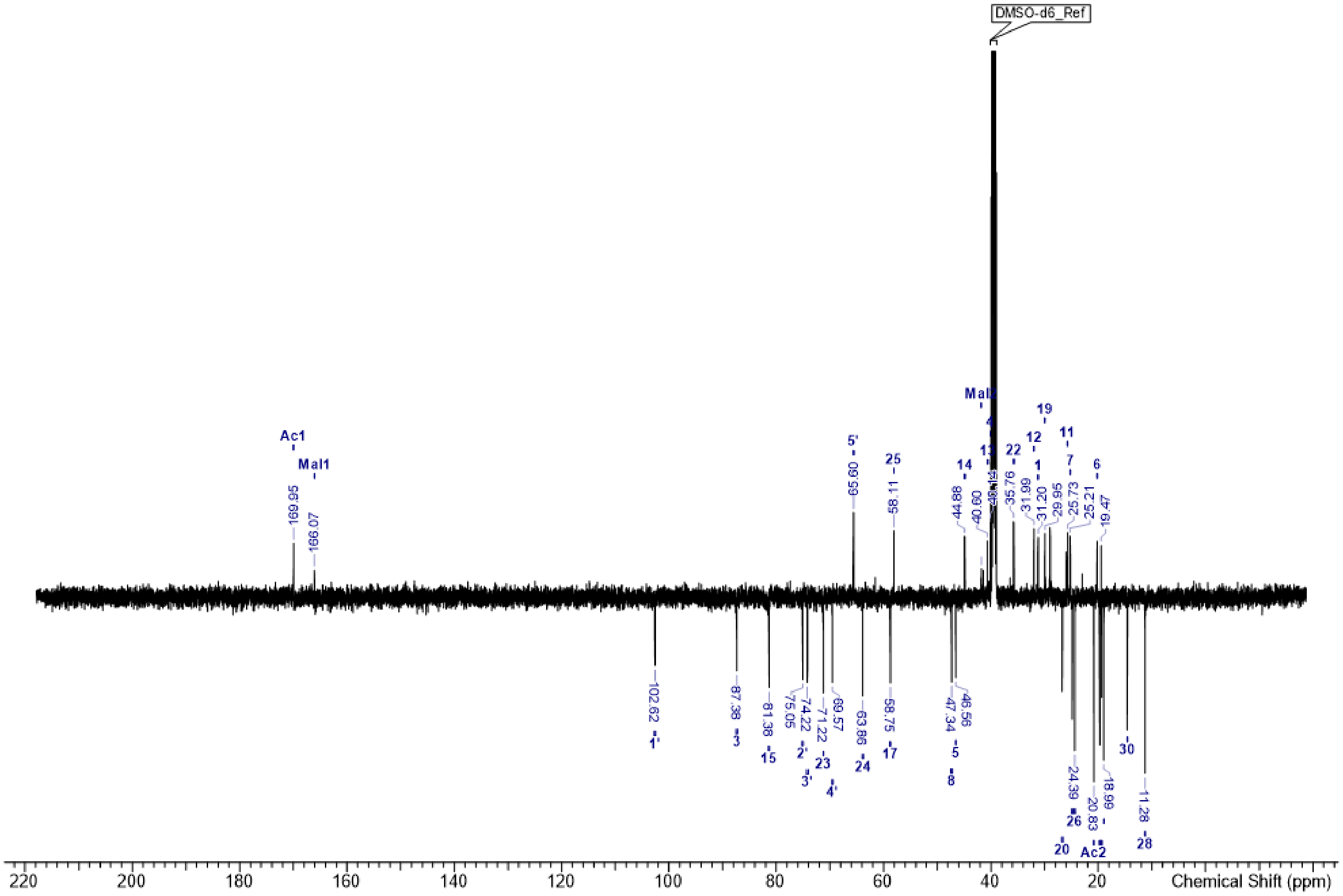

Figure S7. ^{13}C-DEPTq spectrum of compound **8a** (500 MHz, DMSO-d_6).

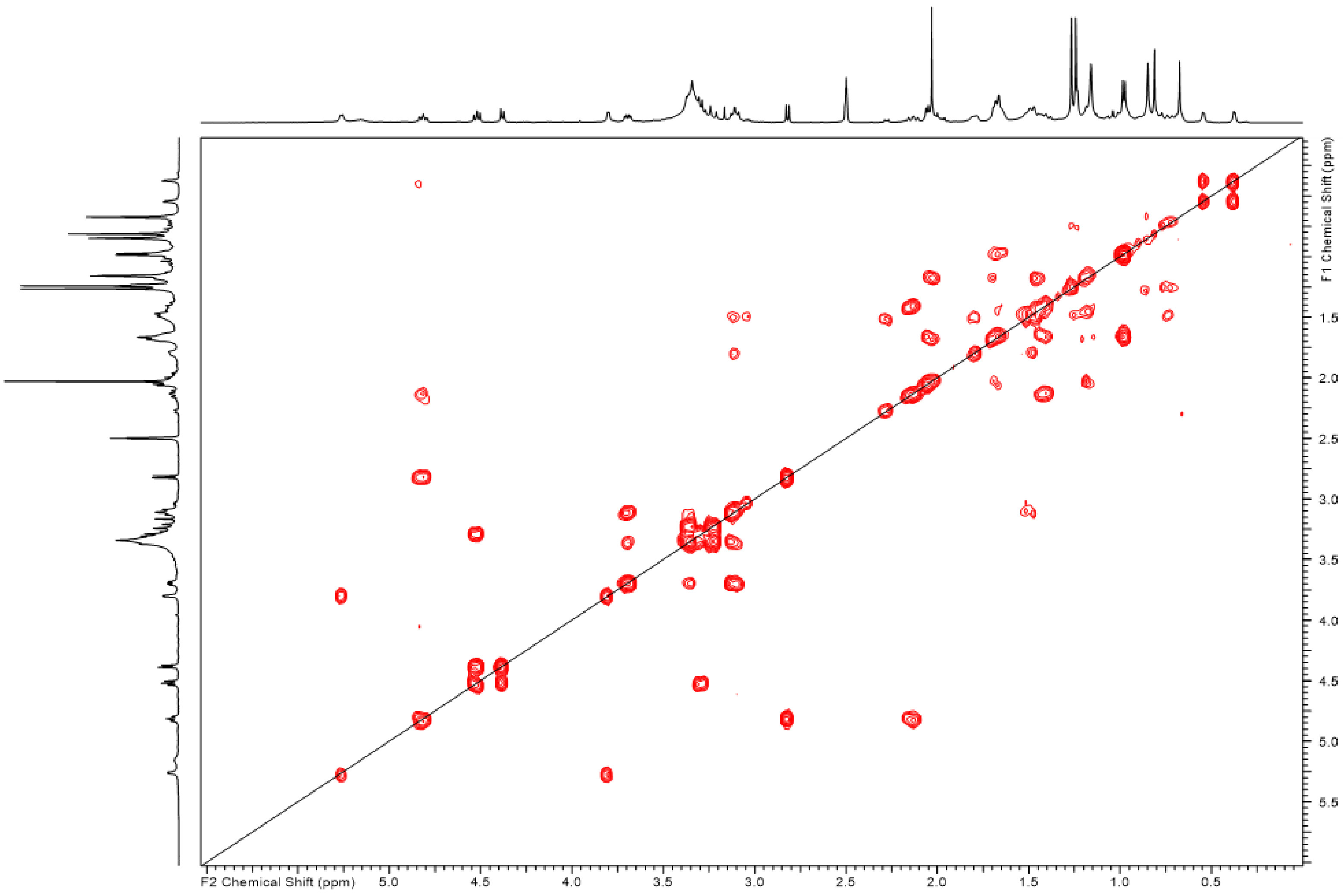

Figure S8. ^{1}H-^{1}H COSY spectrum of compound **8a** (500 MHz, DMSO-d_6).

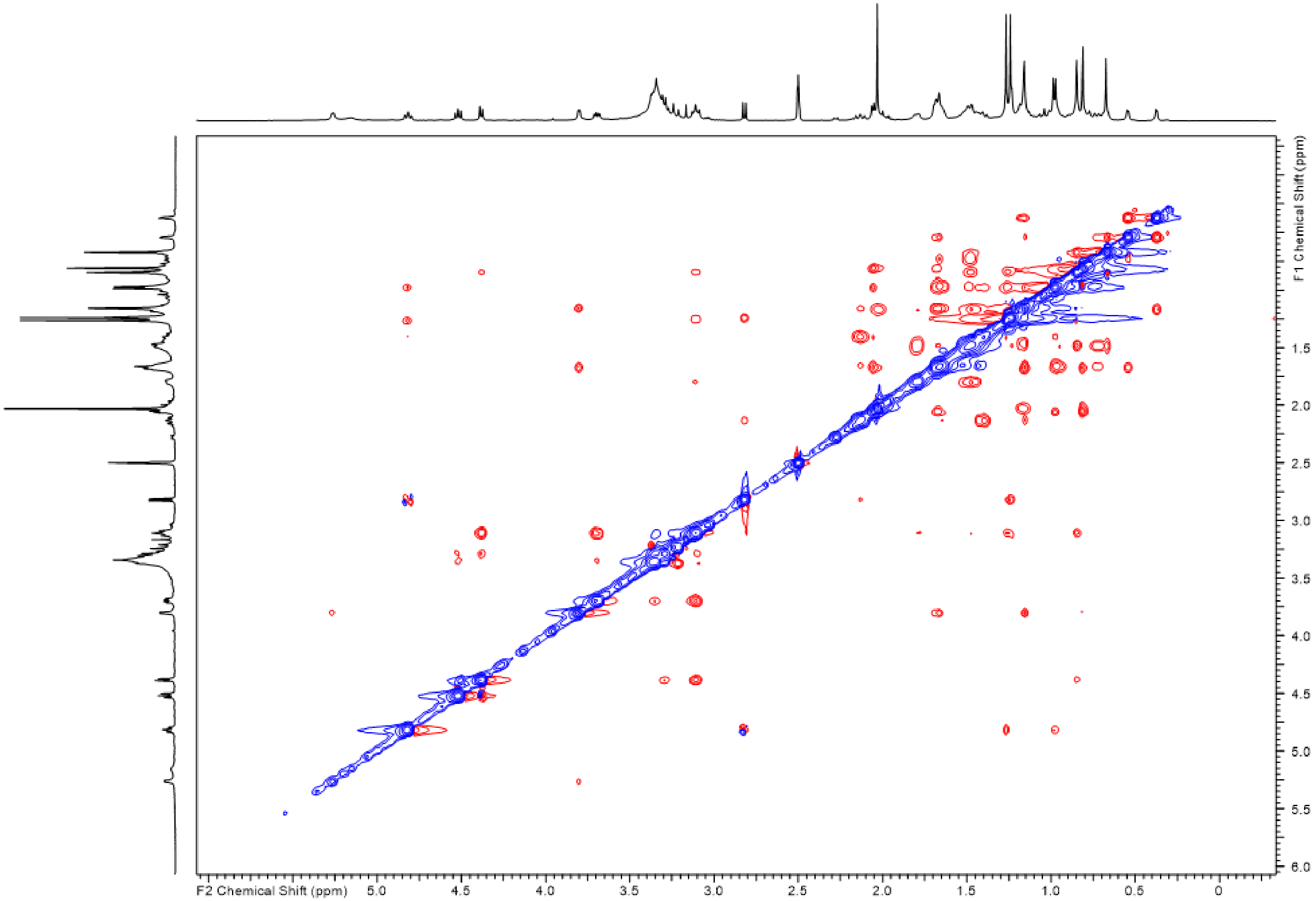

Figure S9. ^{1}H-^{1}H ROESY spectrum of compound **8a** (500 MHz, DMSO-d_6).

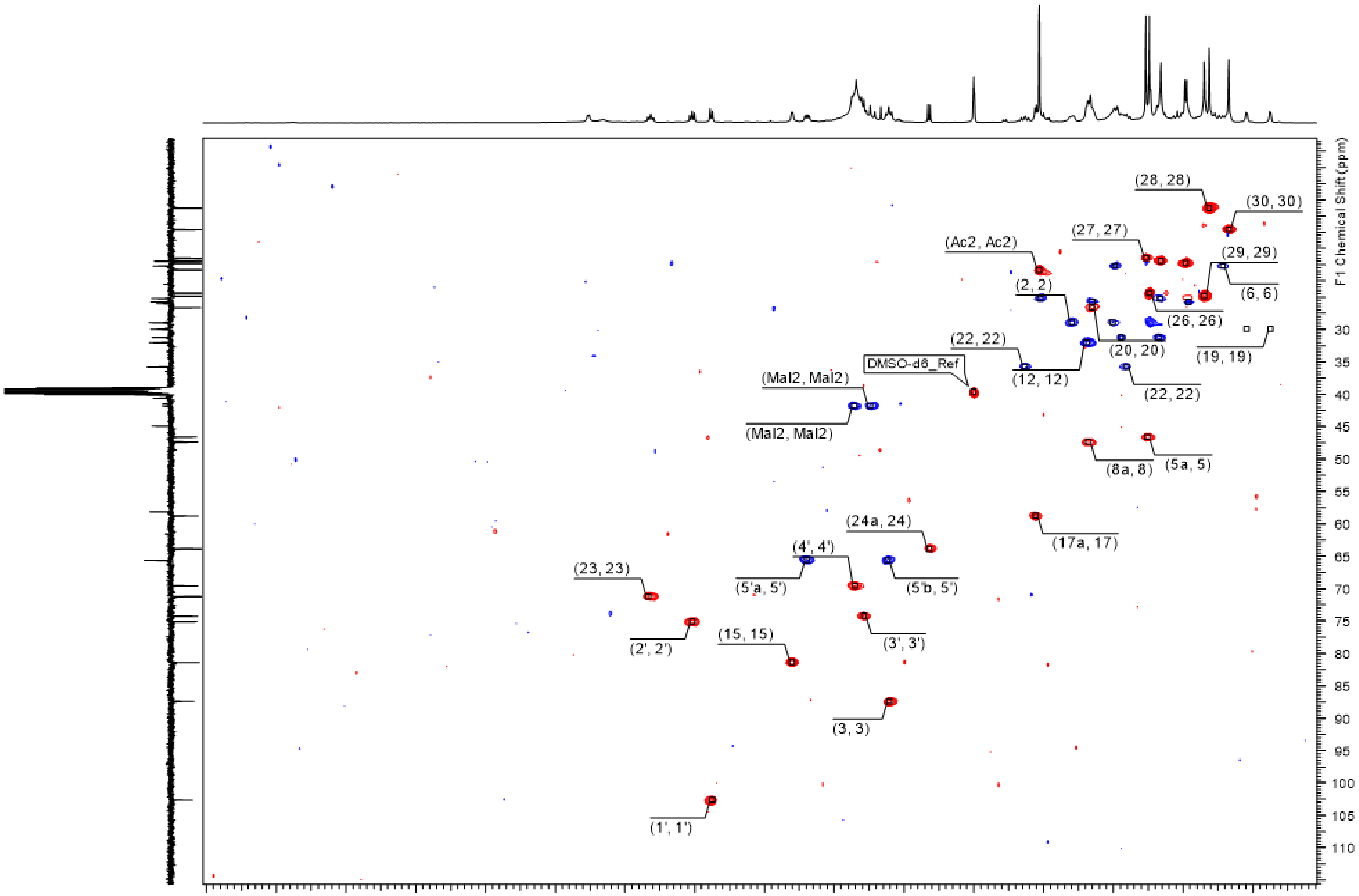

Figure S10. HSQC-DEPT spectrum of compound **8a** (500 MHz, DMSO-d_6).

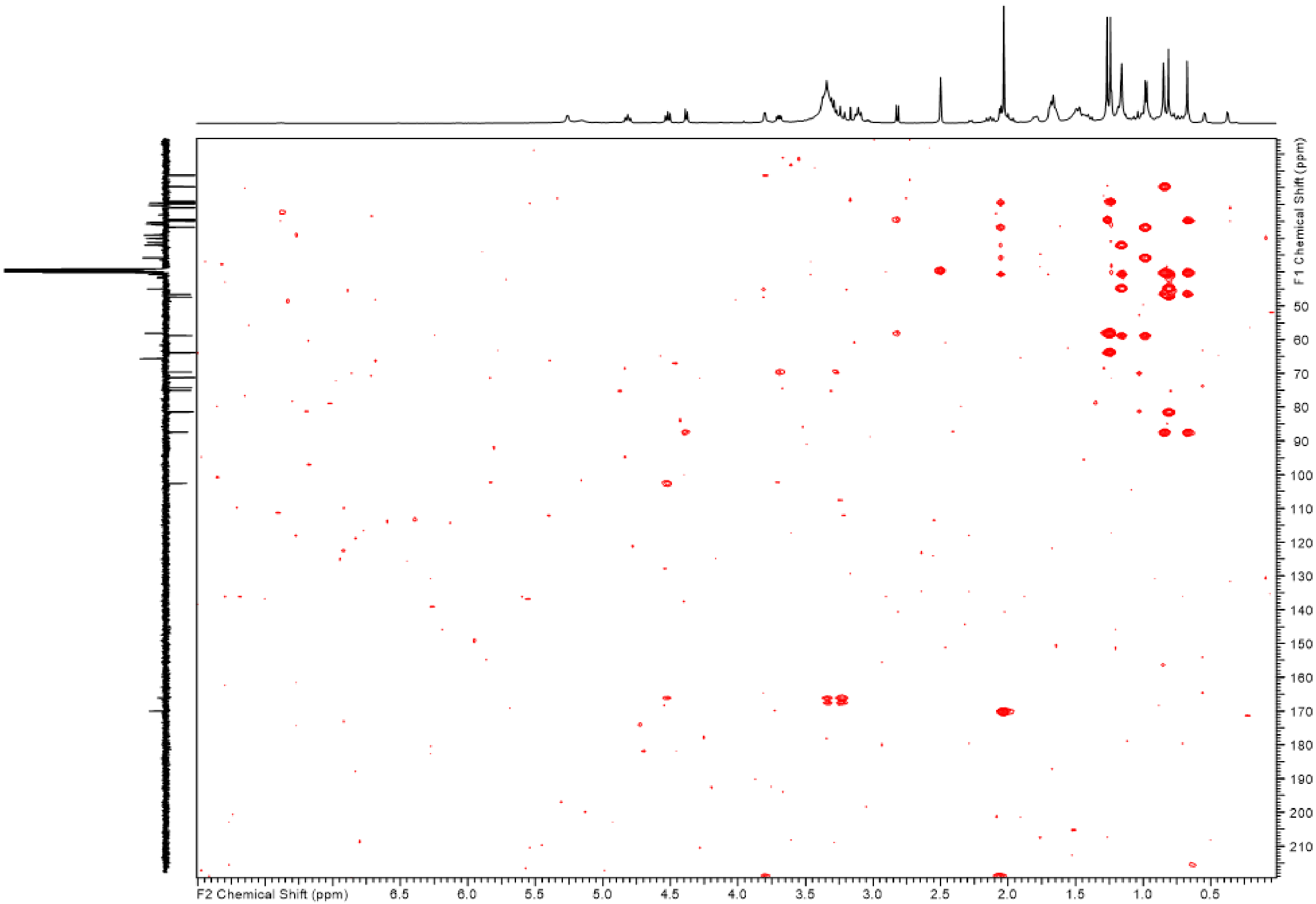

Figure S11. HMBC spectrum of compound **8a** (500 MHz, DMSO-d_6).

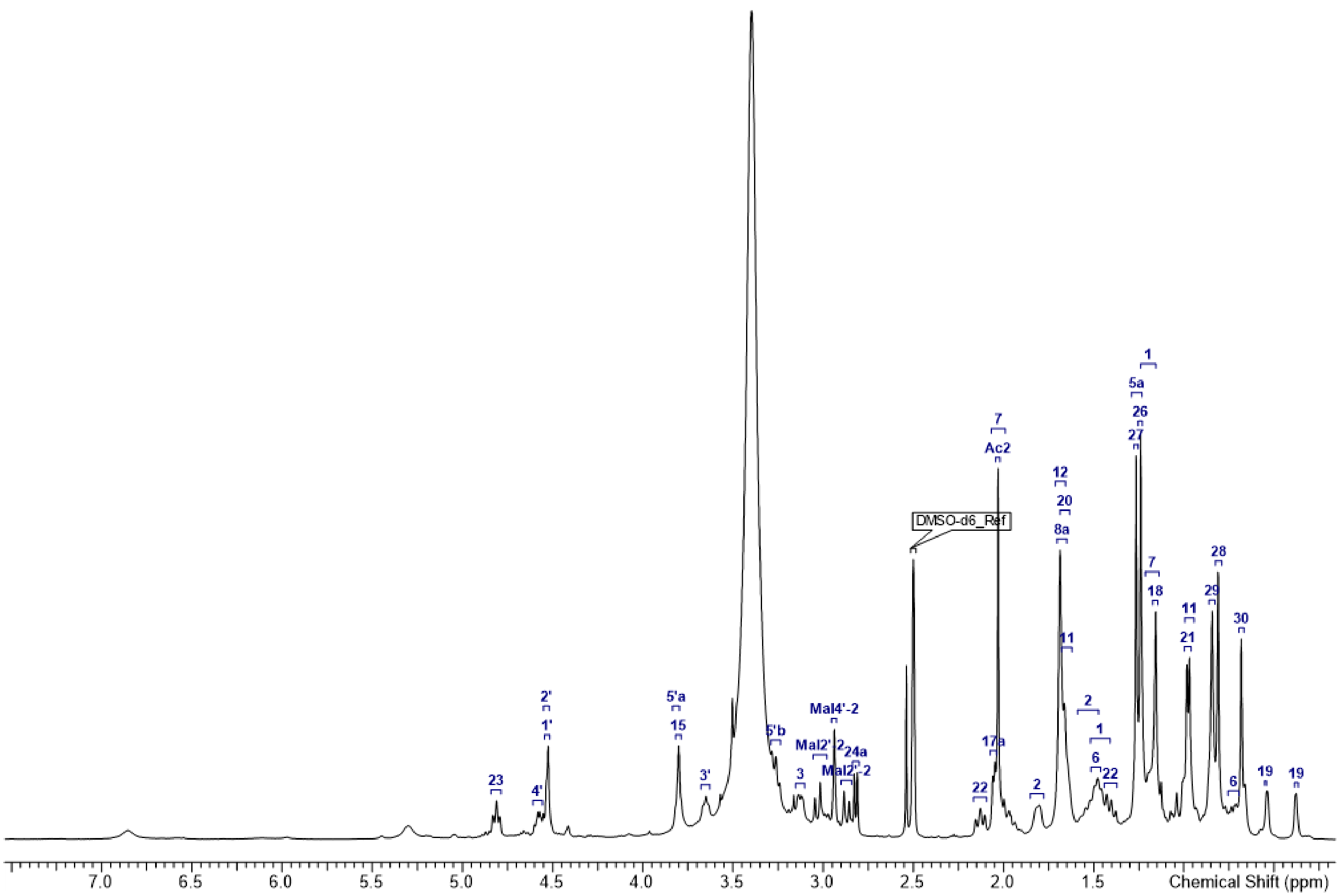

Figure S12. ^{1}H-NMR spectrum of compound **9a** (500 MHz, DMSO-d_6).

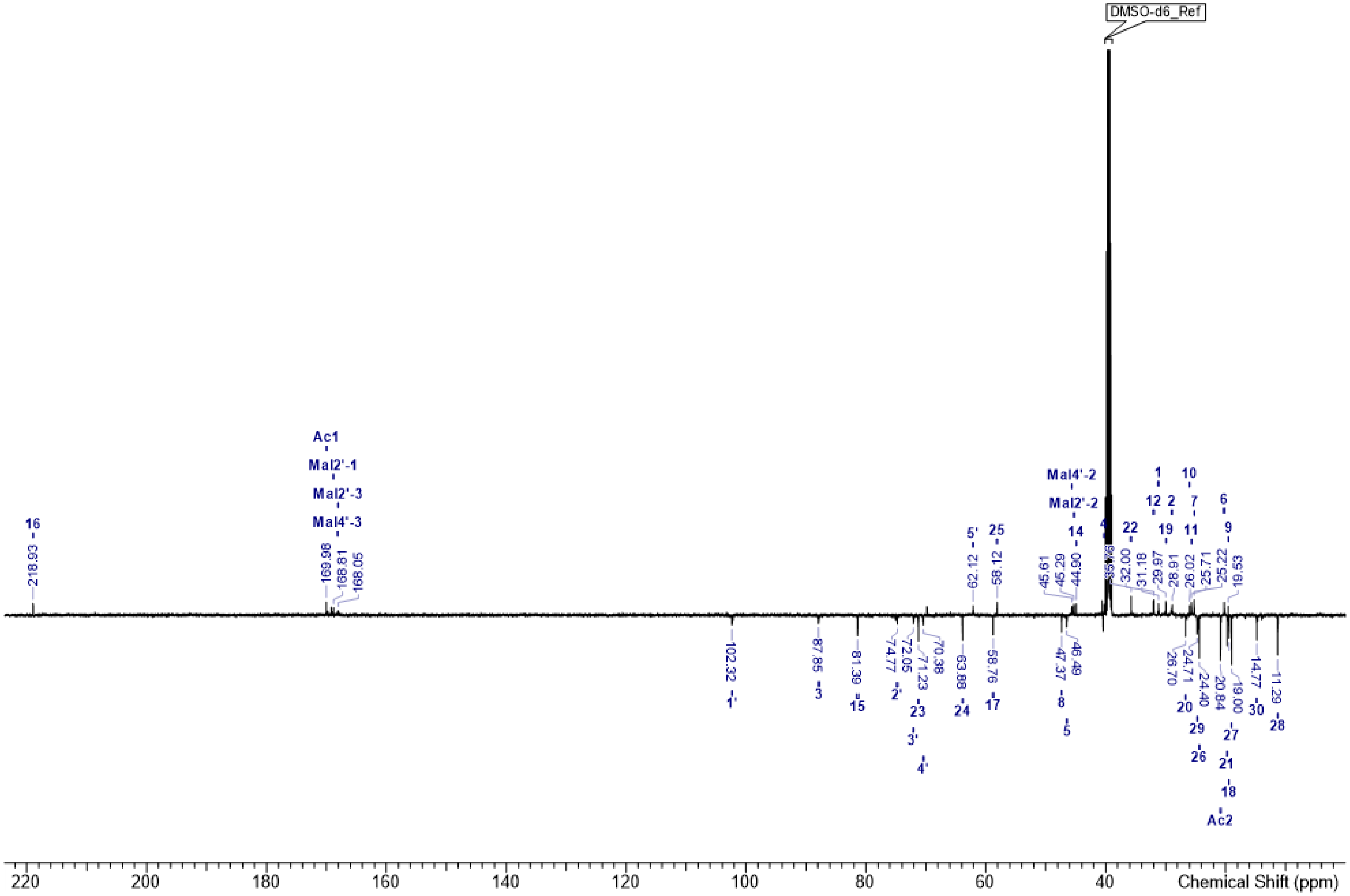

Figure S13. ^{13}C-DEPTq spectrum of compound **9a** (500 MHz, DMSO-d$_6$).

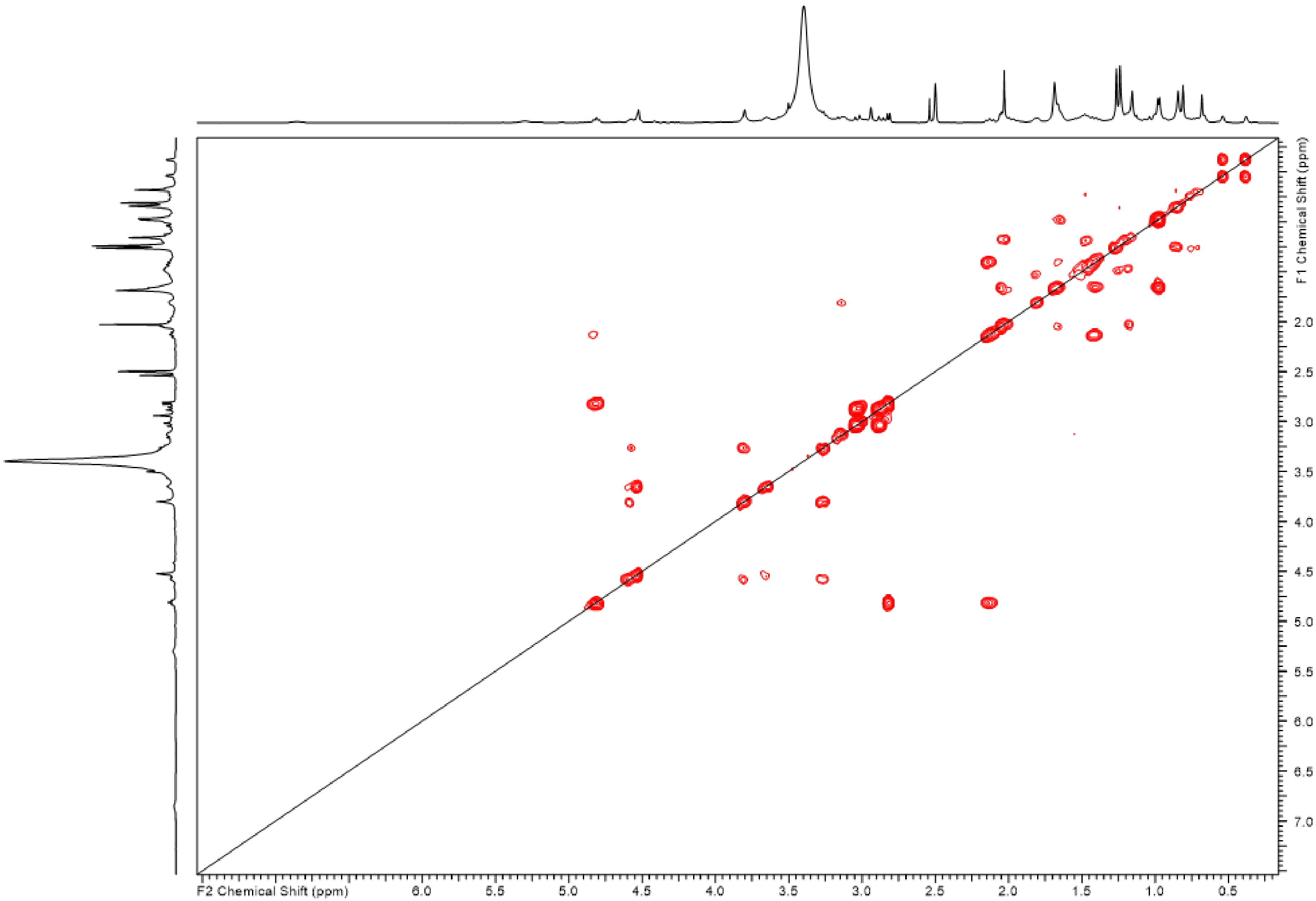

Figure S14. ^{1}H-^{1}H COSY spectrum of compound **9a** (500 MHz, DMSO-d_6).

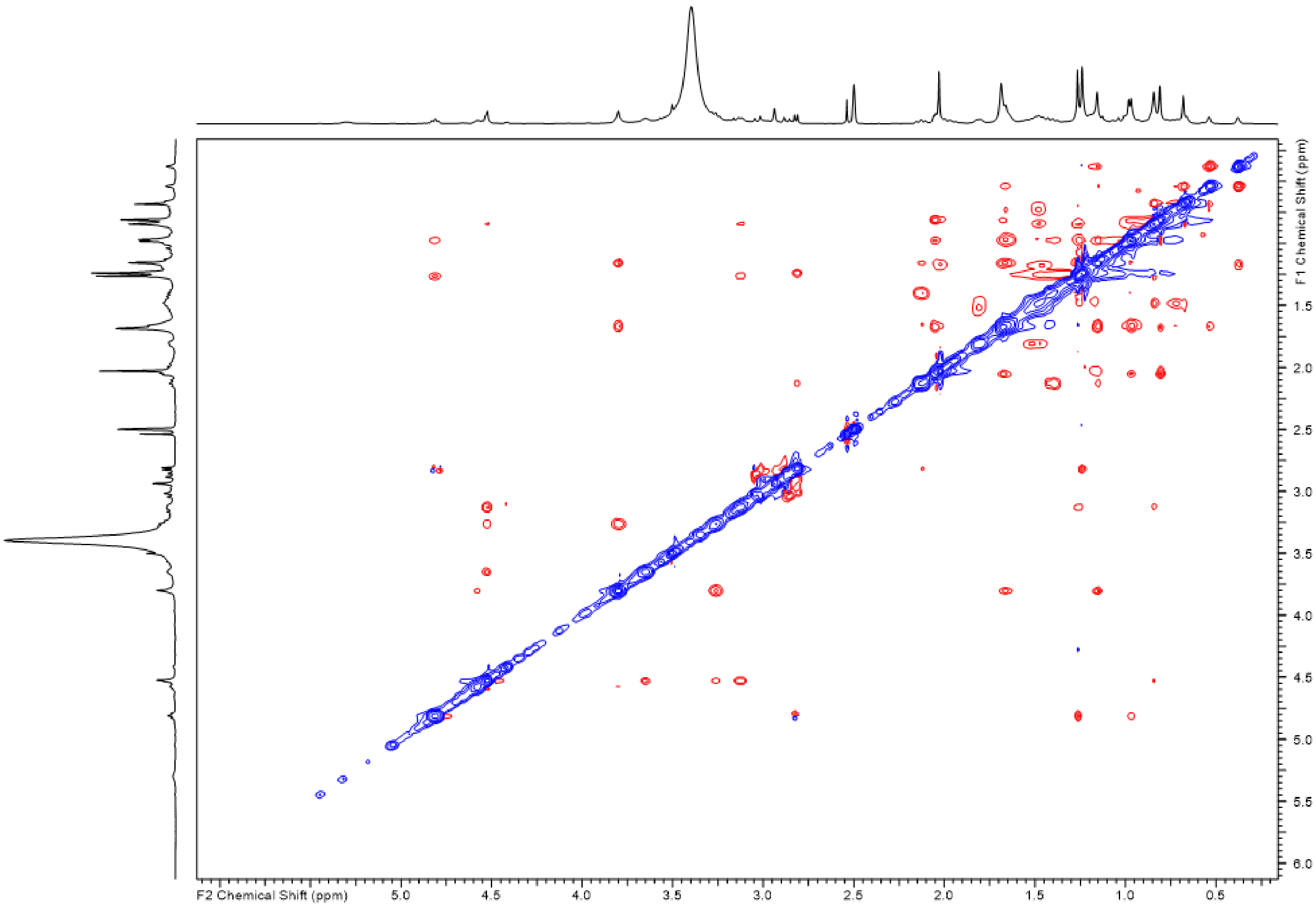

Figure S15. ^{1}H-^{1}H ROESY spectrum of compound **9a** (500 MHz, DMSO-d$_6$).

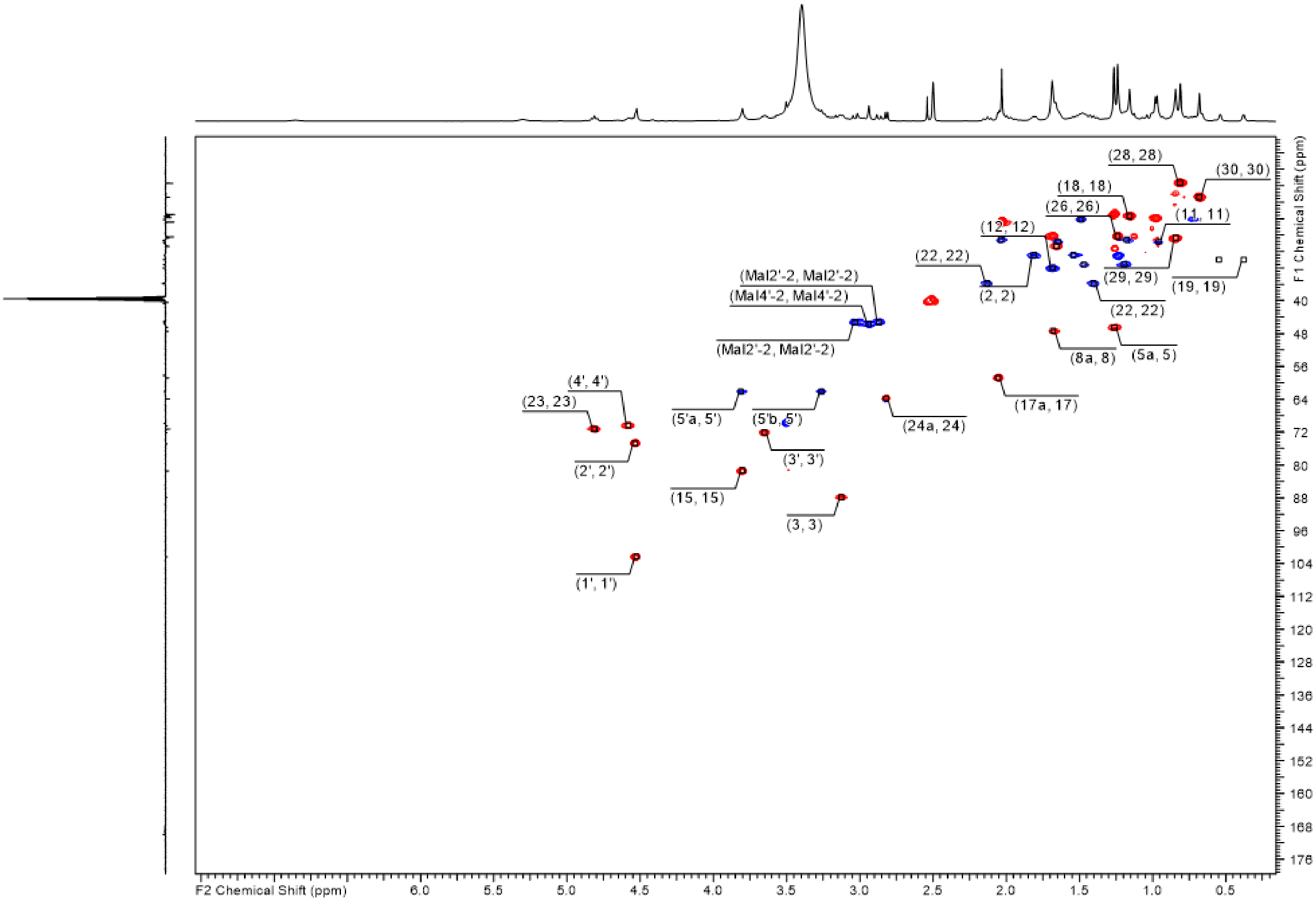

Figure S16. HSQC-DEPT spectrum of compound **9a** (500 MHz, DMSO-d$_6$).

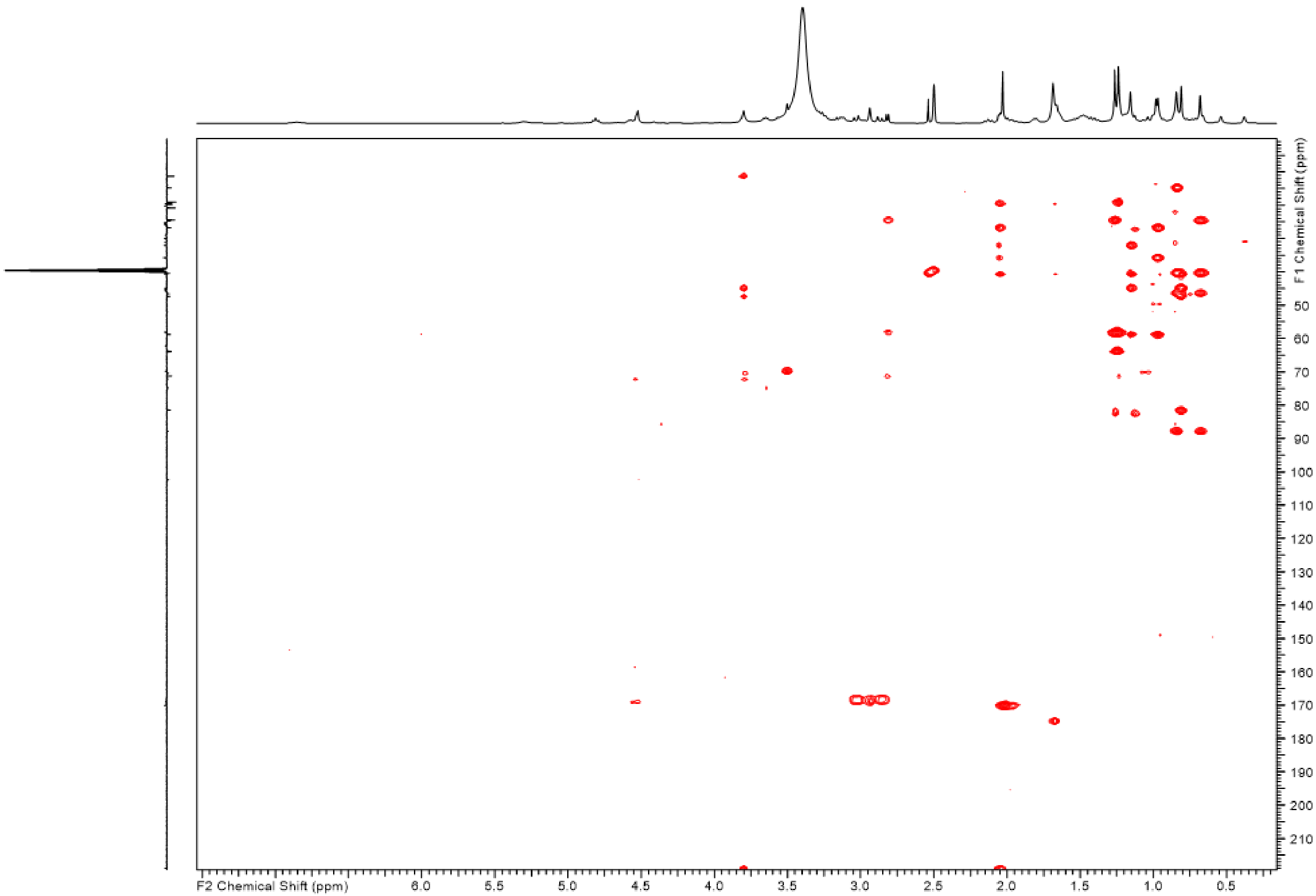

Figure S17. HMBC spectrum of compound **9a** (500 MHz, DMSO-d_6).

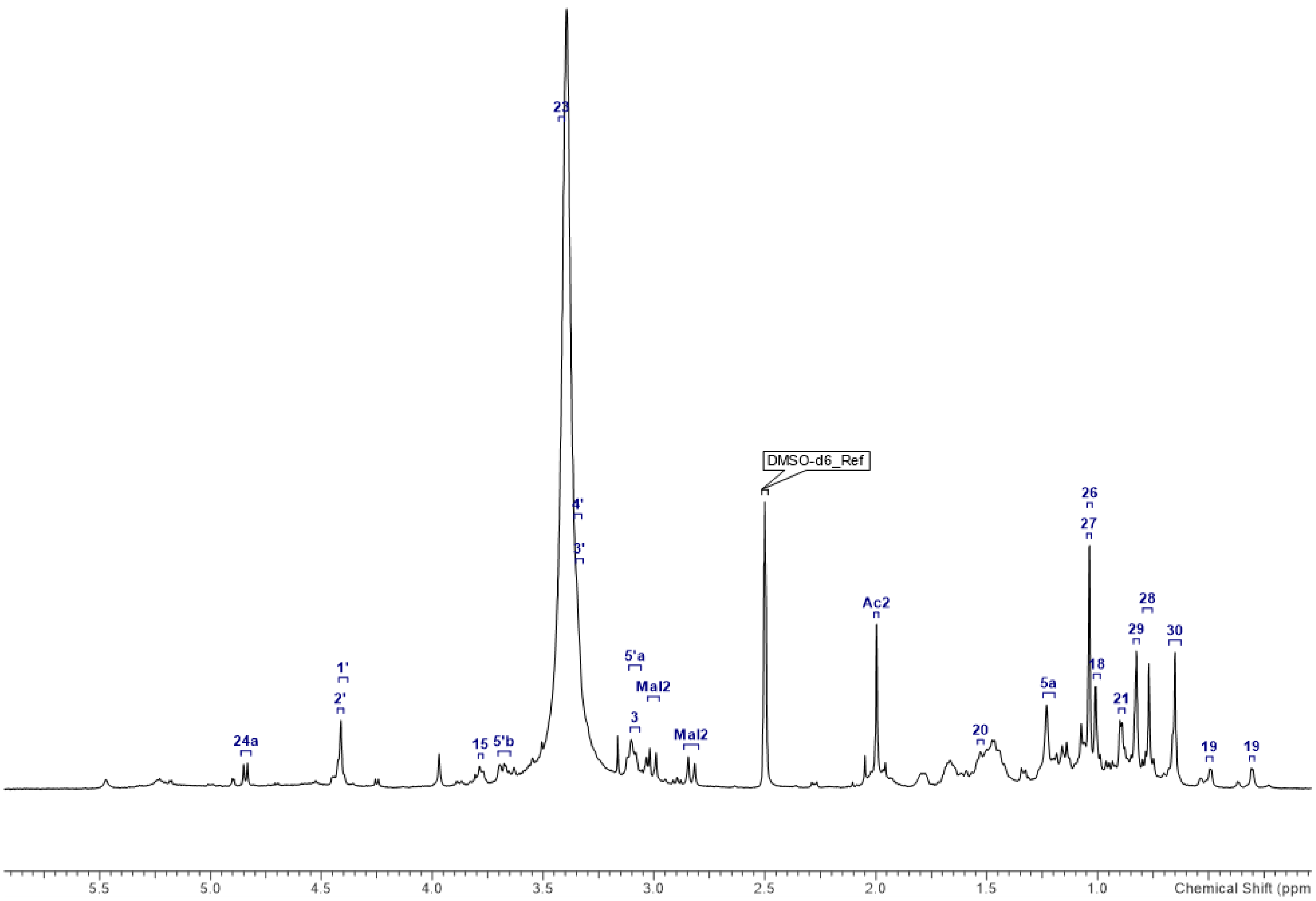

Figure S18. ^{1}H-NMR spectrum of compound **10a** (500 MHz, DMSO-d_6).

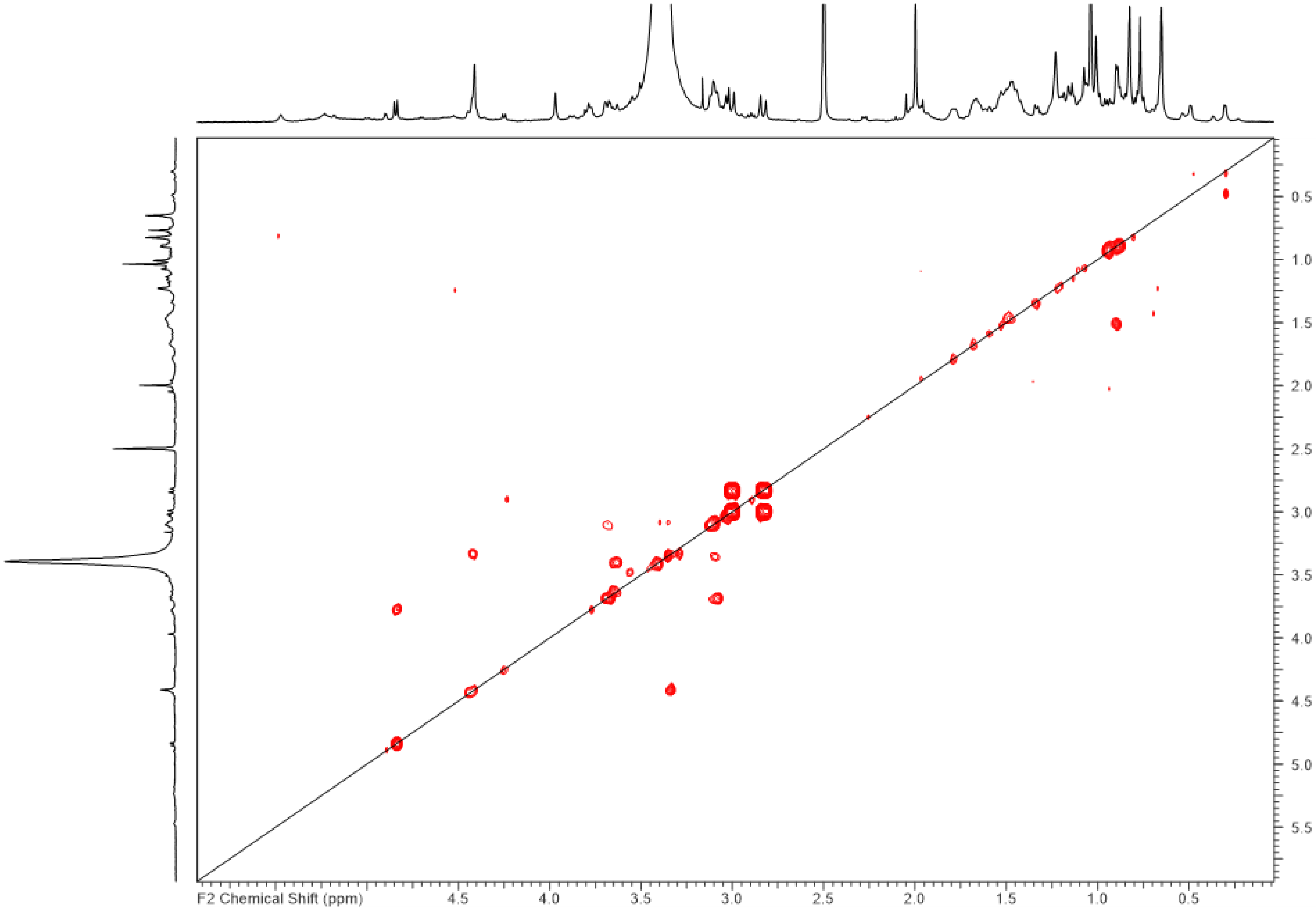

Figure S19. ^{1}H-^{1}H COSY spectrum of compound **10a** (500 MHz, DMSO-d_6).

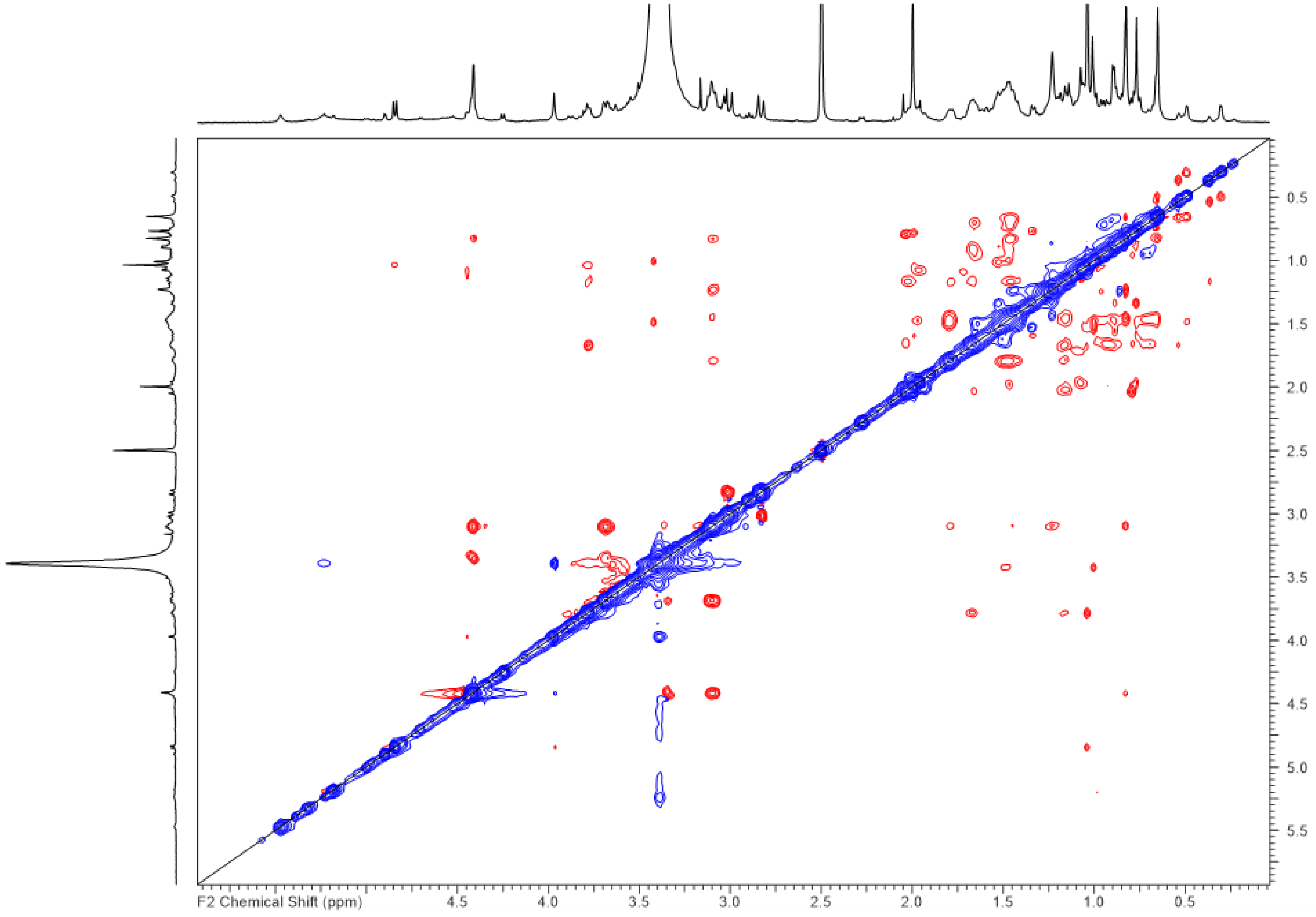

Figure S20. ^{1}H-^{1}H ROESY spectrum of compound **10a** (500 MHz, DMSO-d_6).

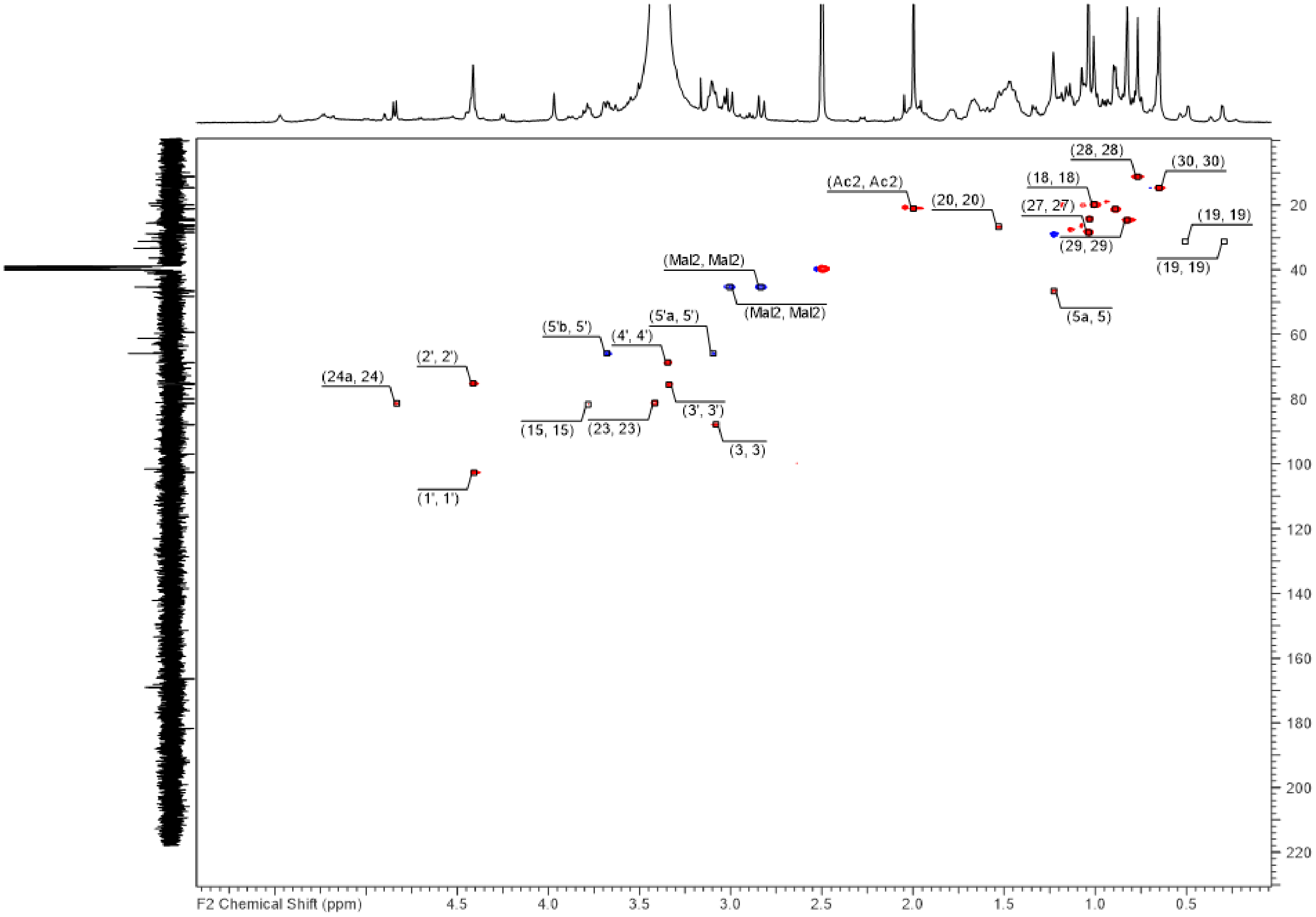

Figure S21. HSQC-DEPT spectrum of compound **10a** (500 MHz, DMSO-d_6).

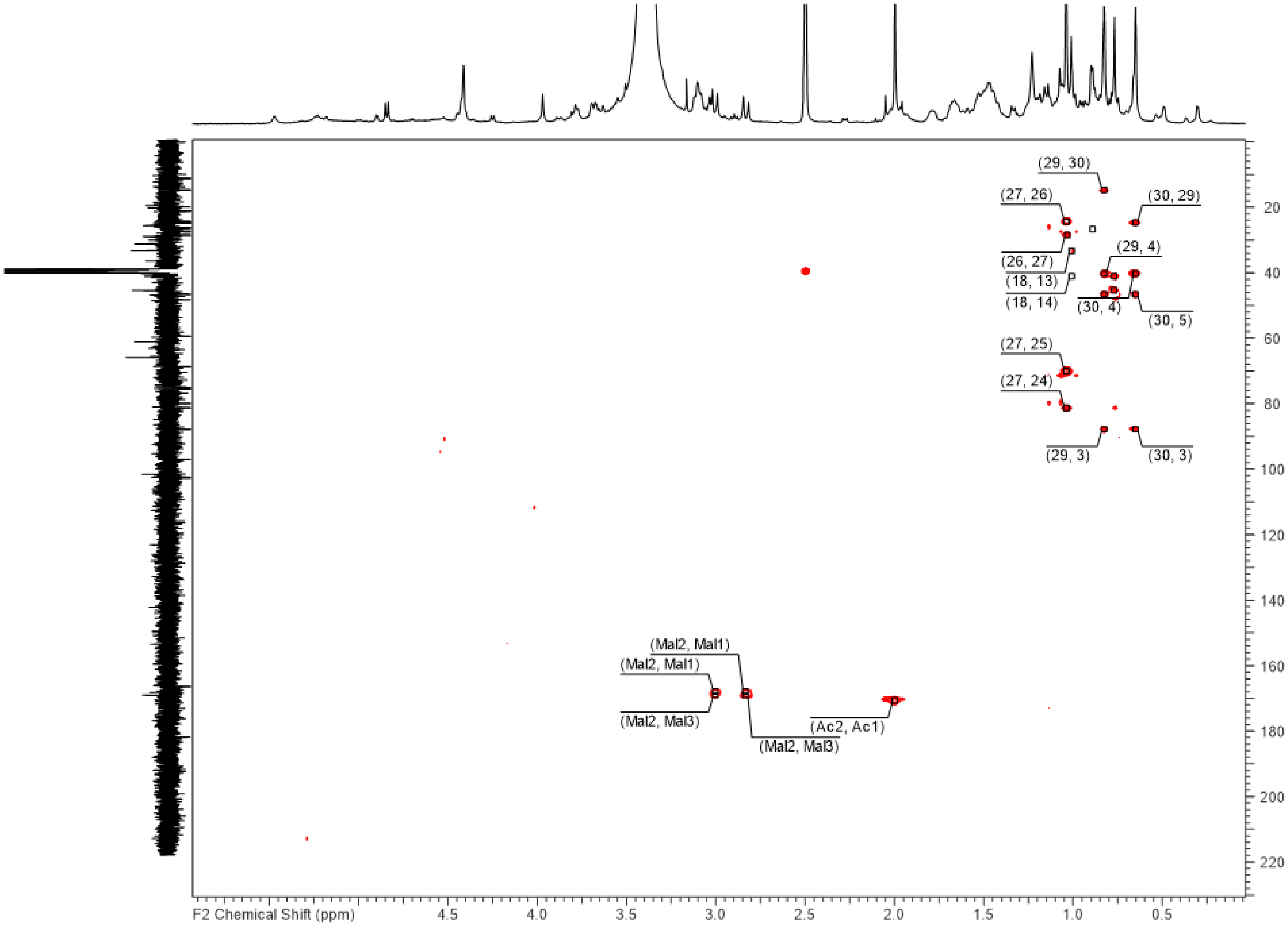

Figure S22. HMBC spectrum of compound **10a** (500 MHz, DMSO-d_6).

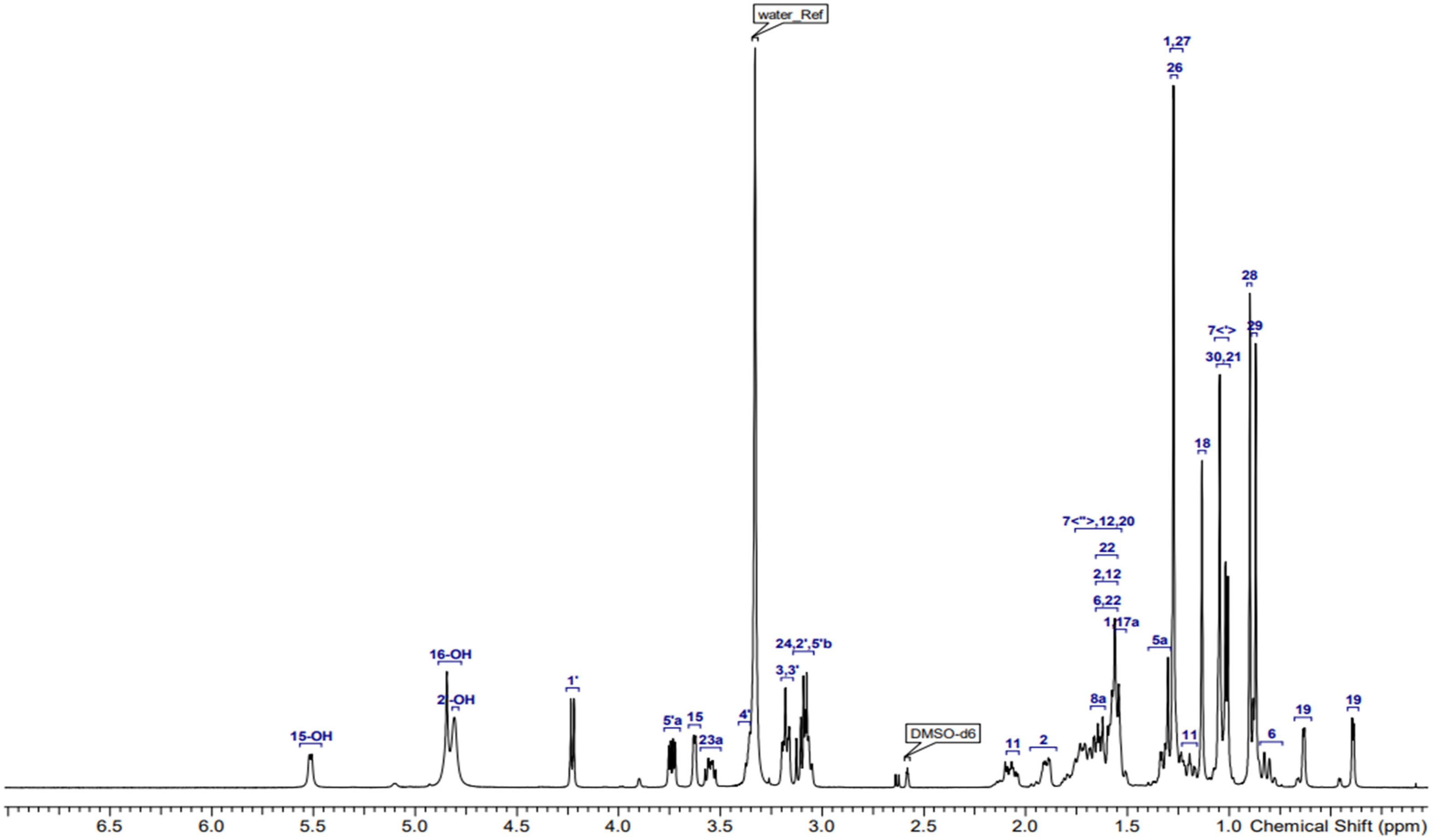

Figure S23. ^{1}H-NMR spectrum of compound **13** (500 MHz, DMSO-d_6).

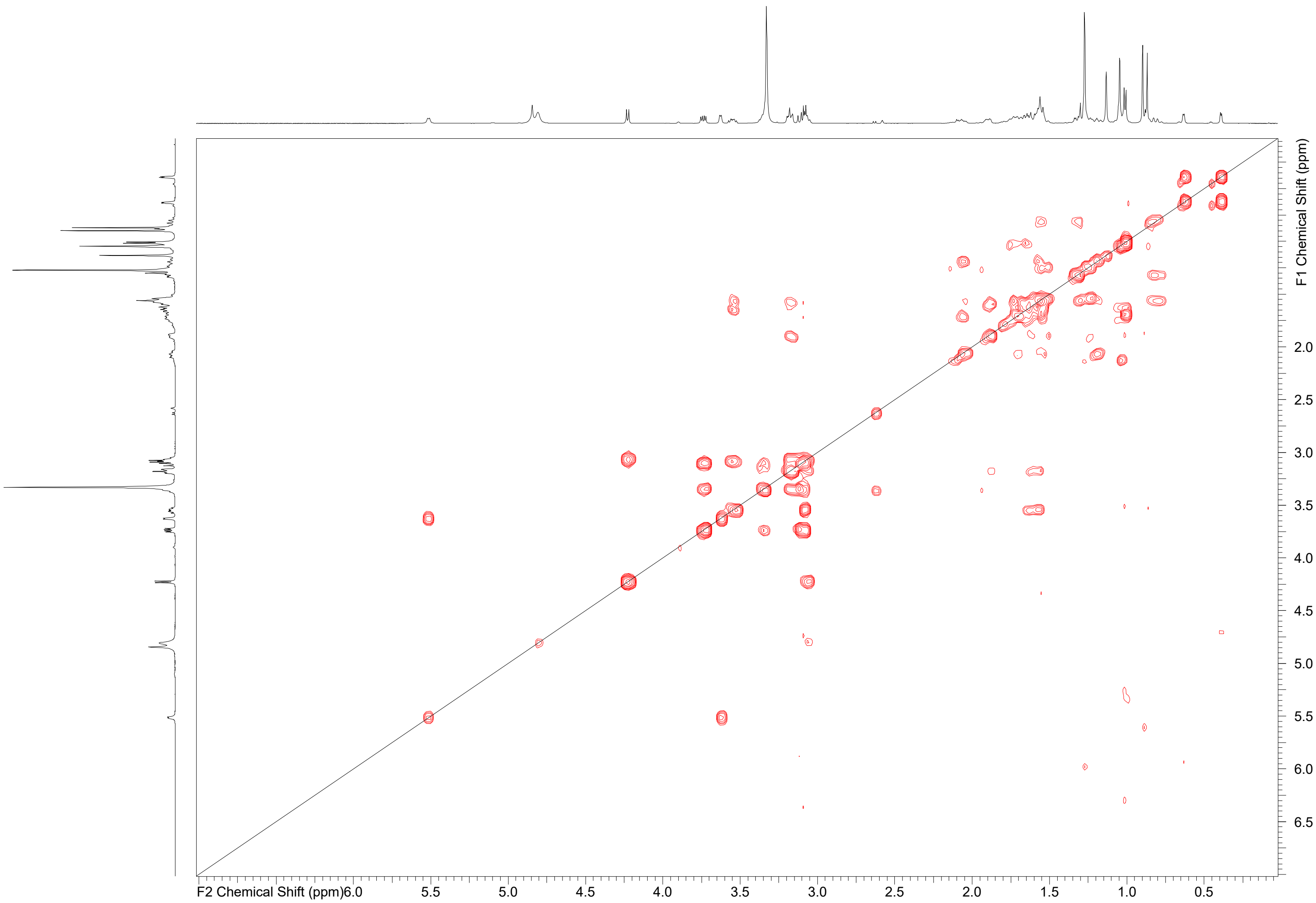

Figure S24. ^{1}H-^{1}H COSY spectrum of compound **13** (500 MHz, DMSO-d_6).

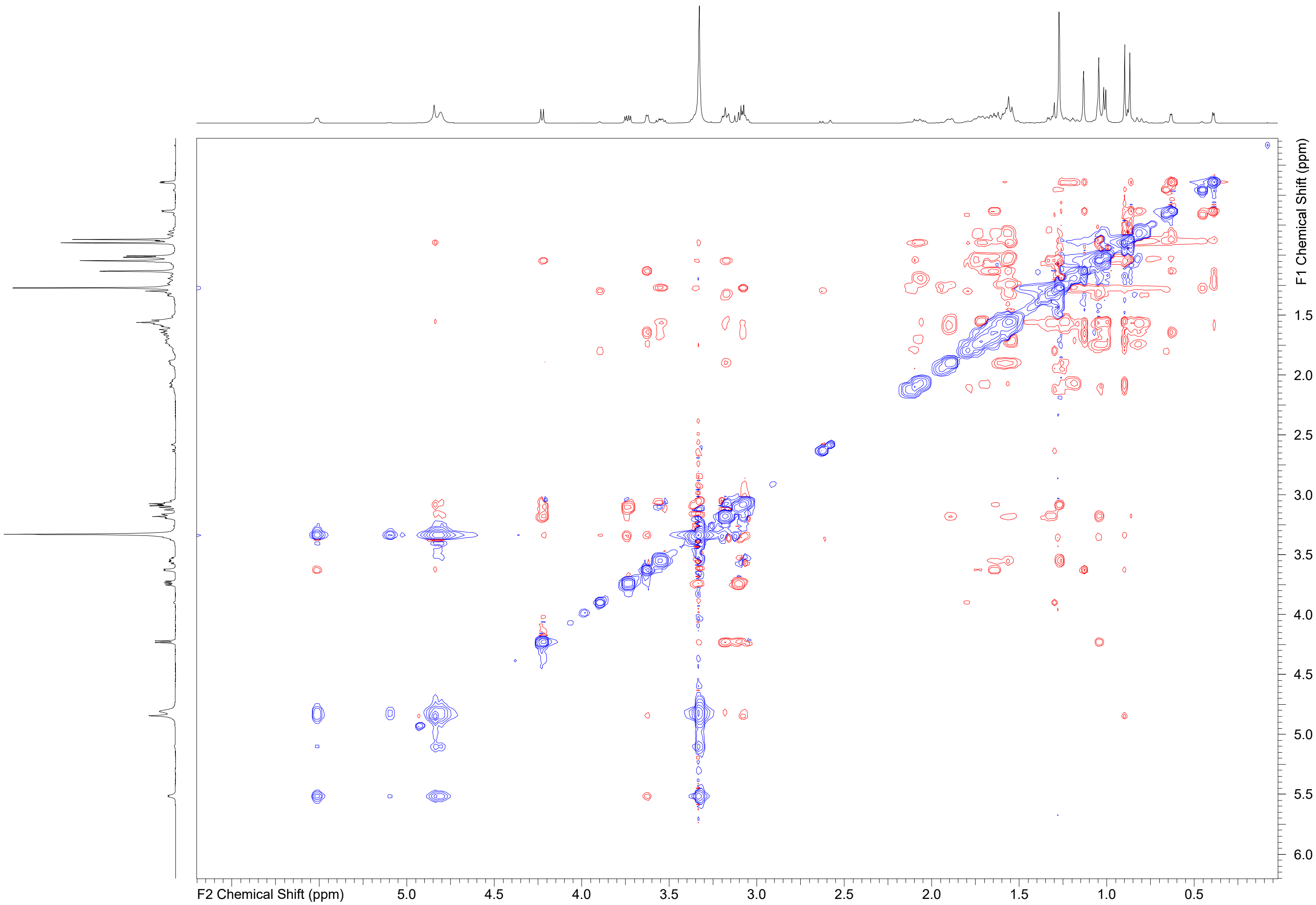

Figure S25. ^{1}H-^{1}H ROESY spectrum of compound **13** (500 MHz, DMSO-d_6).

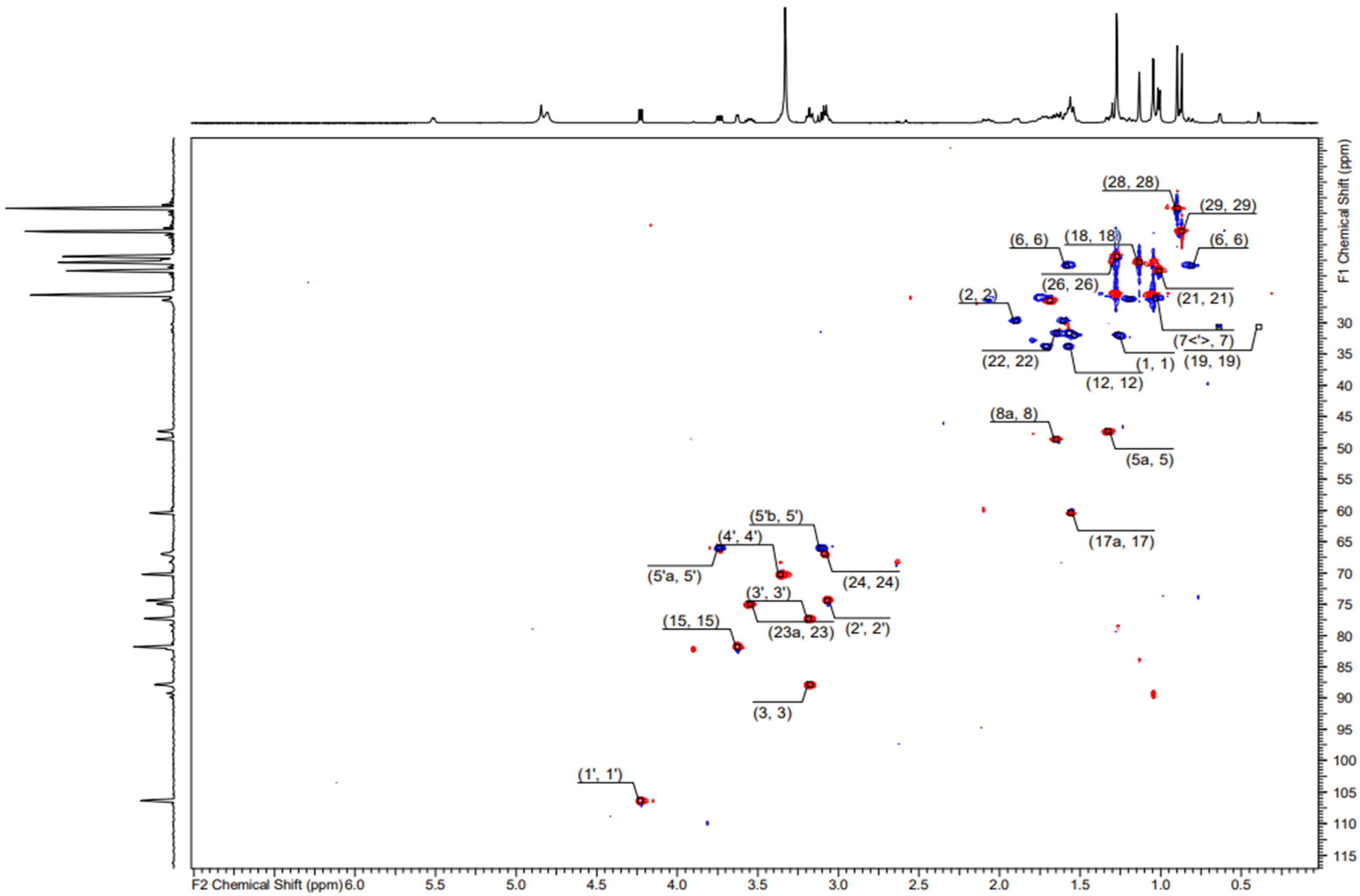

Figure S26. HSQC-DEPT spectrum of compound **13** (500 MHz, DMSO-d_6).

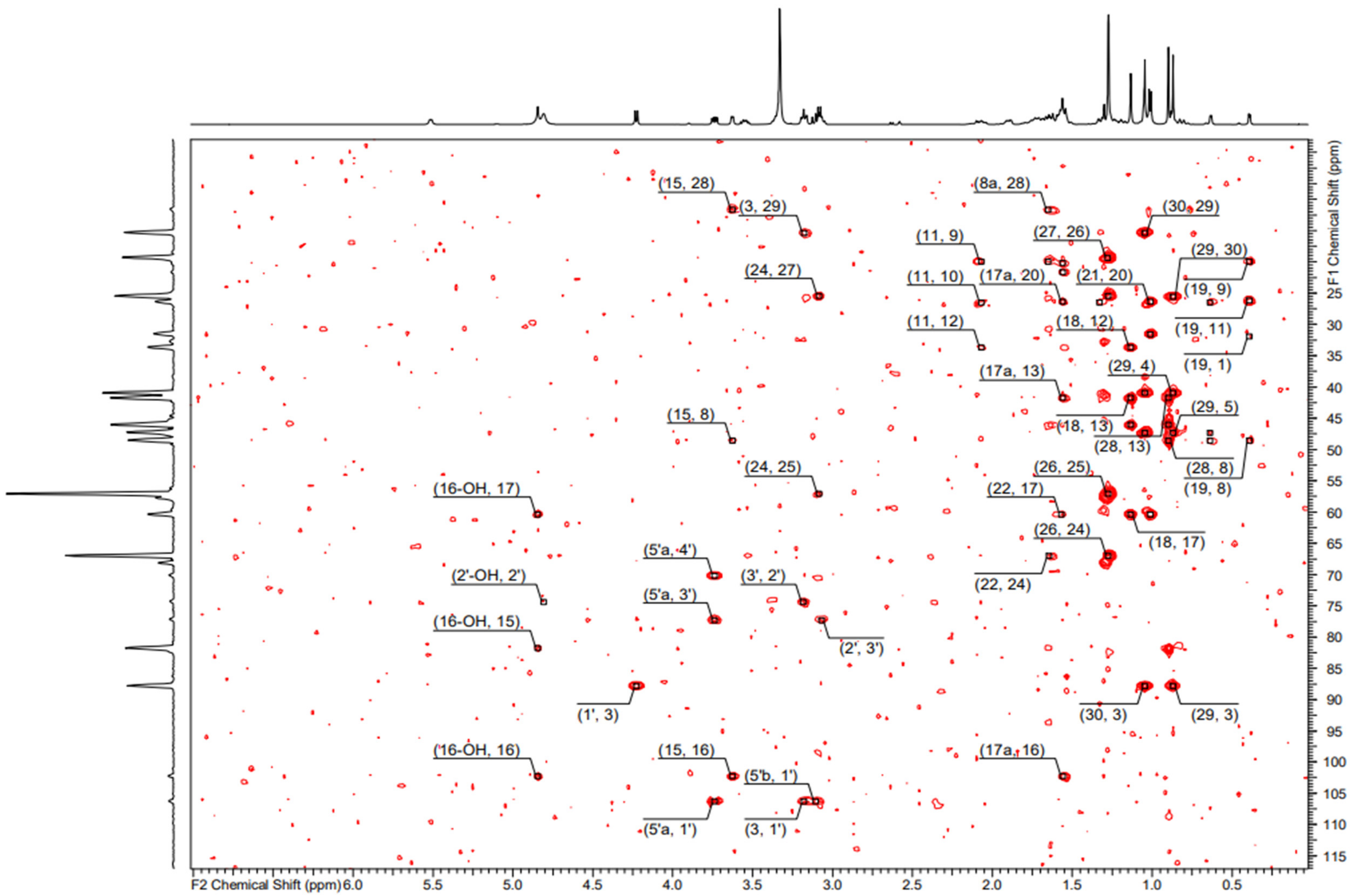

Figure S27. HMBC spectrum of compound **13** (500 MHz, DMSO-d_6).

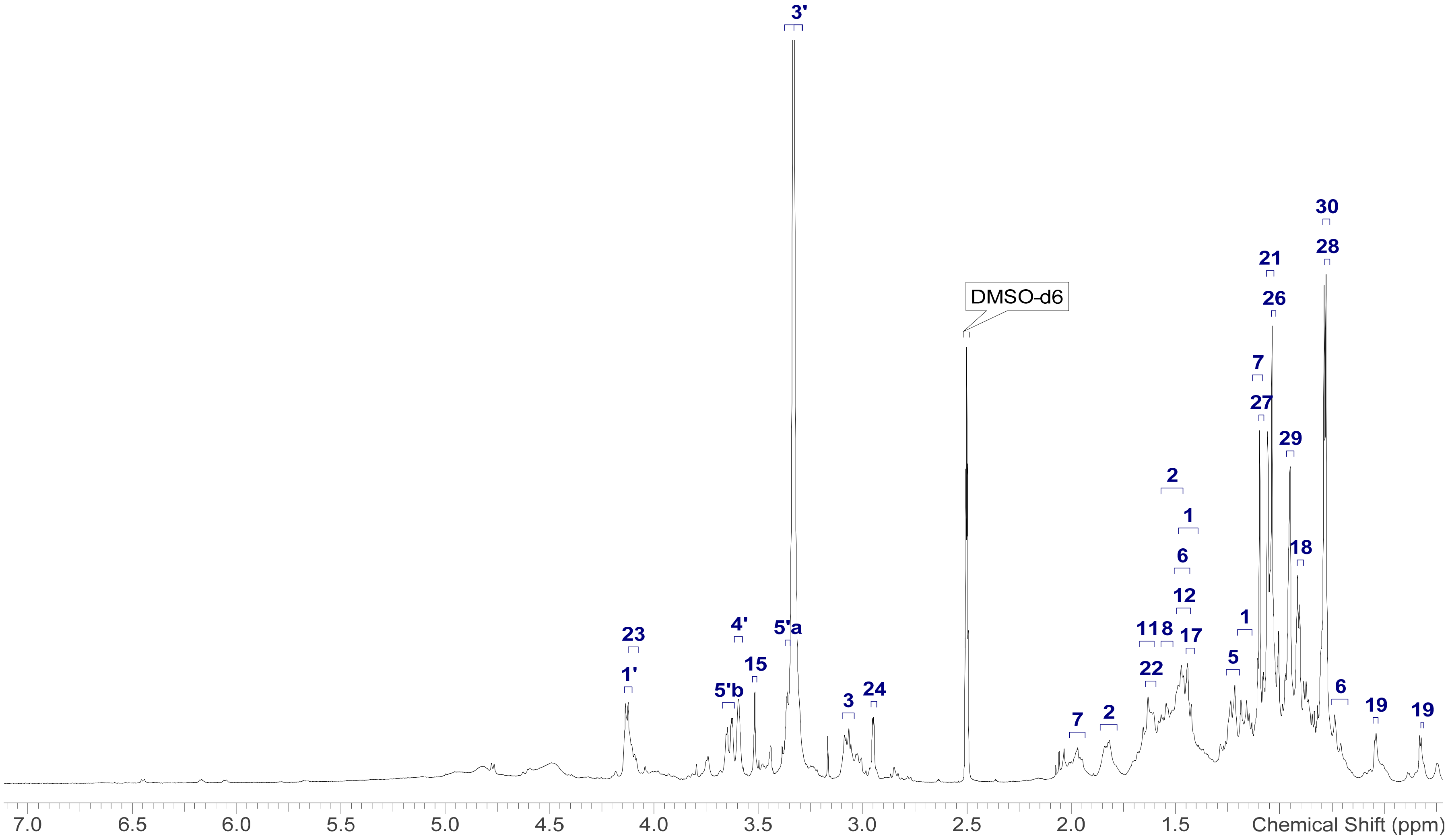

Figure S28. ^{1}H-NMR spectrum of compound **8b** (500 MHz, DMSO-d6).

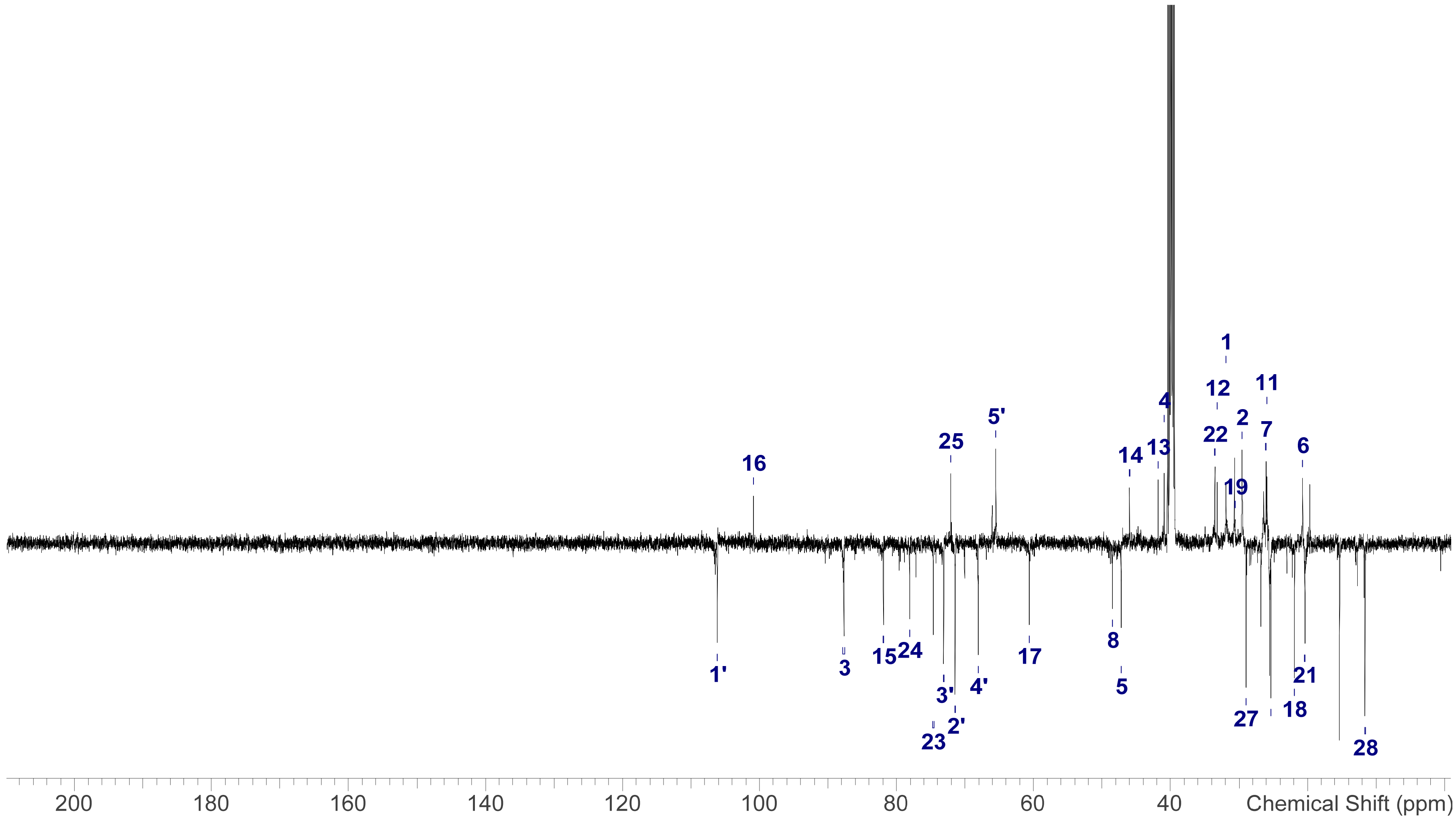

Figure S29. ^{13}C-DEPTq spectrum of compound **8b** (500 MHz, DMSO-d$_6$).

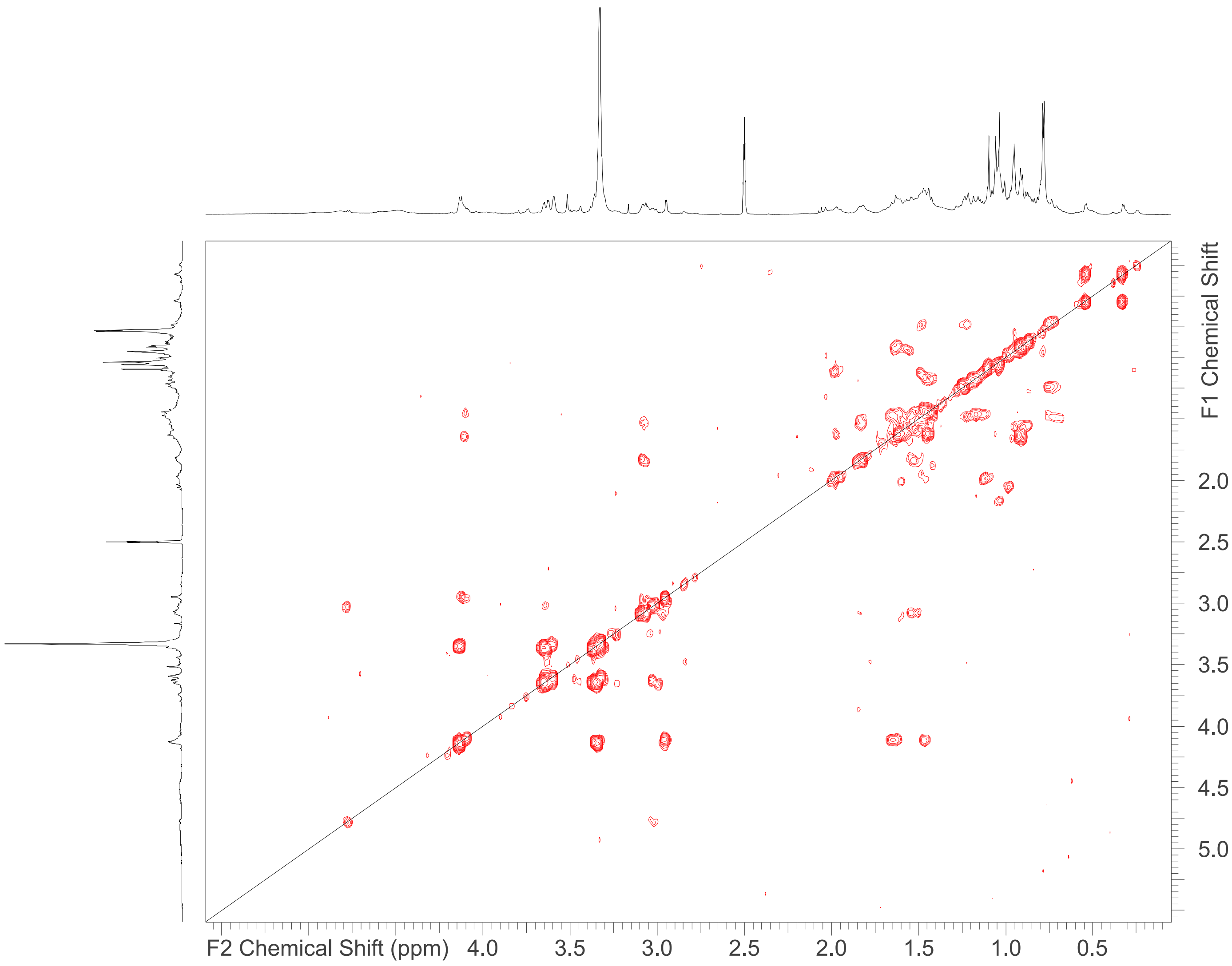

Figure S30. ^{1}H-^{1}H COSY spectrum of compound **8b** (500 MHz, DMSO-d_6).

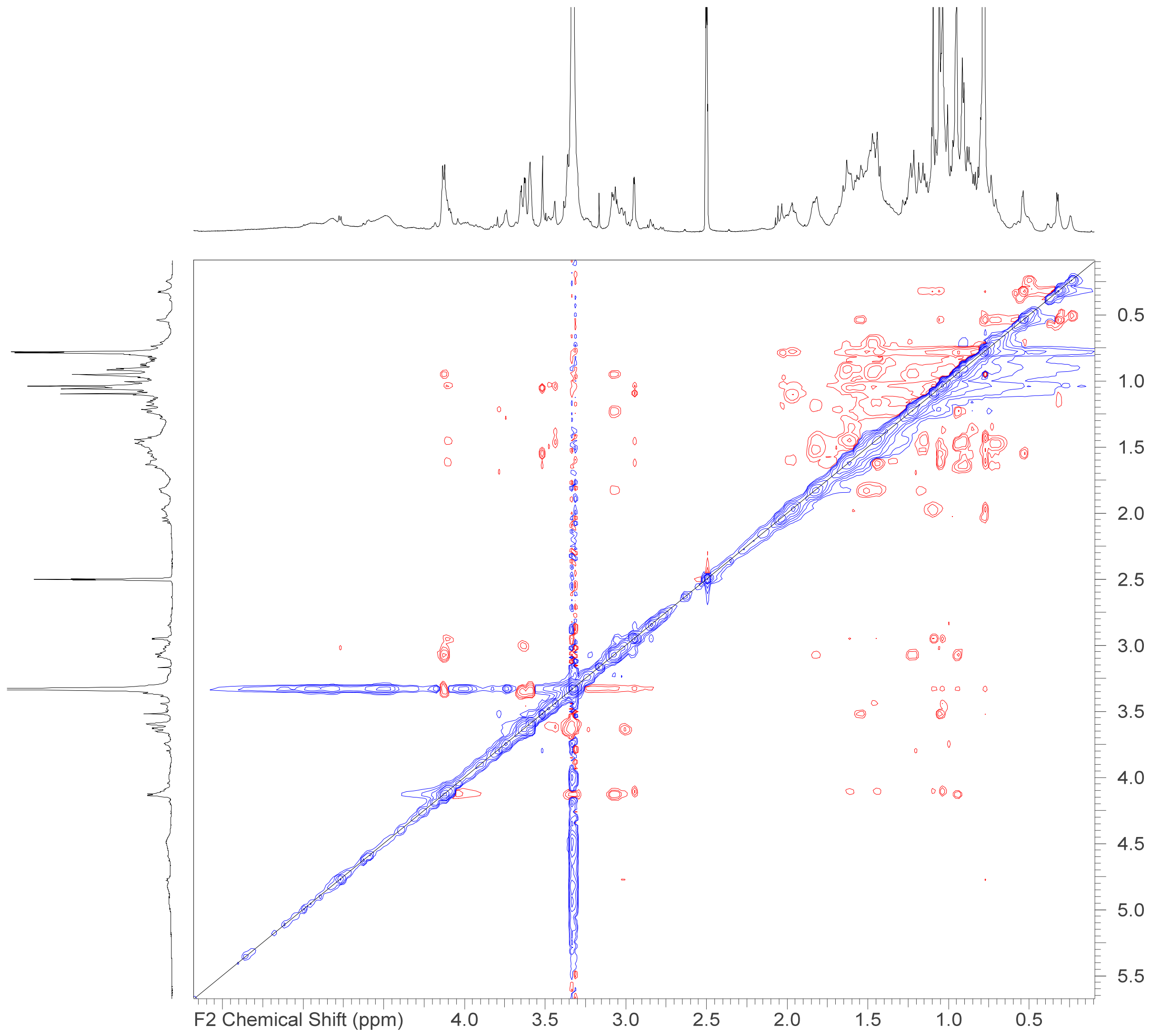

Figure S31. ^{1}H-^{1}H ROESY spectrum of compound **8b** (500 MHz, DMSO-d$_6$).

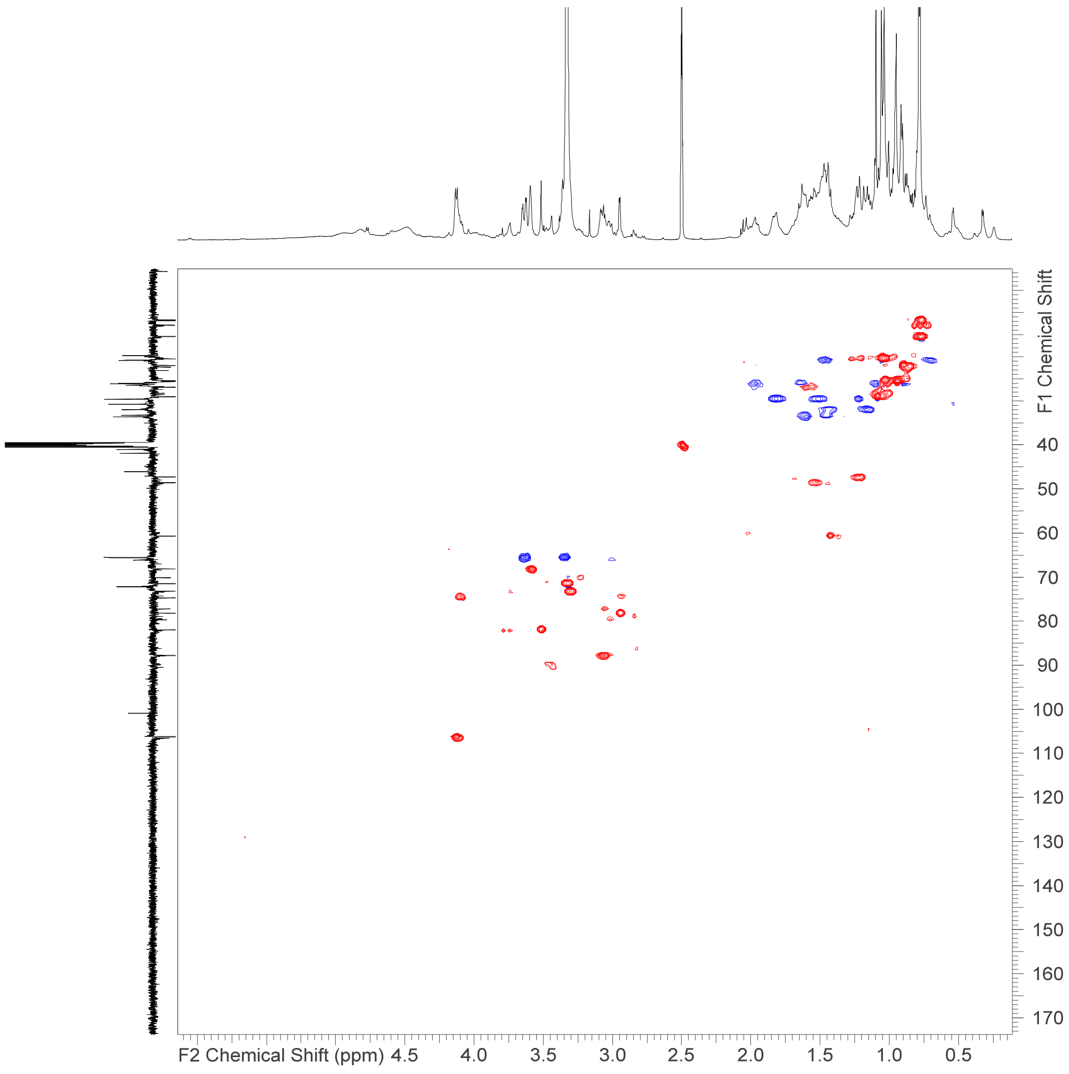

Figure S32. HSQC-DEPT spectrum of compound **8b** (500 MHz, DMSO-d_6).

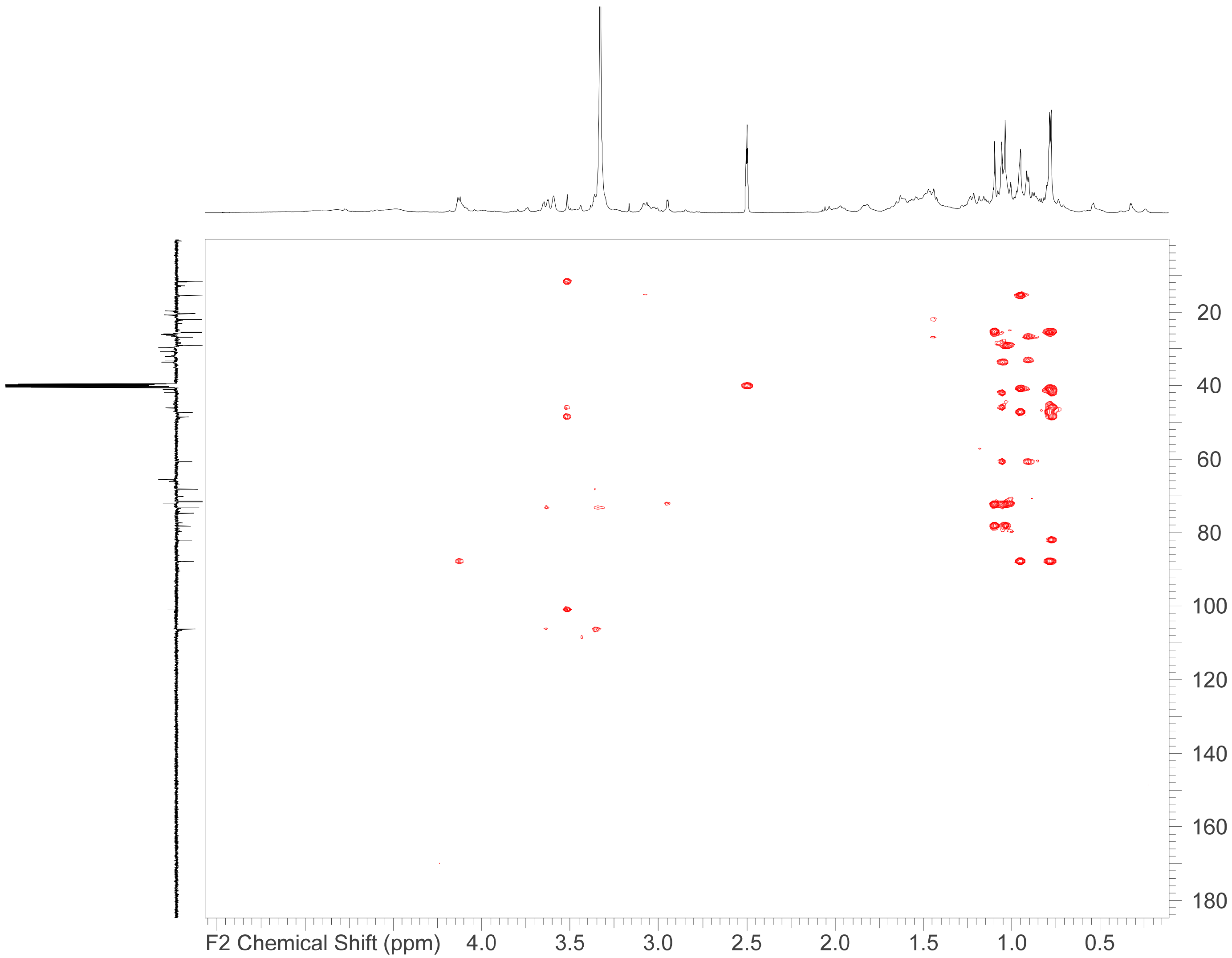

Figure S33. HMBC spectrum of compound **8b** (500 MHz, DMSO-d_6).

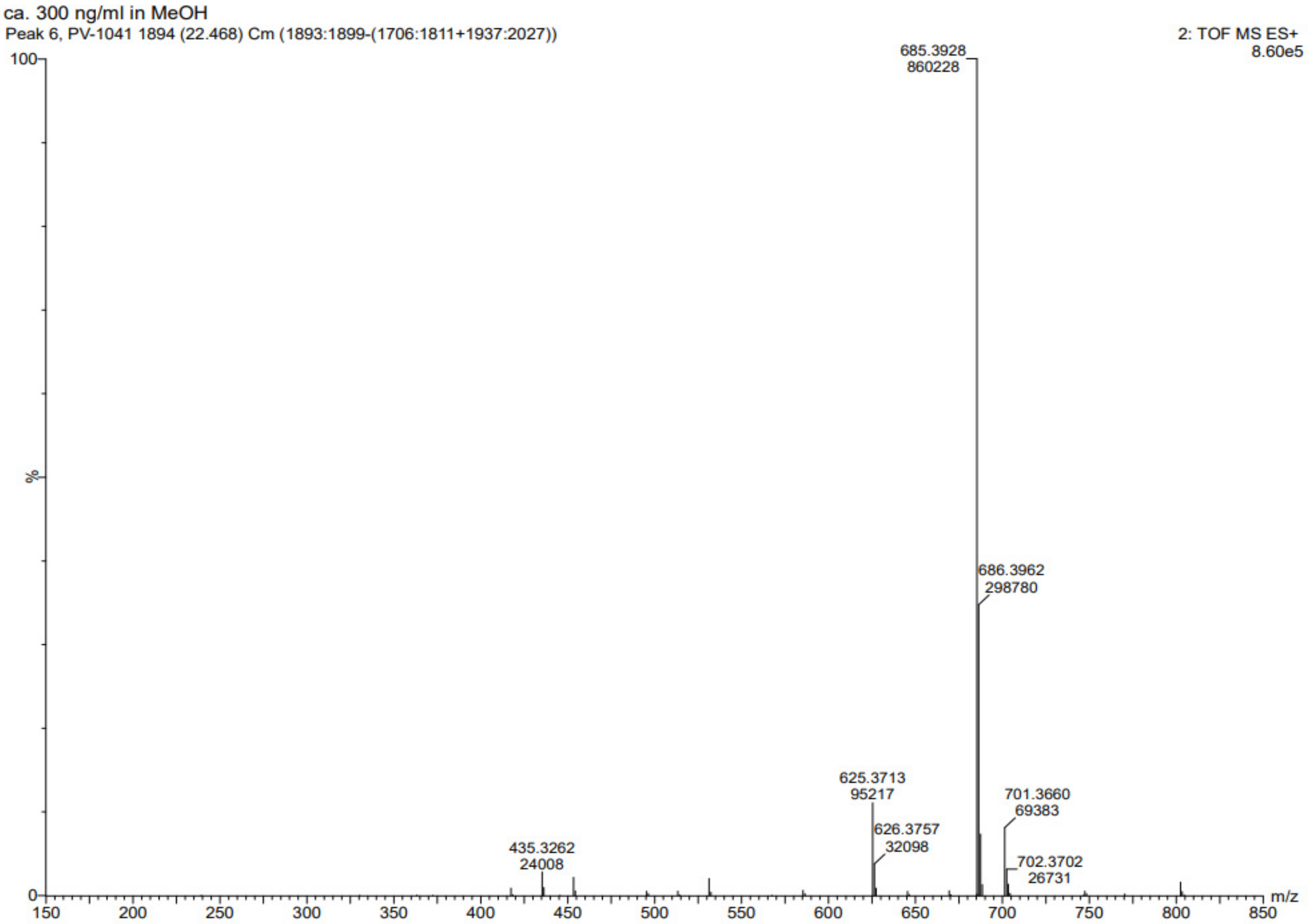

Figure S34. HRESIMS spectrum of compound **5b**.

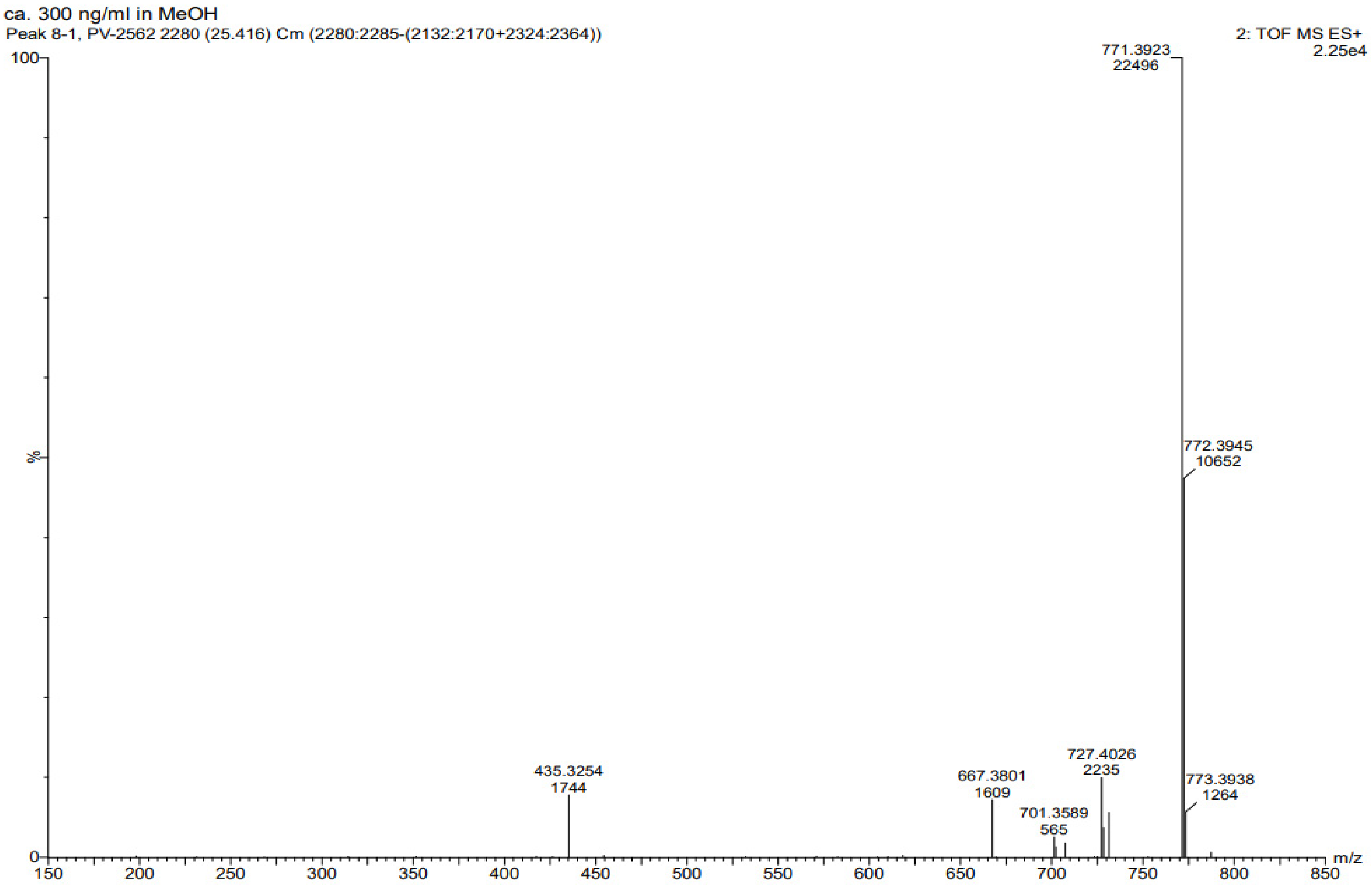

Figure S35. HRESIMS spectrum of compound **8a**.

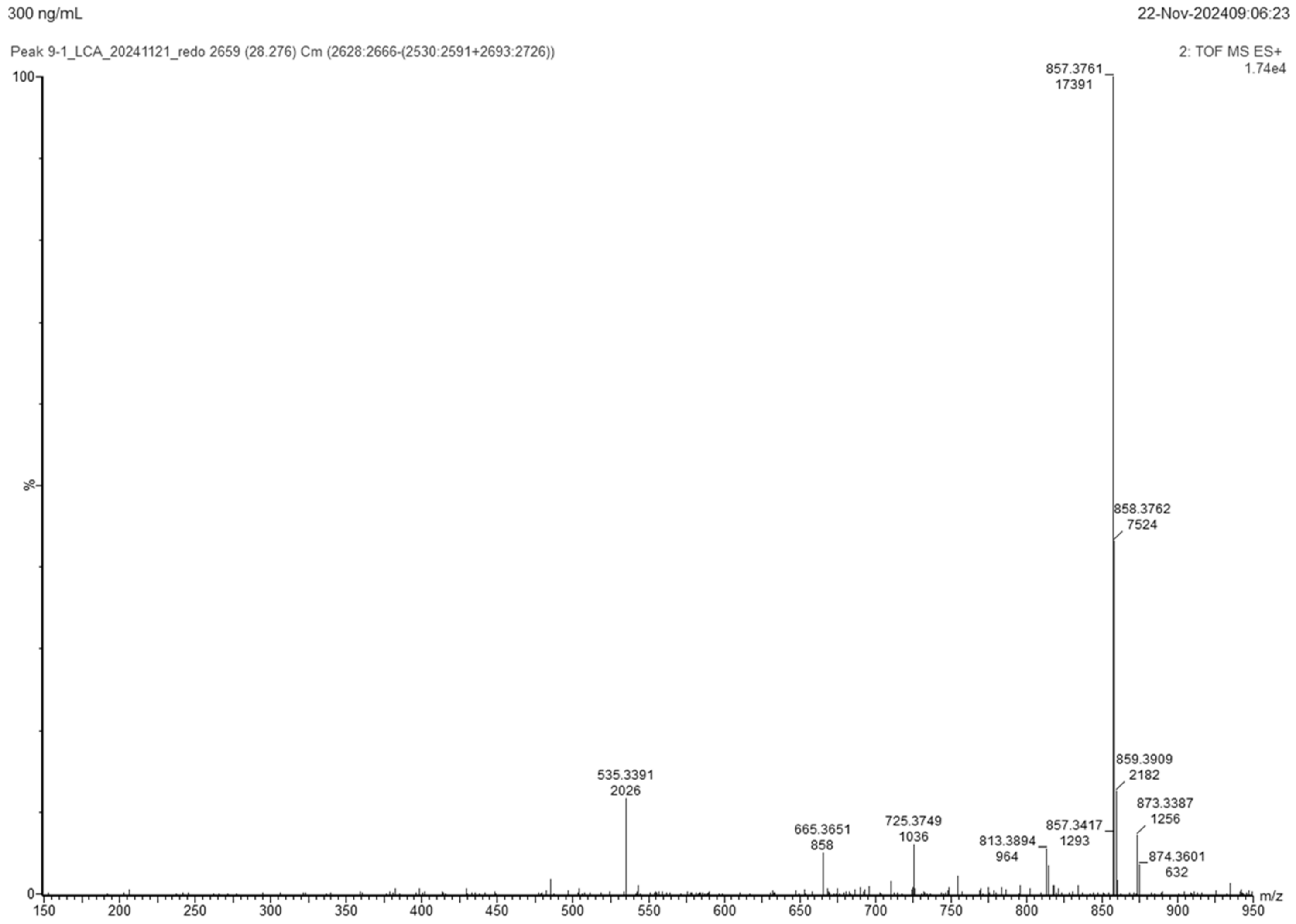

Figure S36. HRESIMS spectrum of compound **9a**.

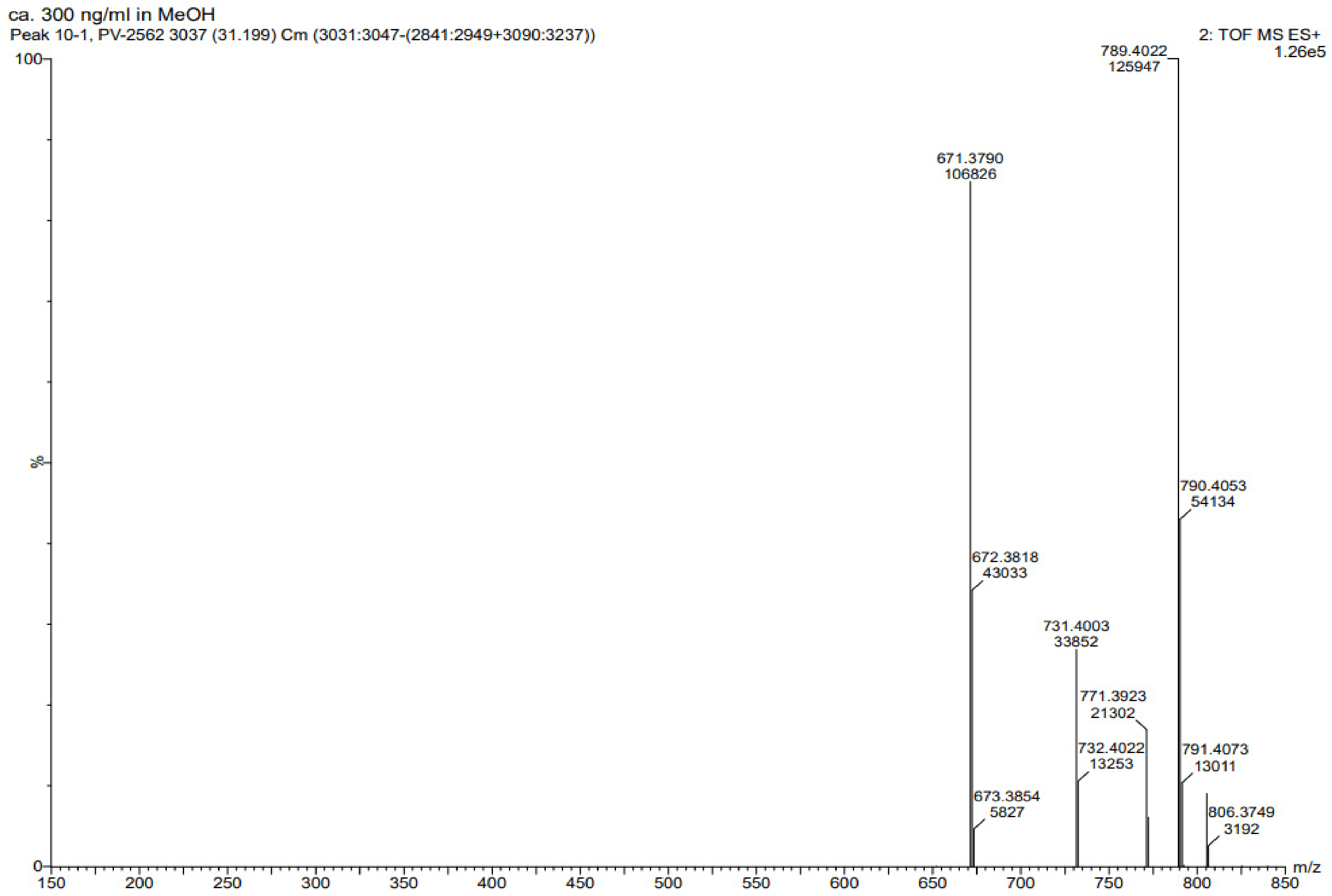

Figure S37. HRESIMS spectrum of compound **10a**.

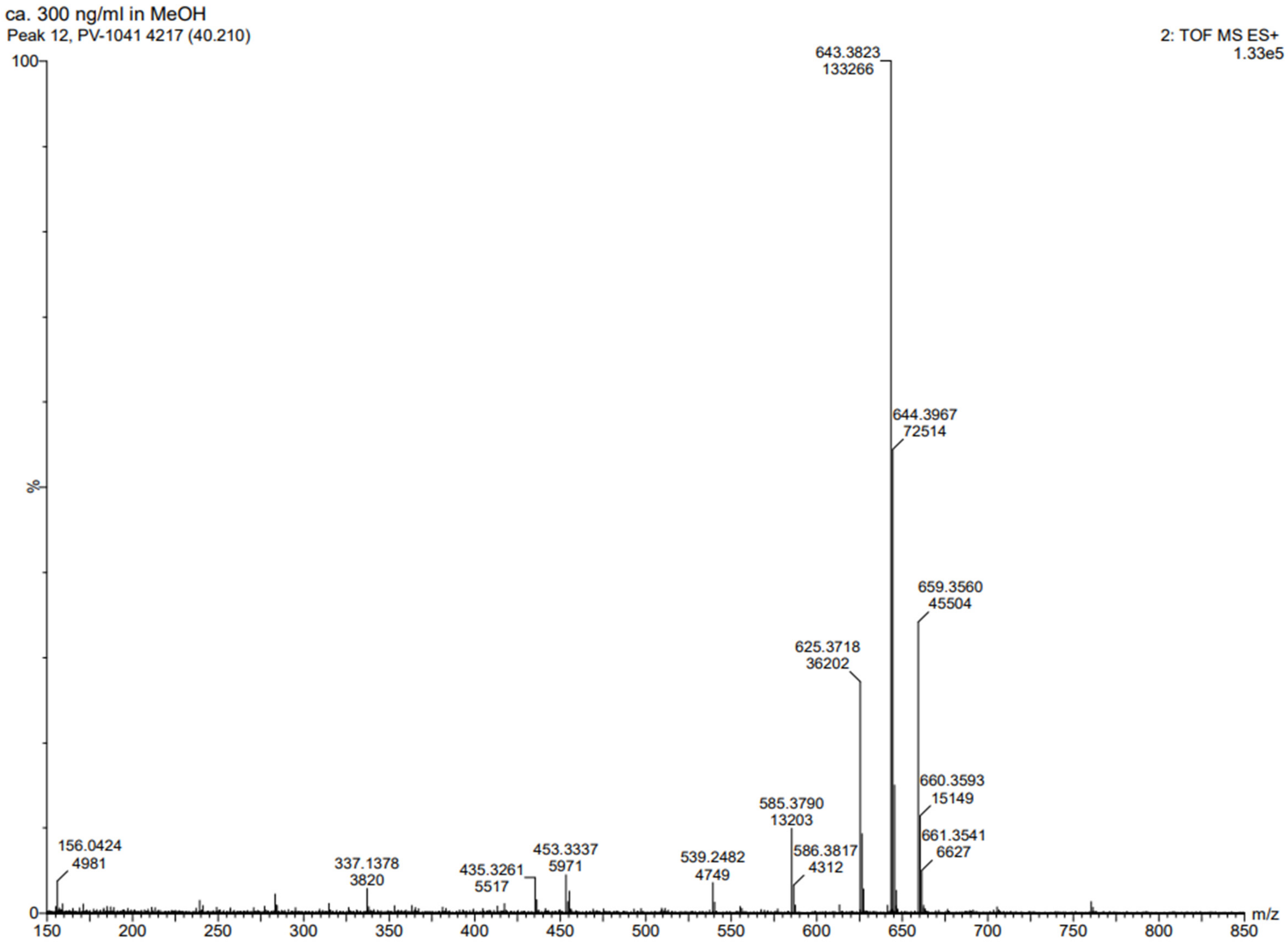

Figure S38. HRESIMS spectrum of compound **13**.

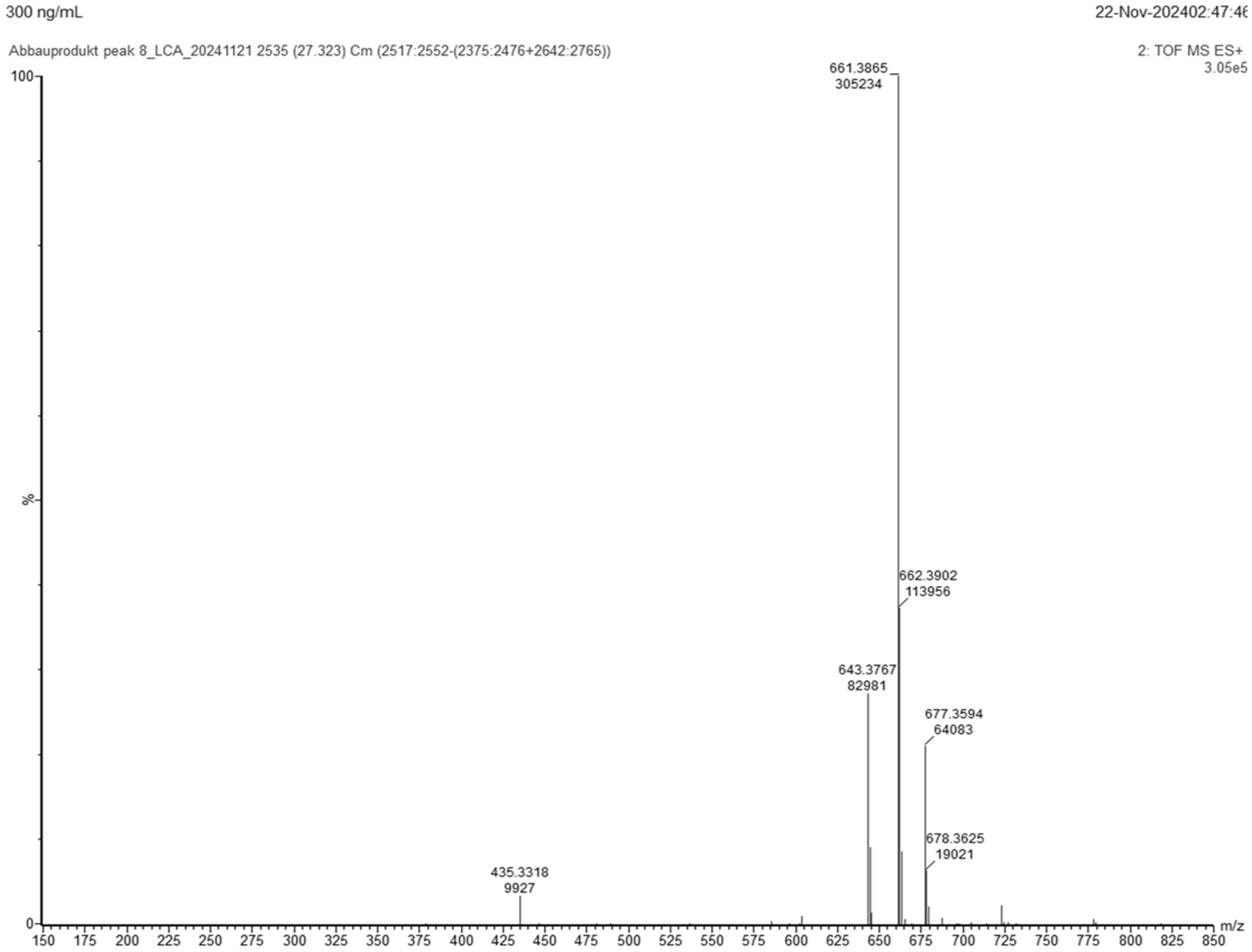

Figure S39. HRESIMS spectrum of compound **8b**.

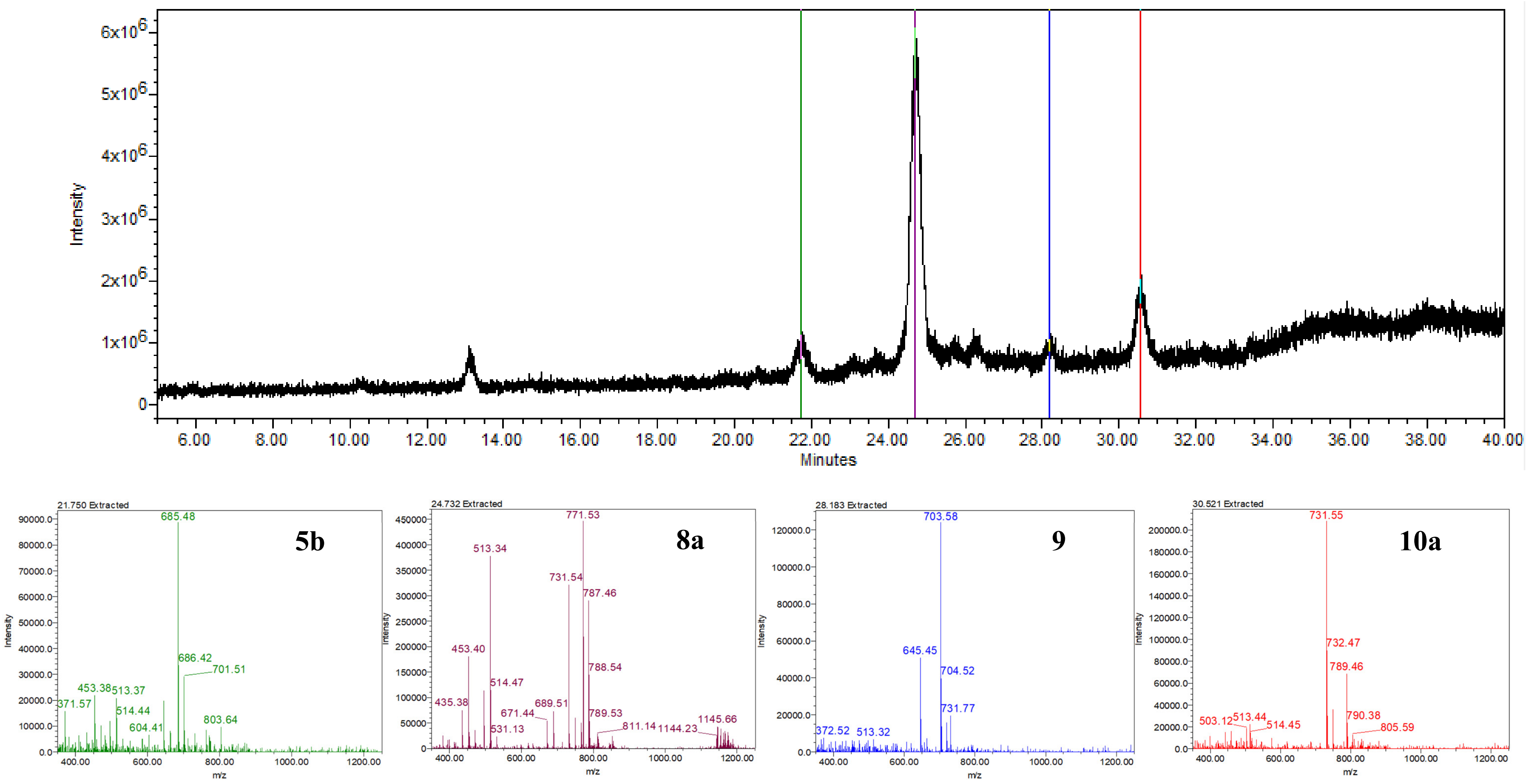

Figure S40. TIC (+) Chromatogram of compound 8a with the degradation products **5b, 9** and **10a**.

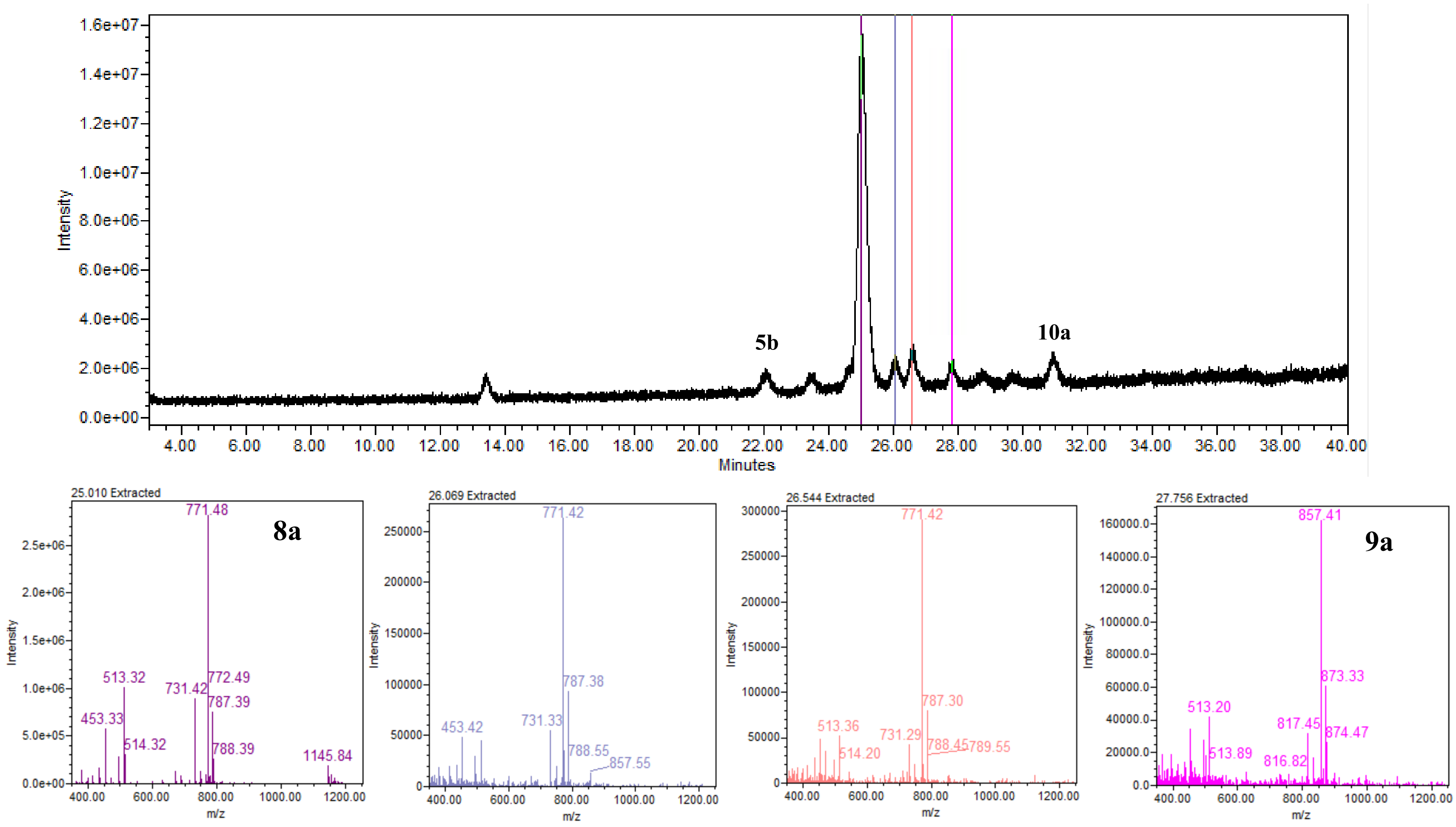

Figure S41. TIC (+) Chromatogram of compound **8a** containing its malonyl positional isomers (RT 26.069 min and RT 26.544) and also compound **9a**.

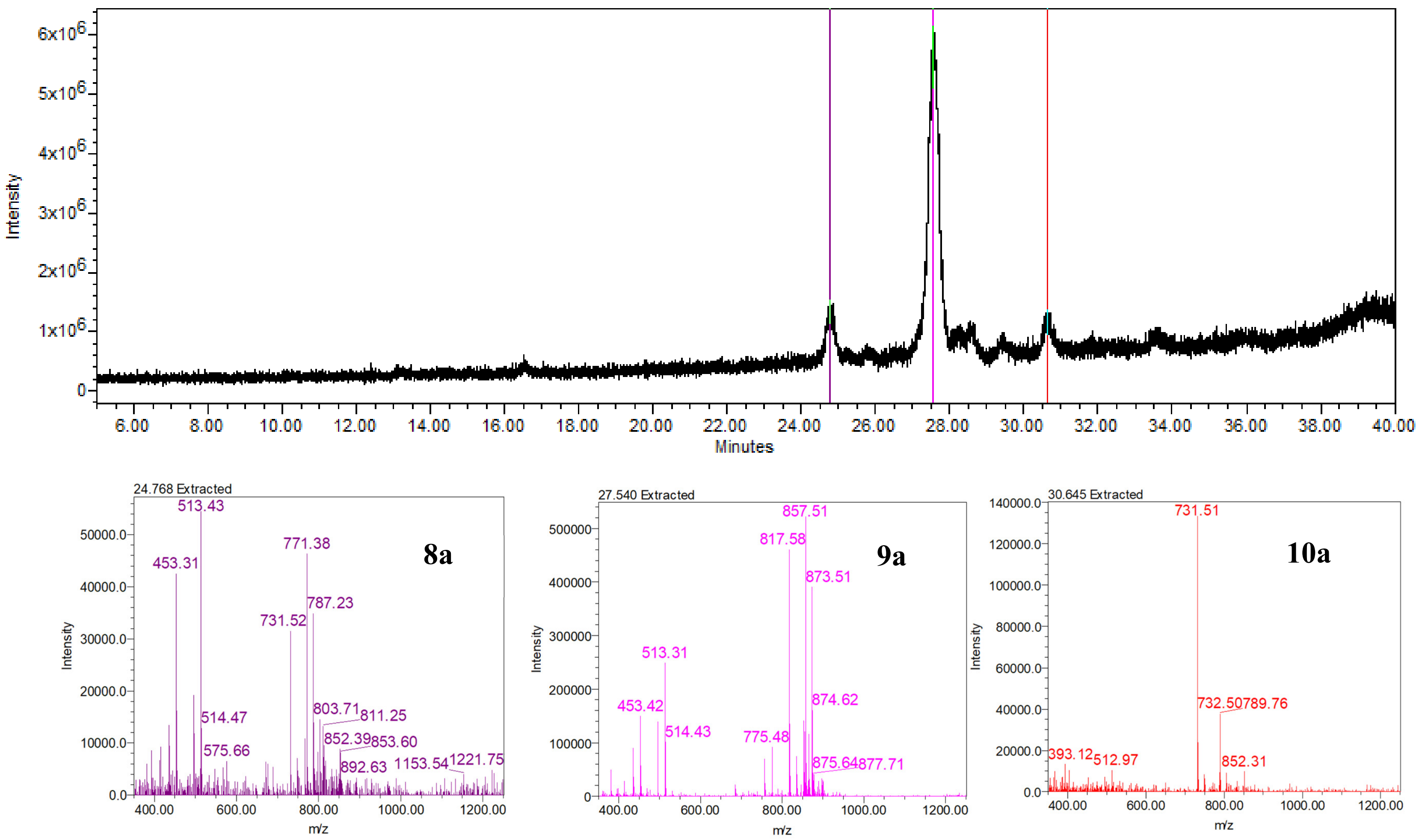

Figure S42. TIC (+) Chromatogram of compound **9a** with the degradation products **8a** and **10a**.

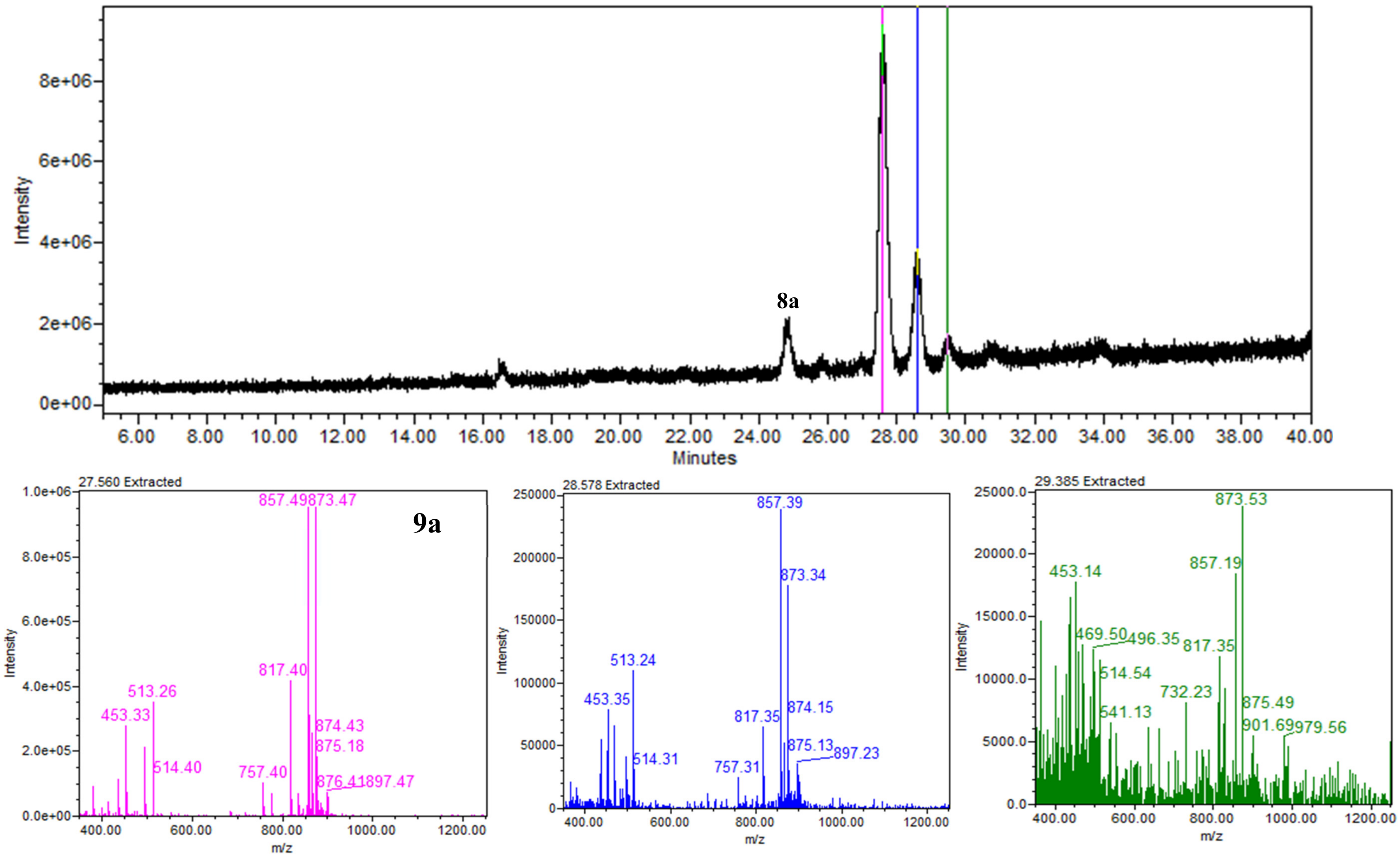

Figure S43. TIC (+) Chromatogram of compound **9a** containing its malonyl positional isomers (2′,3′- and 3′,4′-) as well as compound **8a**.

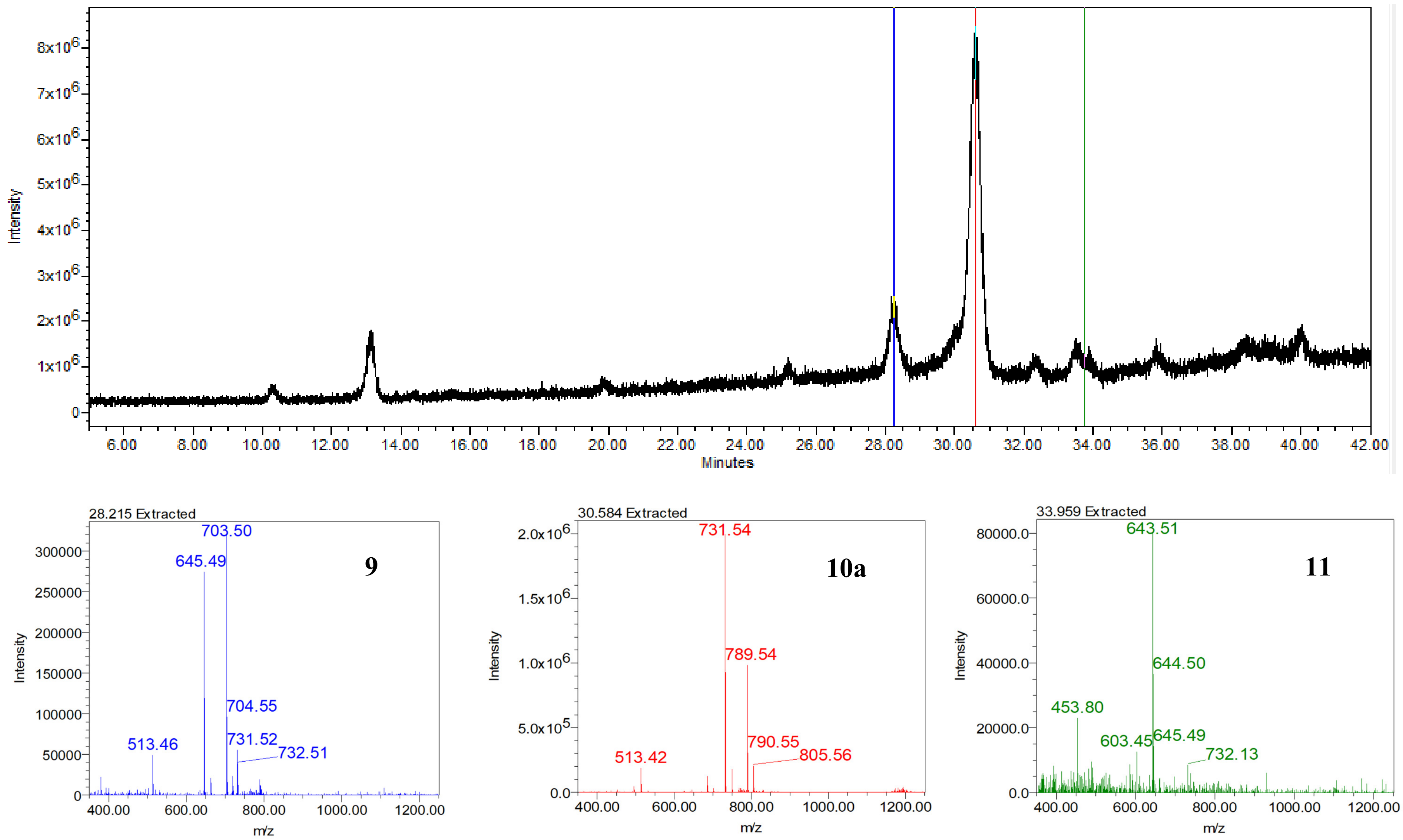

Figure S44. TIC (+) Chromatogram of compound **10a** with the degradation products **9** and **11**.

Figure S45. Key HMBC and ROESY correlations of **5b**, **8a**, **9a**, **10a, 13** and **8b**. HMBC correlations are shown in green and ROESY correlations in violet.

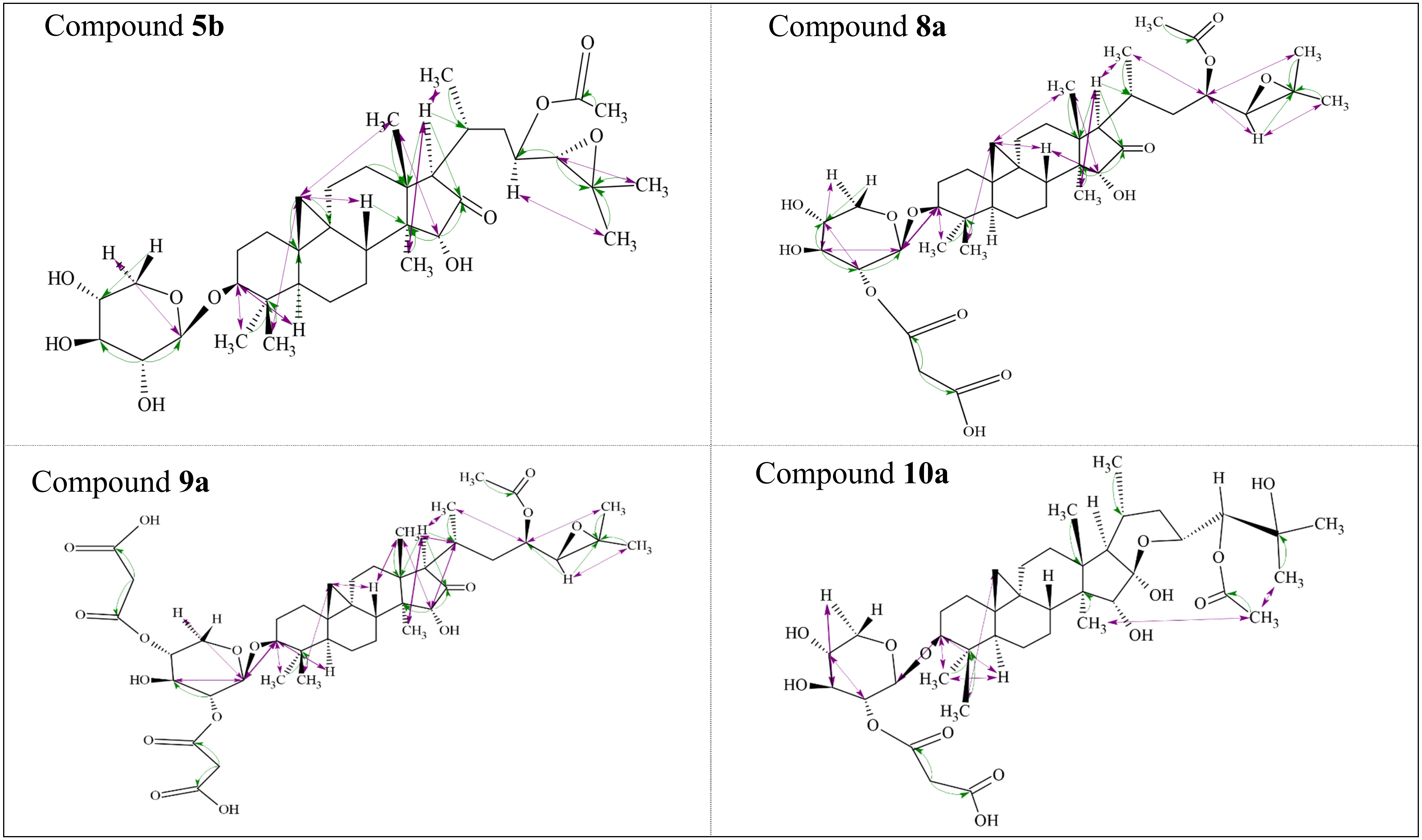

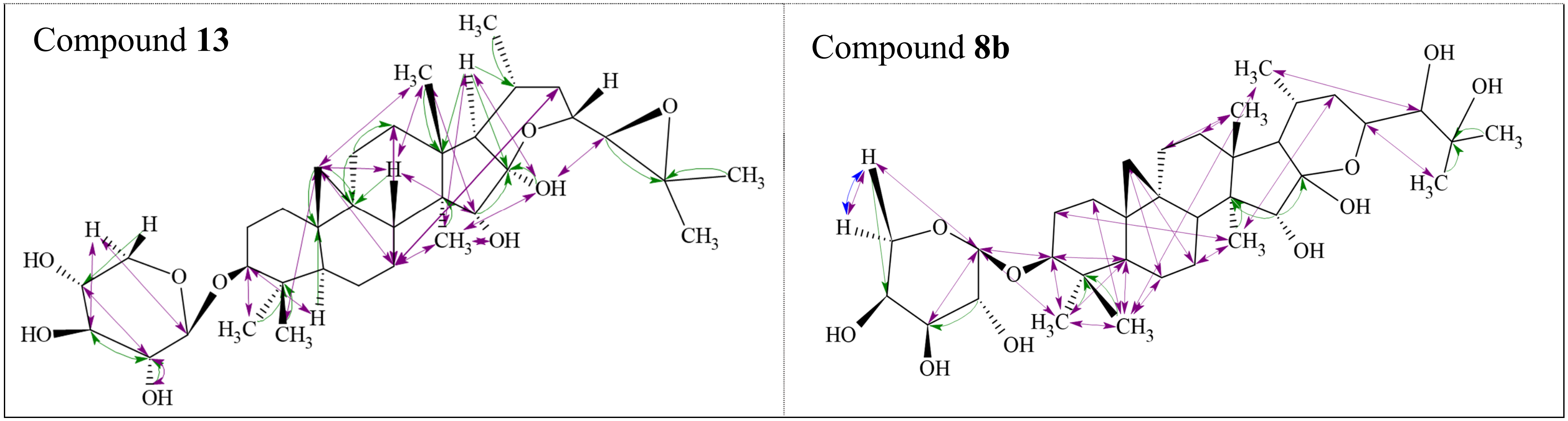
Compound **13**
Compound **8b**
